# Spectroscopically Orthogonal Labelling to Disentangle Site-Specific Nitroxide Label Distributions

**DOI:** 10.1007/s00723-023-01611-1

**Published:** 2023-09-24

**Authors:** Valentina Vitali, Katrin Ackermann, Gregor Hagelueken, Bela E. Bode

**Affiliations:** 1 EaStCHEM School of Chemistry, Biomedical Sciences Research Complex, and Centre of Magnetic Resonance, University of St Andrews, North Haugh, St Andrews, KY16 9ST Scotland; 2 Magnetic Resonance Center (CERM), University of Florence, Via Luigi Sacconi 6, 50019 Sesto Fiorentino, Italy; 3 Department of Chemistry “Ugo Schiff”, University of Florence, Via Della Lastruccia 3, 50019 Sesto Fiorentino, Italy; 4 Institute of Structural Biology, Biomedical Center, University of Bonn, Venusberg-Campus 1, 53127 Bonn, Germany

## Abstract

Biomolecular applications of pulse dipolar electron paramagnetic resonance spectroscopy (PDS) are becoming increasingly valuable in structural biology. Site-directed spin labelling of proteins is routinely performed using nitroxides, with paramagnetic metal ions and other organic radicals gaining popularity as alternative spin centres. Spectroscopically orthogonal spin labelling using different types of labels potentially increases the information content available from a single sample. When analysing experimental distance distributions between two nitroxide spin labels, the site-specific rotamer information has been projected into the distance and is not readily available, and the contributions of individual labelling sites to the width of the distance distribution are not obvious from the PDS data. Here, we exploit the exquisite precision of labelling double-histidine (dHis) motifs with Cu^II^ chelate complexes. The contribution of this label to the distance distribution widths in model protein GB1 has been shown to be negligible. By combining a dHis Cu^II^ labelling site with cysteine-specific nitroxide labelling, we gather insights on the label rotamers at two distinct sites, comparing their contributions to distance distributions based on different in silico modelling approaches and structural models. From this study, it seems advisable to consider discrepancies between different in silico modelling approaches when selecting labelling sites for PDS studies.

**Supplementary Information:**

The online version contains supplementary material available at 10.1007/s00723-023-01611-1.

## Introduction

Understanding topologies and conformational transitions of biomacromolecules and their role in health and disease has spurred a growing scientific interest in pulse dipolar electron paramagnetic resonance spectroscopy (PDS) as a reliable source for structural insights into proteins [1–3] and nucleic acids [4–6]. PDS is often combined with other biophysical methods, including X-ray crystallography, cryo-electron microscopy, or Nuclear Magnetic Resonance (NMR) spectroscopy. PDS derives its strength, efficacy, and versatility from depending solely on the presence of paramagnetic centres, while being unaffected by the complexity or the size of the diamagnetic part of the system under investigation.

These paramagnetic probes are typically attached to biomacromolecules through Site-Directed Spin Labelling (SDSL), an approach pioneered during the late 1980s by Hubbell and co-workers [7–9], based on the introduction of small spin-bearing molecules at specific and strategically placed sites within the native diamagnetic system. PDS is employed to retrieve the magnetic dipole–dipole interaction between two or more spin labels and has become widely and successfully applied for retrieving interatomic distances in the nanometre range (about 2–10 nm and even beyond) [10, 11]. Obtaining information about the distance distributions between pairs of spin labels is crucial for understanding the structural organization [12, 13], dynamics, and conformational changes [14–18] of a vast array of biomolecules.

**Fig. 1:**
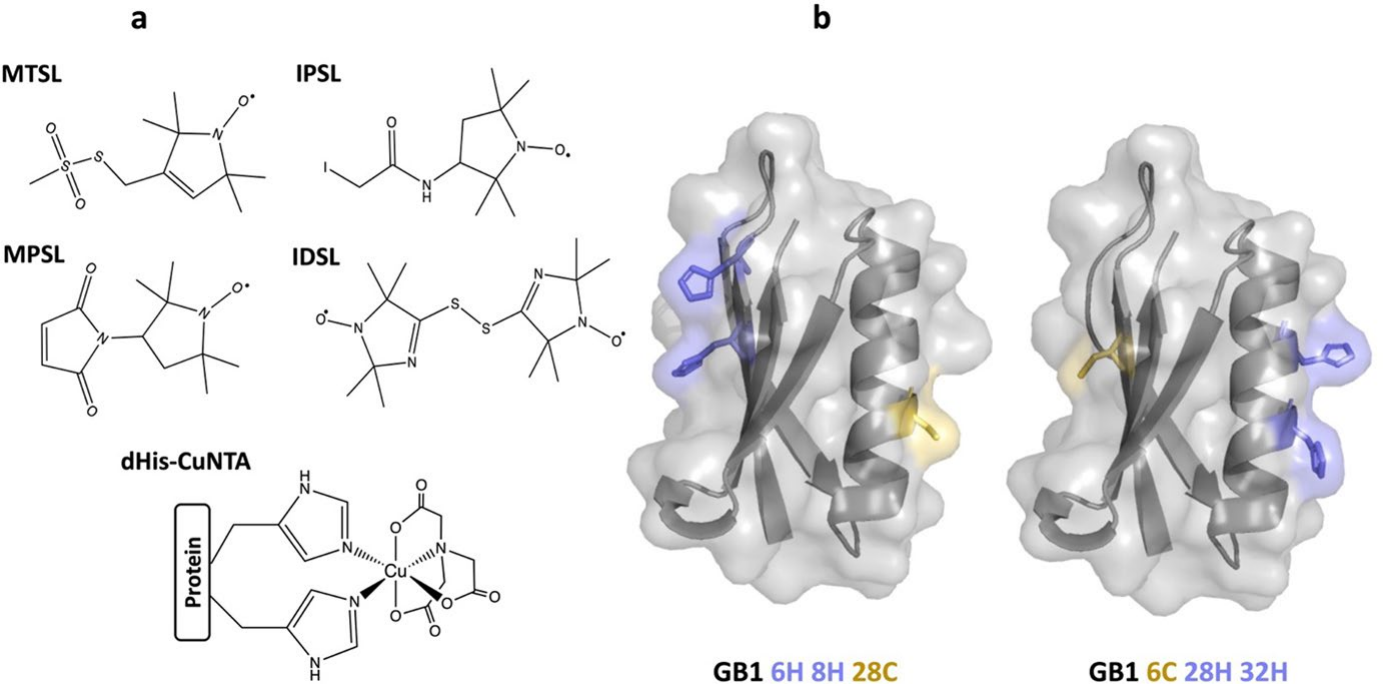
**a** Structures of the four free nitroxide labels used for conjugation of the cysteine residues, respectively, in position 28 (α-helix) and 6 (β-sheet) of the GB1 protein and the CuNTA label directly coordinating the dHis motif (dHis-CuNTA). **b** The two GB1 constructs I6C/K28H/Q32H and I6H/N8H/K28C employed in this study

Depending on the specific case studied, a growing number of spin labels with different properties have been developed. Paramagnetic labels commonly employed for PDS include nitroxides, transition metal ions (such as copper [19, 20] and manganese [21, 22] ions), and lanthanide ions (such as gadolinium) [23, 24], or native metal clusters [25]. Nevertheless, despite this huge variety of types of spin labels, nitroxide radicals are by far the most commonly adopted and are typically attached through covalent bonds to cysteine residues introduced via SDSL. The unique chemistry of the thiol group of this amino acid and its general low abundance in protein sequences results in a high selectivity of the conjugation reaction. Among the great pool of available nitroxide labels, four have recently been tested in PDS on the model system immunoglobulin-binding B1 domain of group G streptococcal protein G (GB1) with two cysteine mutations (one on the α-helix and the other in the β-sheet) [26]. The four tested labels were: (1-Oxyl-2,2,5,5-tetramethyl-3-pyrroline-3-methyl)methanethiosulfonate (MTSL or MTSSL) [27, 28], 3-Maleimido-2,2,5,5-tetramethyl-1-pyrrolidinyloxy (MPSL) [29–31], 3-(2-Iodoacetamido)-2,2,5,5-tetramethyl-1-pyrrolidinyloxy (IPSL) [32–34], and bis-(2,2,5,5-Tetramethyl-3-imidazoline-1-oxyl-4-yl)disulfide (IDSL) [35–38] (Fig. 1a). Each one possesses distinct reactivity and chemical properties that enable unique applications in probing various aspects of biomolecular systems.

Notably, MTSL is the most robust and widespread used nitroxide label. It can be grafted onto the site of choice with high specificity. MPSL is characterized by a greater conformational flexibility due to the increased length of its linker. It still reacts easily with the target protein and is less prone than MTSL to be reductively cleaved due to the absence of the disulfide bond. On the other hand, IPSL tends to be less reactive and requires higher excess for quantitative labelling [26]. The biradical IDSL has the shortest linker that is least flexible, it is also the least stably attached and requires the largest excess of the four nitroxides in labelling reactions. Almost 30-fold excess of label, with respect to the free cysteines, is required due to the equilibrium constant of the disulfide exchange, between the free label disulfide bond and the disulfide bond between the cysteine and the label, being close to one [26]. In the previous study [26], some of the extracted distance distributions between the two nitroxides were broad or even bimodal; however, in samples doubly labelled with nitroxides, it is not straightforward to assign the contributions of the individual labelling site to the distribution width and shape. However, the bipedal spin labels formed by metal chelate complexes of copper(II) such as copper nitrilotriacetate (CuNTA) [39] or copper iminodiacetic acid (CuIDA) [40] bound to double-histidine (dHis) motifs engineered into specific sites [41] display a much narrower distance distribution in GB1 [20]. As observed for the tetra-histidine GB1 construct (I6H/N8H/K28H/Q32H), the intrinsic rigidity and the lower conformational flexibility of this spin pair ensures narrower and more precise distance distributions between the Cu^II^–Cu^II^ pair with respect to the nitroxide–nitroxide distances, making this motif ideal for an independent investigation of the two nitroxides’ behaviours. Although this labelling alternative is not based on covalent bonding, its robustness against competitor ligands has been tested extensively [42].

Therefore, to better understand the influence, in this case, of the nitroxide labelled secondary structural elements (α-helix *vs.* β-sheet) and their respective behaviours in PDS experiments, we have investigated each site of GB1 independently. Here, we have employed orthogonal labelling, individually combining the aforementioned nitroxides with a dHis motif [41] with bipedal coordination to CuNTA [20, 39] alternately at either labelling site, i.e., in the α-helix or the β-sheet, of GB1 (Fig. 1b).

To reliably translate the spectroscopic information on the labelled samples to the actual protein structure of interest, different approaches to explicitly model the labels onto the proteins have been developed. Modelling the spin label conformers through in silico approaches can, e.g., be useful to better understand the reasons behind the differences in widths and shapes of the distributions of the systems under investigation and to reliably translate distance distribution into conformers or conformer ensembles of proteins. The corresponding computational tools provide means to study spin-labelled biomolecules by modelling the distance distributions between paramagnetic moieties and are often employed to identify suitable labelling sites by performing a systematic scanning of the protein residues.

Here, we have explored the two different in silico labelling approaches MMM [43, 44] and MtsslWizard [45], built on different theoretical concepts. While MtsslWizard is based on the excluded volume by steric clashes between label rotamers and the protein, MMM relies on the Lennard Jones potential energy and intrinsic energies of spin label rotamers. The new modelling package chiLife implements these two approaches in a single software tool [46] and has very recently incorporated the use of bipedal labels [47]. Beyond giving information on the distributions between pairs of nitroxide labels, MMM has recently introduced the possibility to simulate the labelling of a dHis motif with coordination complexes such as CuNTA and to retrieve the corresponding distance distributions [44]. Here, we introduce and test a new implementation for bipedal labels into MtsslWizard. The original version of the software superimposes a model of a spin label onto an amino acid (or nucleobase) residue and generates an ensemble of non-clashing rotamers [45]. In essence, this procedure generates an estimate of the accessible volume of the spin label. While this is straightforward for monopedal labels, such as MTSL, bipedal labels are harder to model. Here, the chi angles cannot be randomly chosen, because the other end of the label must coincide with the main chain atoms of the second labelling site.

We have designed a genetic search algorithm to solve this problem. We found that this algorithm quickly converges to a set of rotamers that connect the two labelling sites. As for the published version of MtsslWizard, parameters such as “tight” or “loose” van-der-Waals cutoff can be selected. In principle, the algorithm should work for any bipedal label. Currently, the parameters for the Cu-based labels in Fig. 1 have been implemented (see supplementary.pse file). The code is freely available on GitHub [48] and as a colab notebook [49]. It will be included in a future update of the MtsslSuite-Server (http://www.mtsslsuite.isb.ukbonn.de/).

When studying structures and conformational flexibilities of proteins by SDSL and PDS, high-quality high-resolution structures or structural models are crucially important. While this is commonly based on experimental structures, recent extraordinary progress in deep learning methods has sparked the development of powerful tools for protein structure prediction. Together with in silico labelling techniques, this can greatly assist in the selection of the labelling sites of a protein for PDS experiments. Herein, we have explored three different structure prediction tools: AlphaFold2, which is based on the predictions of protein folding using network-based models and multiple sequence alignments [50], OmegaFold, which leverages deep learning to predict high-resolution protein structure from a single primary sequence without depending on sequence alignment [51], and ESMFold, which predicts protein folding using large-scale language model on a single primary sequence and demonstrated great runtime efficiency [52]. The outcomes of these prediction tools were compared to the ones obtained using one of the GB1 crystallographic structures (PDB: 4wh4) [20].

From the experimental point of view, the significant spectral separation between dHis-CuNTA and nitroxides makes Relaxation-Induced Dipolar Modulation Enhancement (RIDME) [53–55] the natural choice for investigating the distance distributions between these orthogonal labels. This is owed to the fact that the spectrum of copper(II) as a paramagnetic metal centre is much broader than that of a nitroxide radical. Both spectra are also fully separated (i.e., non-overlapping) at Q-band EPR frequencies. Thus, double-resonance techniques that require selective excitation of both spin centres are only feasible with very large bandwidth not available in most EPR resonators. Single frequency techniques that require the excitation of both spins are even less feasible as the excitation would require covering both spectra at the same time. In RIDME, however, the fast-relaxing spin is inverted statistically by longitudinal relaxation introducing the dipolar interaction. Thus, the microwave pulses only need to excite a sufficient fraction of the nitroxide radical to detect the echo which is readily achieved in most setups. By analyzing the modulation with the dipolar frequency, the inter-spin distance can be recovered. RIDME has the further advantage that orientation-dependent excitation of the broad copper(II) spectrum can be avoided as all orientations will contribute, simplifying analysis and interpretation [56]. Here, we compare the 5-pulse variable-time (vt) RIDME, recently introduced by our group [57], with the standard 5-pulse constant-time (ct) RIDME. Testing the vtRIDME sequence further will allow evaluating its performance compared to the constant-time sequence, to gain a deeper understanding of its potential and limitations.

## Experimental Section

### Expression, Purification, and Spin Labelling

The model protein used for this study is the small and rigid immunoglobulin-binding B1 domain of group G streptococcal protein G (GB1). Both GB1 constructs investigated here have a cysteine for the nitroxide labelling and a dHis motif for the chelator agent CuNTA. The I6C/K28H/Q32H construct has the cysteine residue in the β-sheet site, while the I6H/N8H/K28C construct has it in the α-helix. Both constructs were expressed and purified as previously described [19]. The nitroxide spin labels used in this study are MTSL [(1-Oxyl-2,2,5,5-tetramethyl-3-pyrroline-3-methyl)methanethiosulfonate; Santa Cruz Biotechnology], MPSL (3-Maleimido-2,2,5,5-tetramethyl-1-pyrrolidinyloxy; Santa Cruz Biotechnology), IPSL (3-(2-Iodoacetamido)-2,2,5,5-tetramethyl-1-pyrrolidinyloxy; Sigma-Aldrich), and IDSL (bis-(2,2,5,5-Tetramethyl-3-imidazoline-1-oxyl-4-yl)disulfide; Noxygen). These nitroxides have already been employed in our previous study where we have developed the respective labelling protocols [26], which were followed in the current study apart from two adjustments regarding the molar ratio of label to cysteine. Here, we have used 15:1 label:cysteine for IPSL and 25:1 label:cysteine for IDSL, with the aim of maximizing their labelling efficiency, while keeping ratios for MPSL and MTSL the same as used previously at 3:1 label:cysteine [26]. The labelling reaction was carried out in phosphate buffer (42.4 mM Na_2_HPO_4_, 7.6 mM KH_2_PO_4_, 150 mM NaCl, and pH 7.4) as described [26].

Successful spin labelling was confirmed via electrospray ionisation (ESI) mass spectrometry using the in-house mass spectrometry facility. Unlabelled (control) and labelled I6H/N8H/K28C and I6C/K28H/Q32H GB1 samples were diluted to 1 µM in 1% formic acid (FA). 30 µL (30 pmol) per sample were injected onto the liquid chromatography (LC) system (Waters Xevo G2 TOF MS with Acquity HPLC) using a MassPrep cartridge column (Waters), applying a 5 min gradient from 95% water, 5% acetonitrile to 5% water, and 95% acetonitrile (eluents supplemented with 1% FA). Data were collected in positive mode from 500 to 2500 m/z, and charged ion series deconvolution to 0.1 Da resolution was performed using the MaxEnt I algorithm utilising a peak width at half height of 0.4 m/z. Results are shown in the supplementary information (SI) (Fig. S1, Table S1).

Labelling efficiency was confirmed with room-temperature continuous wave (CW) EPR. CW EPR experiments were performed using a Bruker EMX 10/12 spectrometer equipped with an ELEXSYS Super Hi-Q resonator at an operating frequency of ~ 9.9 GHz (X-band) with 100 kHz modulation. 20 μL of every GB1 sample, each with a concentration around 30 μM, were filled into capillary tubes and the CW spectra were recorded with a field sweep of 150 G, a center field of 3505 G, and a modulation amplitude of 0.7 G. All spectra were recorded with a receiver gain of 70 dB and 1 mW power (23 dB attenuation). The double integral of each spectrum was compared with the one of a standard TEMPO sample at 100 μM concentration in water, retrieving the labelling efficiency of each nitroxide spin label for both GB1 constructs (Fig. S2, Table S2). High labelling efficiency, around 100%, was consistently observed for both GB1 constructs and for all the four distinct nitroxide labels after the overnight incubation (with the exception of MTSL for the 6C GB1 construct) as corroborated both by CW EPR and ESI-MS data. Some of the labels, especially IDSL, manifested a labelling efficiency above 100%. However, due to the low concentration of the samples, these values can be considered within the error range.

### PDS Sample Preparation

Spin-labelled samples were freeze-dried and resuspended in D_2_O (Merck). CuNTA stock solution (10 mM in phosphate buffer) was added to each sample to yield a final concentration of 10 μM CuNTA for the I6C/K28H/Q32H construct and 50 μM CuNTA for the I6H/N8H/K28C GB1 construct to ensure a final loading of the dHis motif of above 95%. The difference in the desired final CuNTA concentration is related to the lower affinity of the copper ligand towards the dHis motif in the β-sheet with respect to the dHis in the α-helix, as discussed previously [19]. Each sample was prepared to a final protein concentration of 10 μM and a total final volume of 65 μL, with 50% (v/v) of deuterated cryo-protectant (ethylene glycol d-6, Deutero GmbH) to ensure the formation of a glassy-frozen solution. All samples were transferred to 3 mm quartz tubes (Technical Glass Products) and immediately frozen by immersion in liquid nitrogen.

### RIDME Measurements

All PDS experiments were performed using a Bruker ELEXSYS 580 pulse EPR spectrometer. Temperatures were maintained using a cryogen-free variable temperature cryostat (Cryogenic Ltd) operating in the 1.8–300 K temperature range. All samples were measured with the 5-pulse constant-time (ct) RIDME [53] and the recently introduced variable-time (vt) RIDME [57] at 30 K, using a high-power 150 W travelling-wave tube (TWT; Applied Systems Engineering) at Q-band (34 GHz) in an overcoupled 3 mm cylindrical resonator (Bruker ER 5106QT-2w in TE102 mode). For every sample, the pulses were applied on the maximum of the nitroxide echo detected field sweep, ctRIDME measurements were performed using the pulse sequence *π*/2–*τ*_1_–*π*–(*τ*_1_ + *t*)–*π*/2–*T*_mix_–*π*/2–(*τ*_2 _− *t*)–*π*–*τ*_2_–echo with detection pulse lengths *π*/2 and *π*, respectively of 8 and 16 ns. Each trace was acquired using an SRT of 10 ms, a *τ*_1_ of 400 ns, and 2 shots-per-loop and 32-step phase cycling.

vtRIDME measurements were performed using the pulse sequence *π*/2–*τ*_1_–*π*–(*τ*_1_ + *t*)–*π*/2–T_mix_–*π*/2–*τ*_0_–*π*–(*τ*_2_ + *t*)–echo with detection pulse lengths *π*/2 and *π*, respectively, of 8 and 16 ns. Each trace was acquired using an SRT of 10 ms, a *τ*_1_ of 400 ns, and 2 shots-per-loop and 32-step phase cycling.

Measurements were recorded with a short (reference) and a long mixing time of 5 and 200 μs, respectively, to allow deconvolution (dividing the constant and variable-time RIDME traces with the longer mixing times by the corresponding reference traces) of the traces [57].

### PDS Data Processing

Data were processed in DeerAnalysis2021 using Tikhonov regularisation and validation as previously described [58, 59]. Briefly, RIDME data were first background-corrected using a homogeneous six-dimensional background function before Tikhonov regularisation followed by statistical analysis varying background start time from 5 to 30% of the total trace length in 8 trials and varying the background dimension from 3 to 6 in 7 trials. Resulting background start time and dimension for the best fit were then used as starting points for a second round of Tikhonov regularization followed by a second round of statistical analysis, this time also including the addition of 50% random noise in 16 trials (896 total trials). Validation trials from the second validation round were pruned with a prune level of 1.15, where trials exceeding the root-mean-square deviation of the best fit by at least 15% are discarded.

### Structure Prediction and Modelling

Four different GB1 structures were compared in this study: a crystallographic structure (PDB: 4wh4) and three predicted structural models obtained from AlphaFold2, OmegaFold, and ESMFold, respectively. The predicted structures were obtained through the use of Google Colabfold [60–63]. The I6C/K28H/Q32H and I6H/N8H/K28C constructs were in silico labelled using the 2021 version of MMM and a version of MtsslWizard with the newly introduced CuNTA labelling implementation [48, 49] based on octahedral coordination of the Cu^II^. Briefly, a model of the bipedal label is superimposed onto the first labelling site and the algorithm generates 8000 trial conformations. For each of these conformations, a penalty in the form of the root-mean-square deviation (rmsd) between the main chain atoms of the second labelling site and the corresponding atoms of the label is calculated. This penalty is used to rank the trial conformations and eliminate the 5% worst conformations. The discarded conformations are replaced by offspring of the remaining conformations and the process is repeated. Both labelling methods were carried out under two different conditions. Modelling with MMM was performed under ambient (298 K) and cryogenic (175 K) temperatures, and labelling with MtsslWizard was carried out with Tight (vdW-restraint 0 clashes, 3.4 Å cutoff) and Loose (vdW-restraint 15 clashes, 2.0 Å cutoff) settings [45].

## Results and Discussion

PDS measurements were performed on the orthogonally spin-labelled GB1 constructs using the 5-pulse constant-time RIDME and the 5-pulse variable-time RIDME experiment, resulting in four different data sets (reference corrected and uncorrected for each, constant- and variable-time RIDME, Figs. S3 and S4) for each of the eight analyzed samples (two GB1 constructs with four nitroxide labels each). As expected for short distances and relatively narrow distance distributions, the experimental data did not display significant discrepancies between these four datasets, showing high consistency and robustness in the distance distributions for both ctRIDME and vtRIDME, regardless of the application or not of the deconvolution step (Fig. S5). In addition, for each of the four traces of every sample, the sensitivity per echo and per unit of time was obtained as previously described [58]. The extracted values confirmed that the vtRIDME gave generally higher sensitivity values than the ctRIDME (for more detailed information, see Tables S3 and S4).

**Fig. 2:**
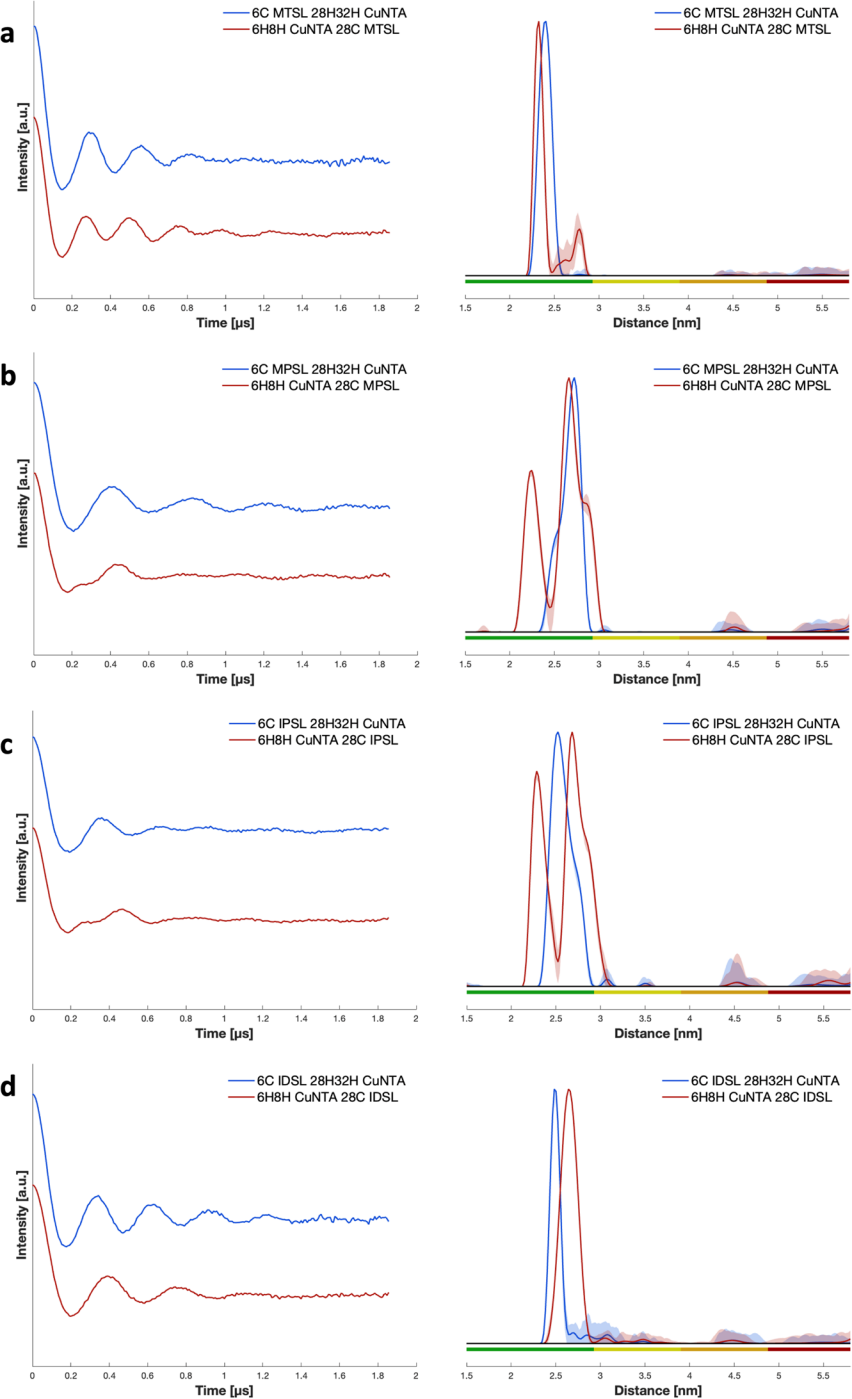
Constant time RIDME traces after background correction with DeerAnalysis2021, for both GB1 constructs I6C/K28H/Q32H (nitroxide on the β-sheet, in blue) and the I6H/N8H/K28C (nitroxide on the α-helix, in red) for the four nitroxide labels, MTSL, MPSL, IPSL, and IDSL, and their respective distance distributions. Colour bars represent reliability ranges (green: shape reliable; yellow: mean and width reliable; orange: mean reliable; red: no quantification possible). **a** MTSL, **b** MPSL, **c** IPSL, and **d** IDSL

The distributions of the experimental data of the GB1 with the nitroxide on the β-sheet site (Fig. 2) were characterized by monomodal and relatively narrow distributions, with hints of a shoulder for the MPSL and IPSL labels. On the other hand, most nitroxide labels on the α-helix displayed a bimodal trend with two distinct and large amplitude peaks arising from the presence of two different sub-ensembles of conformers. These may be rationalised for nitroxides with long enough linkers and discrete flexibility, allowing the label to be pointing away from the protein backbone at the maximum distance possible, but also allowing it to be bent towards the protein surface, resulting in shorter distances. Therefore, having the shortest and least flexible linker, IDSL can be hypothesized to have a low propensity of bending itself towards the protein surface, and was the only spin probe retaining a unimodal and relatively narrow distribution, independent of the site of GB1 to which it was attached. The observed bimodality is very unlikely caused by conformations of GB1 itself as both, the copper(II)–copper(II) distance in GB1 I6H/N8H/K28H/Q32H and the nitroxide-nitroxide distance in bis-IDSL labelled GB1 6C 28C are narrow and monomodal, thus strongly indicating a single GB1 conformation [20, 26].

The presence of a spin probe with low conformational flexibility, such as dHis-CuNTA, was crucial to unravel the individual contributions of the nitroxides on the α-helix and the β-sheet. Drastically reducing the contribution to the width of one labelling site to the distance distribution allowed us to untangle the ambiguity observed in the double cysteine GB1 construct I6C/K28C [26] (Fig. S6). Surprisingly, the helix site, as a well-studied structural element which is often the preferred choice for attaching paramagnetic moieties, seemed to be the one entailing the bimodality and broadness trend of the distance distribution peaks for the system under investigation. Nonetheless, the intrinsic rigidity exhibited by the CuNTA label does not translate in discernible orientation selection effects that would impact the distance distribution shape, since in the RIDME pulse sequence, the Cu(II) is not excited by any pulses and relaxes isotropically to good approximation.

Here, we have introduced a new implementation for MtsslWizard, which can now predict distance distributions between nitroxides and dHis copper(II) labels, and we compared its performance with the ones achieved from MMM [44], to test whether, in this case, the experimental bimodality could be captured reproducibly by either or both of the in silico tools.

The in silico labelling was performed on four different model structures: the crystallographic structure (PDB: 4wh4) and three structures generated from the prediction suites AlphaFold2, OmegaFold, and ESMFold. Interestingly, a recent publication on GB1 claimed that in their research, AlphaFold2 was the best structural model in the prediction of the experimental behaviour of a dHis GB1 system [64]. While this seems counterintuitive, it may well be that the AI-based prediction performs best as it has been trained using a large number of datasets, thereby averaging many structures. Therefore, here, we discuss in depth the results obtained based on the AlphaFold2 structure, while data for the other models can be found in the SI (Tables S8–S11, Fig. S7–S10). Since no substantial variations in terms of shape, mean, width, and full width at half maximum (FWHM) of the distance distributions obtained from the vt or ct RIDME were observed, we decided to rely on the standard ctRIDME deconvoluted experimental data to compare with the in silico distance distributions. Deconvoluted data for vtRIDME and non-deconvoluted for ctRIDME and vtRIDME are shown in the SI (Fig. S4 and Tables S5–S7).

**Fig. 3:**
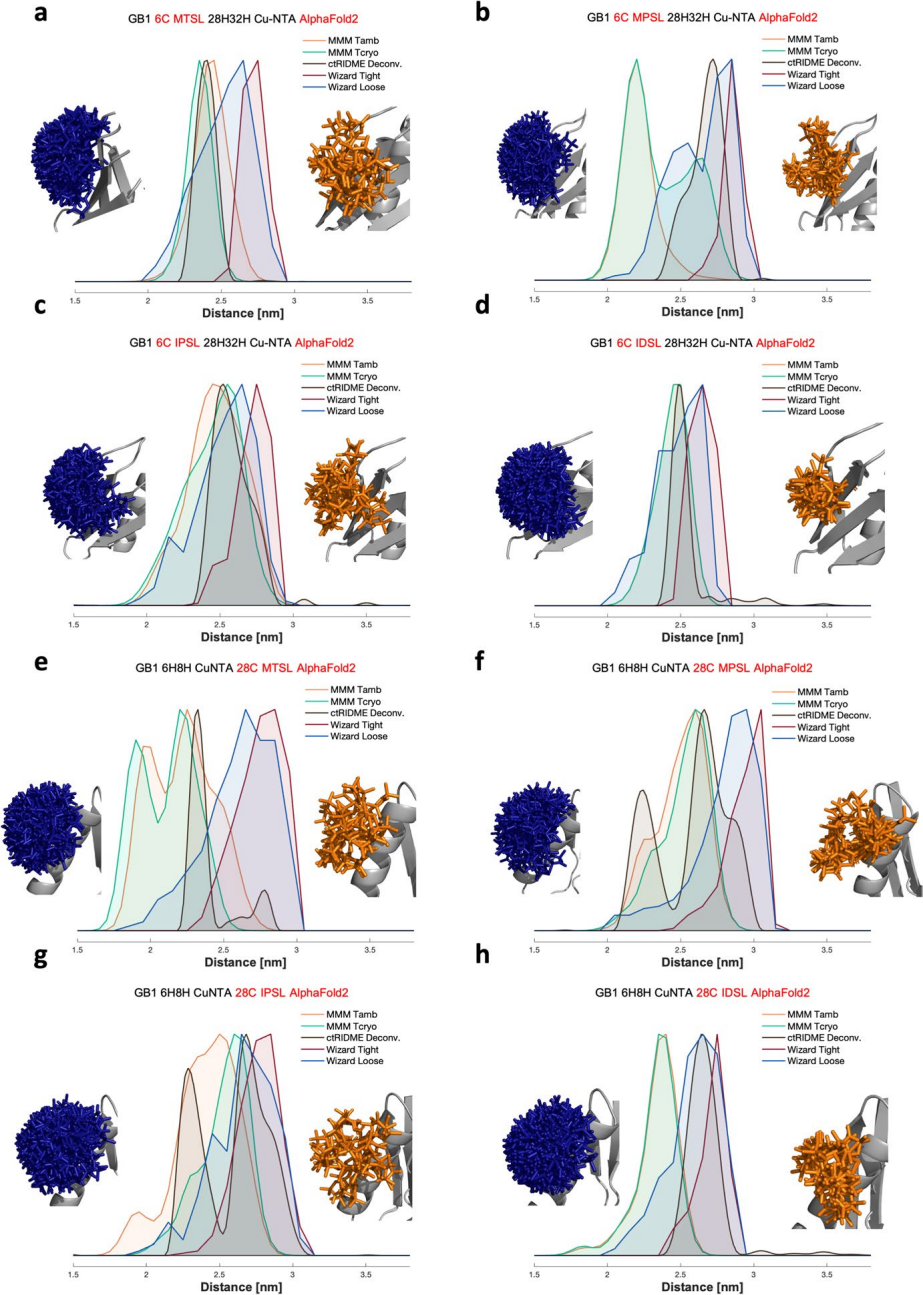
Modelled distance distributions for both GB1 constructs (I6C/K28H/Q32H and I6H/N8H/K28C), both labelled with MTSL, MPSL, IPSL, and IDSL, based on the AlphaFold2 structure, superimposed with their respective experimental distance distributions (derived from ctRIDME deconvoluted data, in black). The in silico approaches compared are MMM at ambient (orange) and cryogenic (green) temperature and MtsslWizard with Tight (red) and Loose (blue) settings**.** Next to the distance distributions label rotamers modelled using MtsslWizard Loose (blue) and MMM at ambient temperature (orange) are given.** a** 6C MTSL, **b** 6C MPSL, **c** 6C IPSL, **d** 6C IDSL, **e** 28C MTSL, **f** 28C MPSL, **g** 28C IPSL, and **h** 28C IDSL

The comparison between the in silico and the experimental distance distributions (Fig. 3) revealed an intriguing difference between MMM and MtsslWizard. While the former consistently underestimated the distances between the two labels, with respect to the experimental data, the latter tended to overestimate the same measurements. In general, both approaches seemed more accurate in the prediction of the narrow and unimodal distributions of the nitroxide labelling on the β-sheet, with the only exception being MMM at cryogenic temperature settings, that simulated hints of bimodality for MPSL. On the other hand, the predicted distributions for nitroxide labelling on the α-helix were characterized by an increased broadness that partially covered the two distinct peaks of the experimental data, although neither MMM nor MtsslWizard seemed to fully and reliably predict the bimodality of the experimental data. Curiously, for MTSL, the shorter distance between the labels, corresponding to the nitroxides interacting with the protein surface, seemed to be better predicted by MMM, while the longer distances seemed to be better predicted by MtsslWizard. This seemed sensible, as MtsslWizard has no attractive energy contribution for conformers close to the protein surface. Rotamers for each nitroxide and for the CuNTA, computed by the different labelling approaches and different conditions, are reported in Figs. S11 and S12.

To better understand and visualize the performance of the in silico approaches and of the different structure prediction tools, informative correlation plots (Fig. S13) were obtained by extracting the mean and width of every distance distribution from the experimental and the in silico labelling data (Table S5). We investigated the dependence of the labelling approach (MtsslWizard Tight or Loose and MMM ambient or cryogenic temperature) on the four different nitroxide labels. Additionally, to get a numerical quantification of the curve discrepancies, the difference between the experimental and in silico mean values was also extracted (*Δ* mean) (Tables S8–S11).

Altogether, neither MMM nor MtsslWizard clearly outperformed the other. For the I6C/K28H/Q32H construct, independent of the prediction method (AlphaFold2, OmegaFold, ESMFold, and X-ray structure), MTSL was predicted best by MMM at ambient temperature, while all other nitroxides were best predicted by MtsslWizard Loose. On the other hand, the interpretation of the data for the I6H/N8H/K28C construct exhibiting bimodality in the experimental distance distributions was not as straightforward. Surprisingly, MTSL, that is widely employed in PDS experiments and whose behaviour might be expected to be well known, was the label on which MMM and MtsslWizard disagreed the most and neither could satisfyingly predict the experimental mean value. This may be a consequence of failing to predict the bimodal distance distribution obtained experimentally. However, the same problem was not observed for MPSL and IPSL; while both of them yielded bimodal distributions experimentally, which again were not satisfyingly predicted by either of the two labelling approaches, the predicted mean and width values were close to the experimental ones for both MMM and MtsslWizard.

Additional correlation plots (Fig. S14) were created to evaluate how the prediction tool affected the in silico labelling performances. In general, predicting the distance distributions between the CuNTA and the nitroxide on the β-sheet site seemed to be mostly unaffected by the choice of the structure prediction method (AlphaFold2, OmegaFold, and ESMFold), as expected for a small, well-known globular protein, like GB1. In contrast, the in silico spin labelling approach clearly had a greater impact on predicted distance distributions. One exception was the prediction for IDSL estimated by MMM both at ambient and cryogenic temperature for OmegaFold, which performed substantially worse than the other prediction tools and was the furthest away from the experimental values. However, the situation was found to be more complex for the I6H/N8H/K28C construct, where the choice of the model prediction influenced the outcome of the distance distribution more strongly, although always with a lower impact than the influence derived from the labelling choice. In general, MtsslWizard seems to be less affected by this trend than MMM.

To further assess the discrepancies between the experimental and the in silico data, we extracted the rmsd values between different distributions, to quantitatively investigate the similarity of the experimental and simulated distance distributions in terms of their overall shape (Tables S12–S15). These values mostly confirmed what was already discussed for the two modelling approaches and were highly consistent with the *Δ* mean values, where some values seemed to be in a better agreement with MMM, and others with MtsslWizard. Even if the bimodality was not captured by either of the two models, the increased broadness of the distributions was enough to reveal relatively low rmsd values even for the α-helix.

To comprehensively evaluate the in silico labelling methods, global rmsd values (Table S16) were extracted comparing all the mean values of the distance distributions of both GB1 constructs with the four nitroxide labels for a single labelling approach with the corresponding experimental values. In general, MtsslWizard with the Loose settings appeared to better represent the experimental data, while the other three approaches showed relatively small differences between each other. The influence of the structure prediction method was small. For MtsslWizard, the closest agreement was found using “Tight” settings based on the experimental structure, and this rmsd was significantly lower than for all other modelling approaches. Interestingly, the lowest rmsd values for MMM were obtained using the ESMFold model, whereas for both settings in MtsslWizard, the experimental structure yielded the best agreement. Very similar results could be observed for the distribution widths.

Although helices are often selected for SDSL, considering that they are well-studied secondary structural elements, in the case of GB1, it seemed to be a challenging site with respect to the sheet, giving rise to more ambiguous results in the distance distributions both from the experimental and in silico point of view. For the former, all labels, except for IDSL, showed some degree of bimodality. For the latter, we could get clues from the lower agreement between the different structure prediction methods and from the simulated distance distributions that were not able to predict the bimodality but only a higher broadness.

Overall, MMM and MtsslWizard rather complement than outperform each other. The introduced implementation for bipedal chelator ligands in the MtsslWizard now also allows predicting distance distributions between the copper and nitroxide spin labels similar to MMM. Therefore, we suggest that both approaches should be employed simultaneously to predict and compare distance distributions at a given labelling site. When the two labelling methods show significant disagreement, this could be interpreted as a warning and caution should be taken in the selection of that specific residue for experimental SDSL approaches, because the discrepancy could indicate propensity for conformational ambiguity in the PDS data.

## Conclusions

In summary, we presented a systematic investigation of two GB1 constructs with copper(II)-nitroxide orthogonal spin labelling, alternating both labels between the α-helix and the β-sheet. We confirmed that exploiting one rigid bipedal chelator agent such as CuNTA and a nitroxide radical provides a higher precision in the distance distributions with respect to the ones obtained with two nitroxide labels. This allowed an in-depth analysis of the behaviours of different nitroxide labels for the different sites, demonstrating how nitroxide labelling of the α-helix could introduce some ambiguity.

The flexibility of the label plays a key role regarding the width and shape of the resulting distance distribution. The chosen nitroxide should be selected carefully, considering also other properties. It is important to keep in mind that the width, mean, and shape of the distance distributions can originate from the properties of the spin label or conformational flexibility of the labelled system.

MPSL and IPSL showed the greatest differences between the α-helix and β-sheet sites; similar differences were less evident but still present for the MTSL label. In contrast, IDSL did not seem affected by the secondary structure of the labelling site, indicating how the length of the linker can affect width and shape of the distribution. It provided the narrowest and most unimodal distance distributions of all labels investigated. Therefore, we suggest that IDSL is a potentially undervalued and underused label that could possibly be exploited for gaining information on small conformational changes.

We analyzed the performance of the newly introduced bipedal labelling with copper(II) chelators for MtsslWizard on prediction of in silico distance distributions between orthogonal Cu^II^ and nitroxide labels. Overall, the results are comparable to the ones obtained from MMM, and with the help of the distribution mean, width, and rmsd values, we demonstrated how the in silico labelling approaches displayed generally good but not perfect agreement in prediction of the experimental data. However, disagreement between the two methods could be an indication of the presence of an ambiguous labelling site with, for example, a bimodal conformer distribution.

Frequently, when selecting cysteine mutants for SDSL, α-helices are chosen as well-defined structural elements. Interestingly, here, we found that this site led to more ambiguity in the interpretation of the distance distributions than the β-sheet site. Further research is required to establish whether these observations for the different secondary structures can be generalized.

The research data underpinning this publication will be accessible at https://doi.org/10.17630/71f8e2e5-9f57-4160-8c32-de1b37d4c073 [65].

### Supplementary Information

Below is the link to the electronic supplementary material.

Supplementary file 1 (PDF 12755 KB)

Supplementary file 2 (PSE 18 KB)

## Data Availability

The research data underpinning this publication will be accessible at https://doi.org/10.17630/71f8e2e5-9f57-4160-8c32-de1b37d4c073 [65].
