# Supplementary material for: Spectroscopically Orthogonal Labelling to Disentangle Site-Specific Nitroxide Label Distributions
Source: Appl Magn Reson. 2023 Sep 24;55(1-3):187–205. doi: 10.1007/s00723-023-01611-1 (PMC10861635; doi:10.1007/s00723-023-01611-1)
Supplement: Supplementary file 1 — (PDF 12755 KB) [file 723_2023_1611_MOESM1_ESM.pdf]

Supplementary Information

Spectroscopically orthogonal labelling to disentangle site-specific nitroxide label distributions

Valentina Vitali[1,2], Katrin Ackermann[1], Gregor Hagelueken*[3], and Bela E. Bode*[1]

[1]EaStCHEM School of Chemistry, Biomedical Sciences Research Complex, and Centre of Magnetic Resonance, University of St Andrews, North Haugh, St Andrews, KY16 9ST, Scotland; [2] Magnetic Resonance Center (CERM), University of Florence, via Luigi Sacconi 6, Sesto Fiorentino 50019, Italy; Department of Chemistry "Ugo Schiff", University of Florence, via della Lastruccia 3, Sesto Fiorentino 50019, Italy; [3]Institute of Structural Biology, Biomedical Center, University of Bonn, Venusberg-Campus 1, 53127 Bonn, Germany

Table of contents

I Results and Discussion ***1***

1.1 Mass Spectrometry **1**

1.2 Continuous Wave EPR Spectroscopy **6**

1.3 RIDME raw spectra **7**

1.4 RIDME processed spectra and distance distributions **12**

1.5 Sensitivity data for vtRIDME and ctRIDME **20**

1.6 Distance distributions extracted from vtRIDME and ctRIDME with and without deconvolution **21**

1.7 Mean, Width and Full Width at Half Maximum for ctRIDME and vtRIDME both with and without deconvolution **23**

1.8 Comparison of the distance distributions between two nitroxides labels or between the copper(II) and the nitroxides **26**

1.9 Modelling with MMM and MtsslWizard **28**

1.10 Correlation plots and mean, widths values of the modelled distributions **33**

1.11 Root mean square deviation (RMSD) values **43**

II References ***46***

I Results and Discussion

1.1 Mass Spectrometry

Acquired ESI mass spectrometry spectra show only a single peak at the expected mass of the protein (unlabelled or labelled with the four nitroxides, respectively). The only exception is for the 6C MTSL sample where a peak with smaller intensity belonging to the unreacted protein can also be detected (**Fig. S1**). For the GB1 I6H/N8H/K28C control construct, a smaller peak corresponding to the dimer (12487 Da) can be observed. However, no dimer peak was detected in any of the labelled samples, leading us to assume that its formation may have occurred during the time frame between removing the DTT and performing the mass spectrometry measurement.
The expected masses and the actual experimental masses for the different samples are reported in **Table S1**.

ESI-MS

Mass values (Da)	Control		MTSL		MPSL		IPSL		IDSL	
	6C	28C	6C	28C	6C	28C	6C	28C	6C	28C
**Expected**	6244.8	6230.8	6414.8	6428.8	6467.8	6481.8	6427.8	6441.8	6401.8	6415.8
**Obtained**	6244.0	6230.6	6414.8	6429.0	6467.9	6481.0	6427.7	6441.1	6401.8	6415.1

Table S1 Expected and obtained mass values (Da) for both GB1 I6C/K28H/Q32H and I6H/N8H/K28C GB1 constructs with and without (control) the four different nitroxide labels MTSL, MPSL, IPSL and IDSL

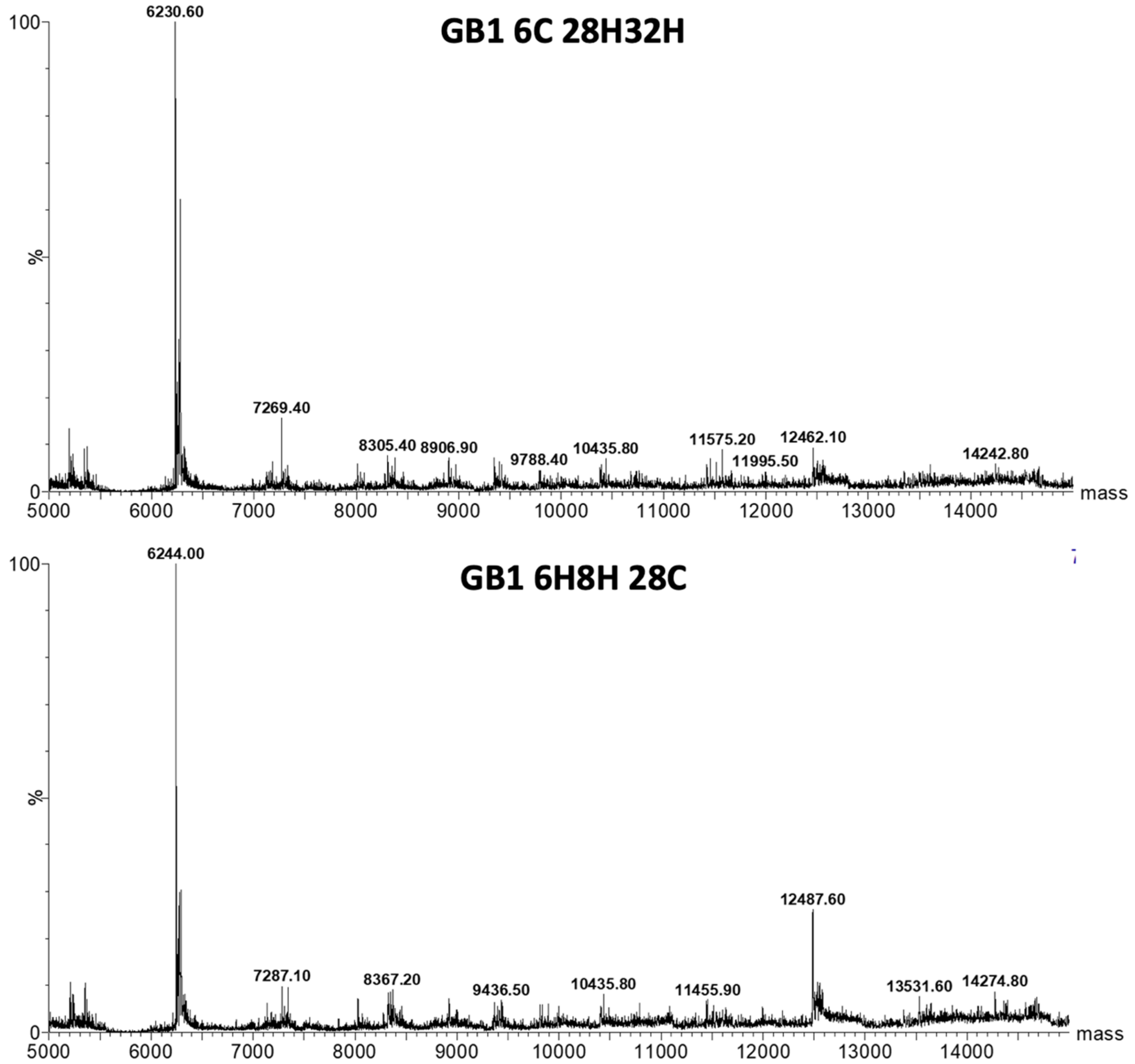

Fig. S1 ESI-MS spectra of the two GB1 constructs I6C/K28H/Q32H and I6H/N8H/K28C with and without (control) the four nitroxide labels MTSL, MPSL, IPSL and IDSL

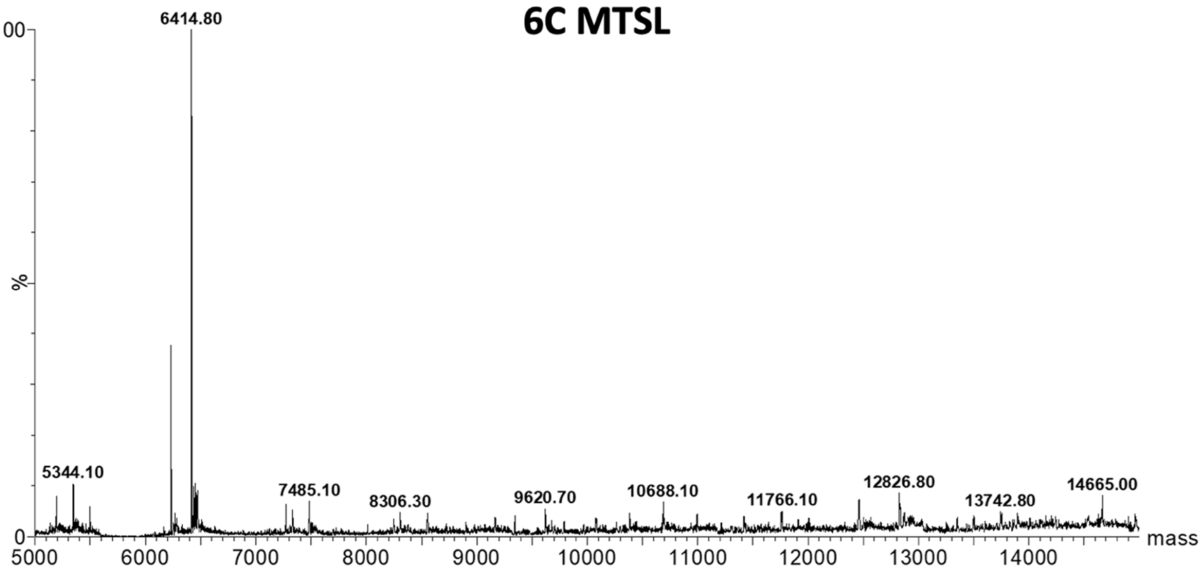

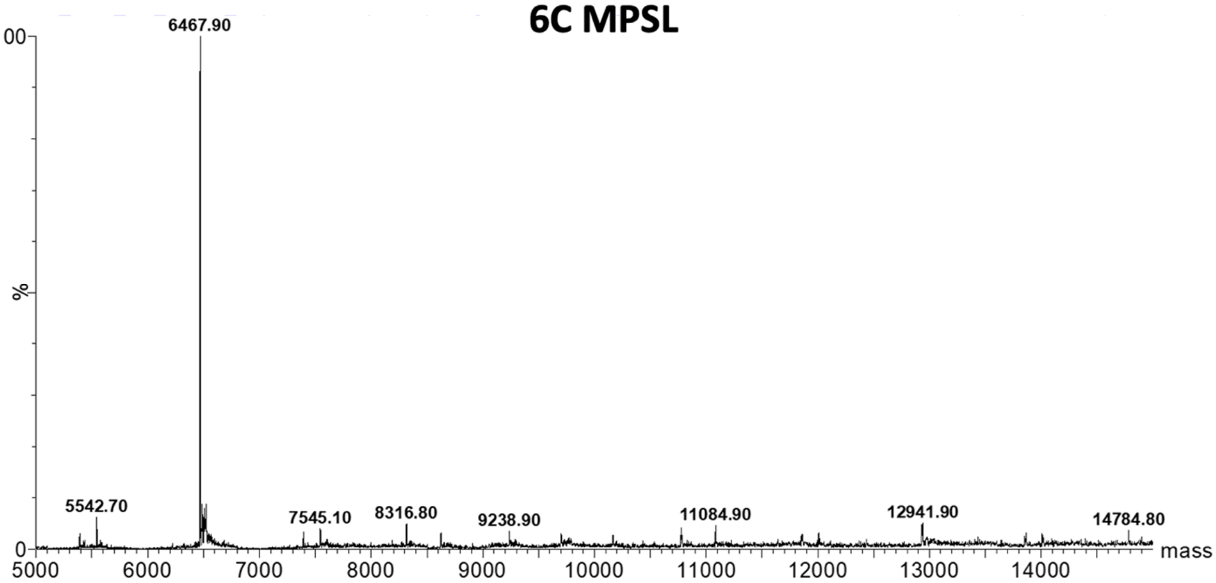

Fig. S1, continued ESI-MS spectra of the two GB1 constructs I6C/K28H/Q32H and I6H/N8H/K28C with and without (control) the four nitroxide labels MTSL, MPSL, IPSL and IDSL

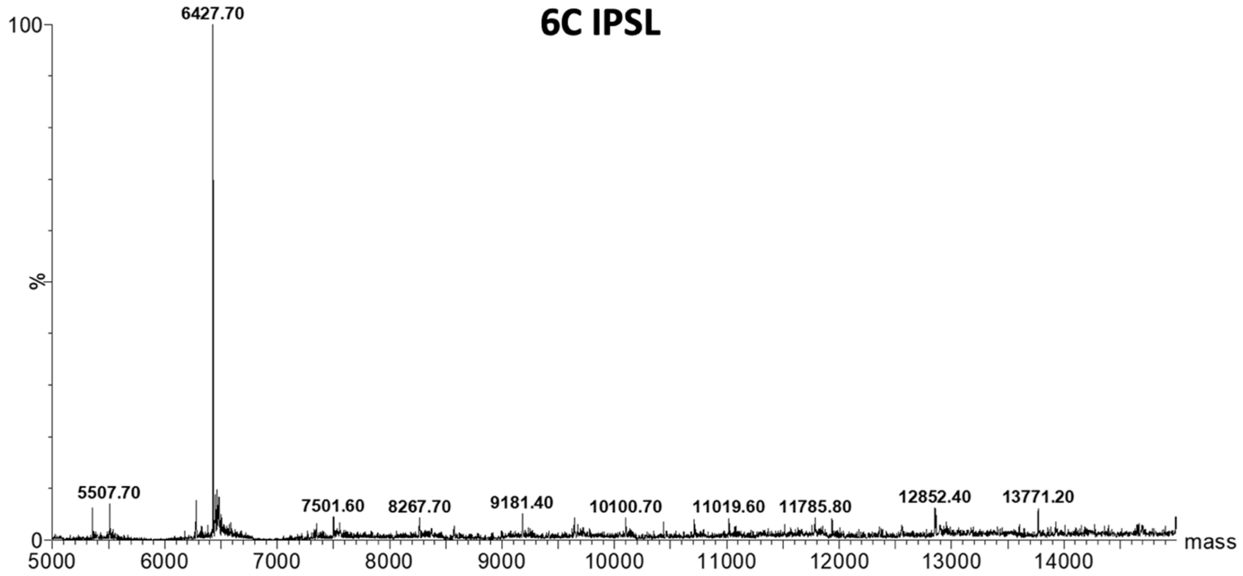

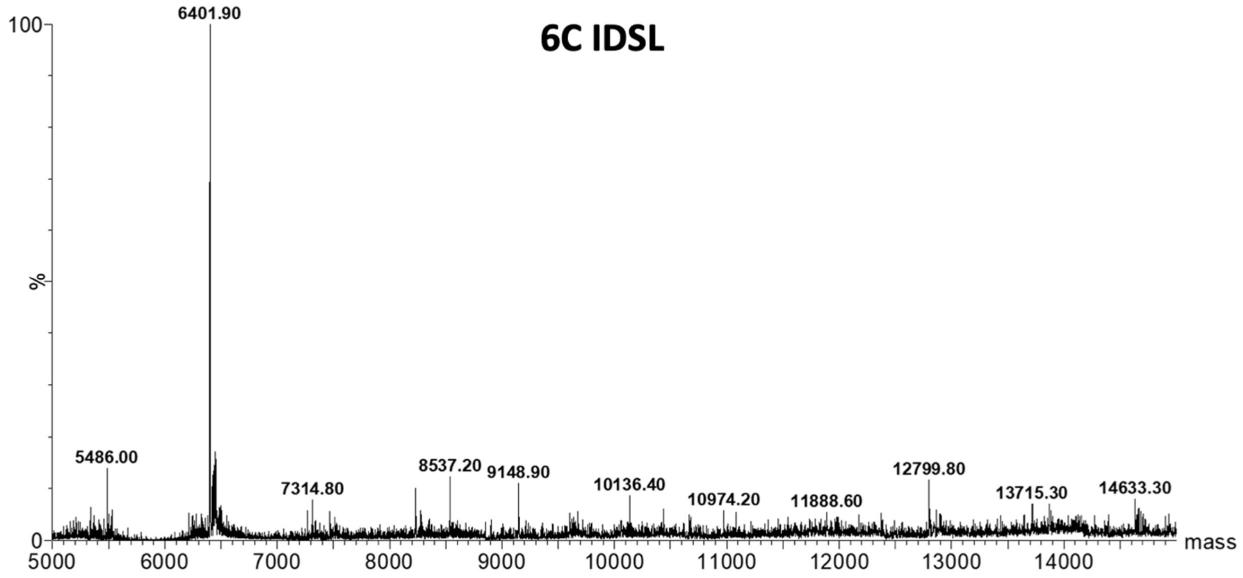

Fig. S1, continued ESI-MS spectra of the two GB1 constructs I6C/K28H/Q32H and I6H/N8H/K28C with and without (control) the four nitroxide labels MTSL, MPSL, IPSL and IDSL

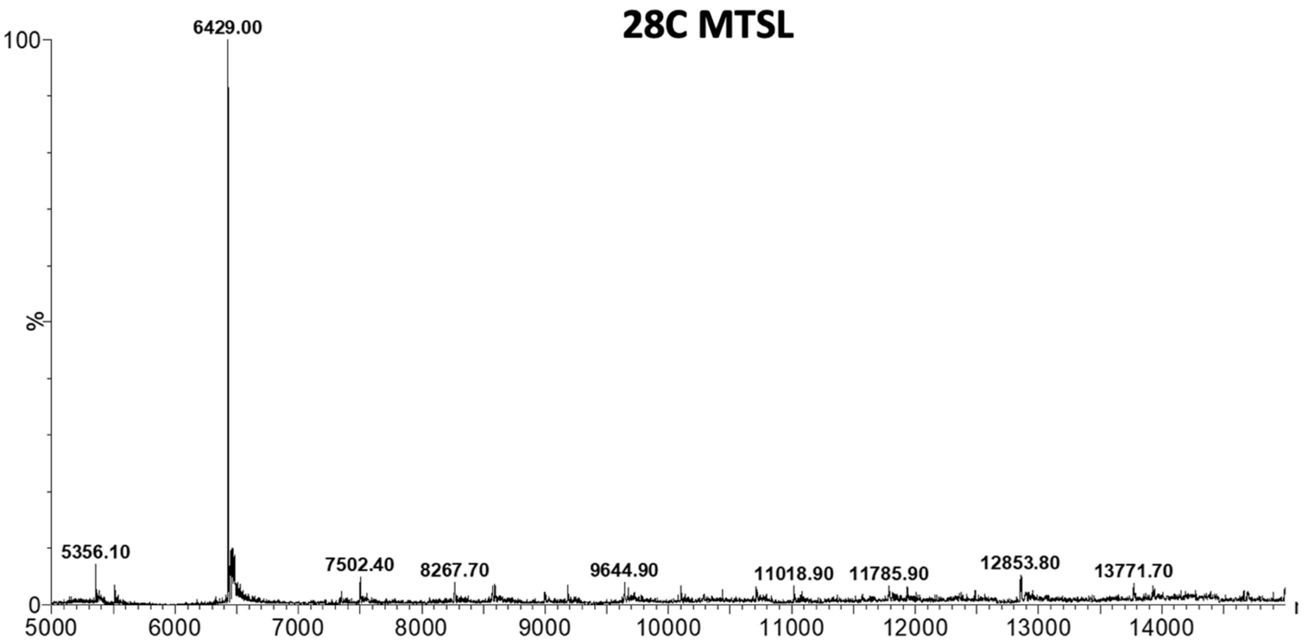

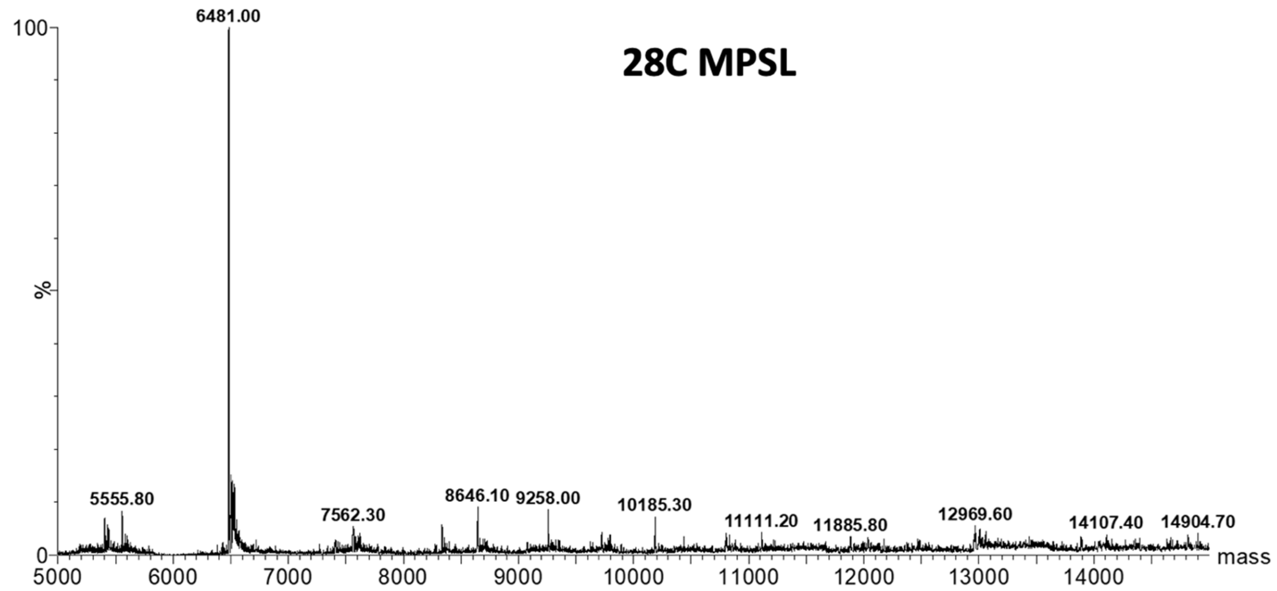

Fig. S1, continued ESI-MS spectra of the two GB1 constructs I6C/K28H/Q32H and I6H/N8H/K28C with and without (control) the four nitroxide labels MTSL, MPSL, IPSL and IDSL

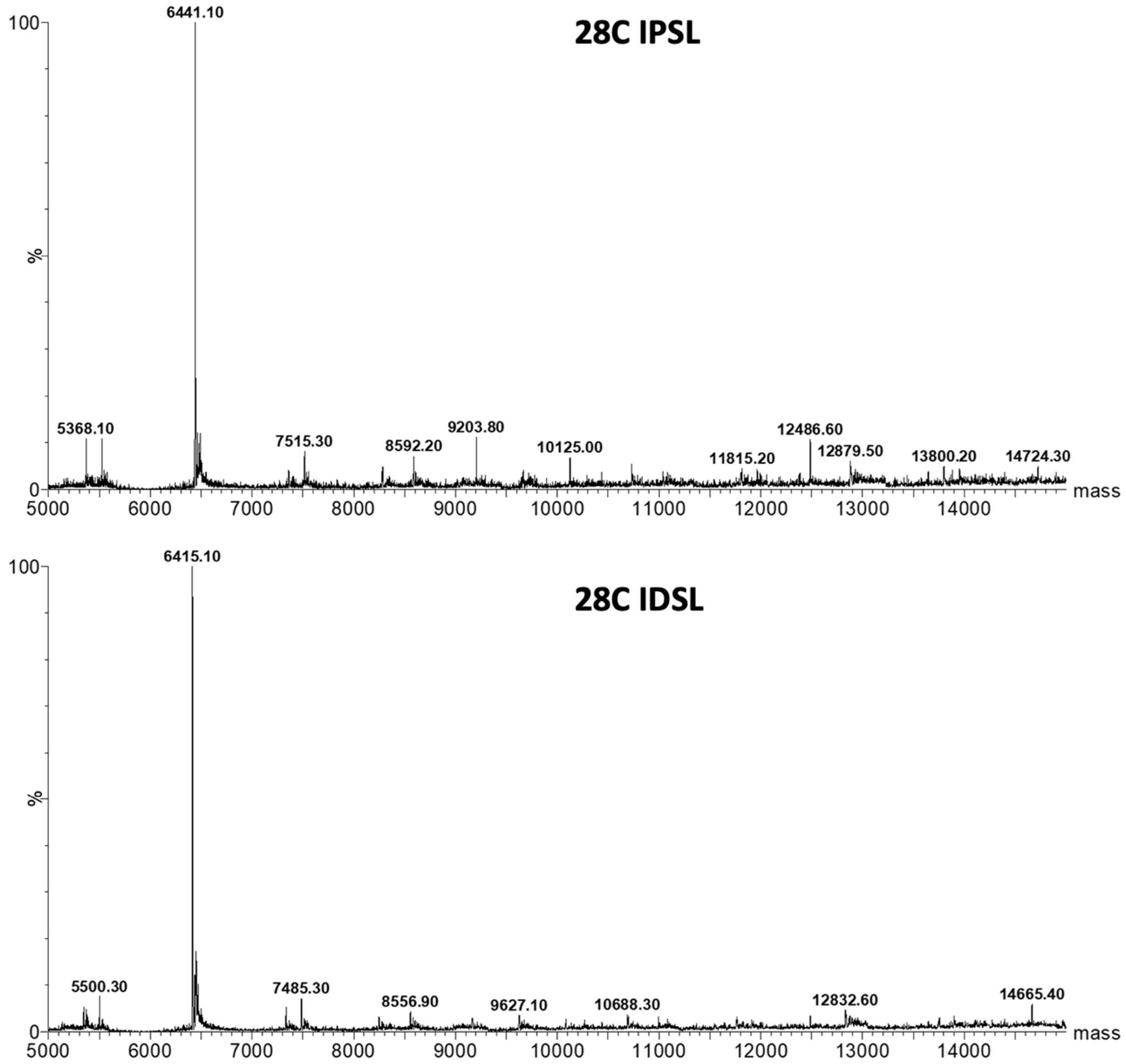

Fig. S1, continued ESI-MS spectra of the two GB1 constructs I6C/K28H/Q32H and I6H/N8H/K28C with and without (control) the four nitroxide labels MTSL, MPSL, IPSL and IDSL

1.2 Continuous Wave EPR Spectroscopy

Individual CW EPR spectra and corresponding labelling efficiencies are given in **Fig. S2** and **Table S2**. Quantitative labelling was obtained for all but the MTSL labelling of the I6C/K28H/Q32H construct, where the labelling efficiency was found to be lower, consistent with what was observed from the ESI-MS spectra.

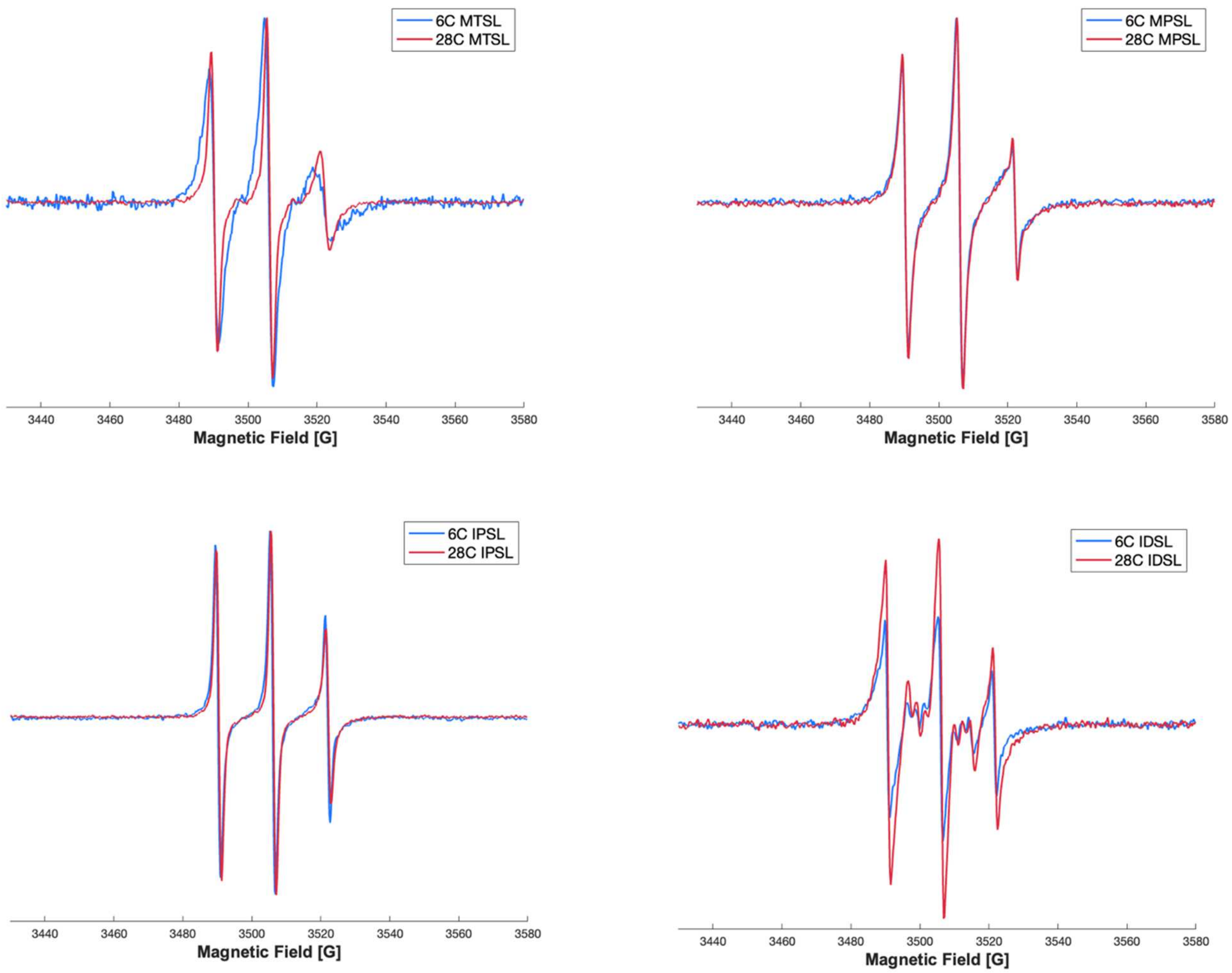

Fig. S2 CW-EPR plots of the GB1 I6C/K28H/Q32H (in blue) and I6H/N8H/K28C (in red) GB1 constructs with the four distinct nitroxide labels, MTSL, MPSL, IPSL and IDSL

Labelling Efficiency

**CW-EPR**	**MTSL**		**MPSL**		**IPSL**		**IDSL**	
	6C	28C	6C	28C	6C	28C	6C	28C
	87%	98%	115%	99%	105%	100%	114%	127%

Table S2 Labelling efficiencies for both GB1 I6C/K28H/Q32H and I6H/N8H/K28C GB1 constructs with the four nitroxides labels, MTSL, MPSL, IPSL and IDSL

1.3 RIDME raw spectra

RIDME raw spectra for both the variable and constant time RIDME, their reference background traces and the superimposed traces after deconvolution are reported in **Fig. S3.**

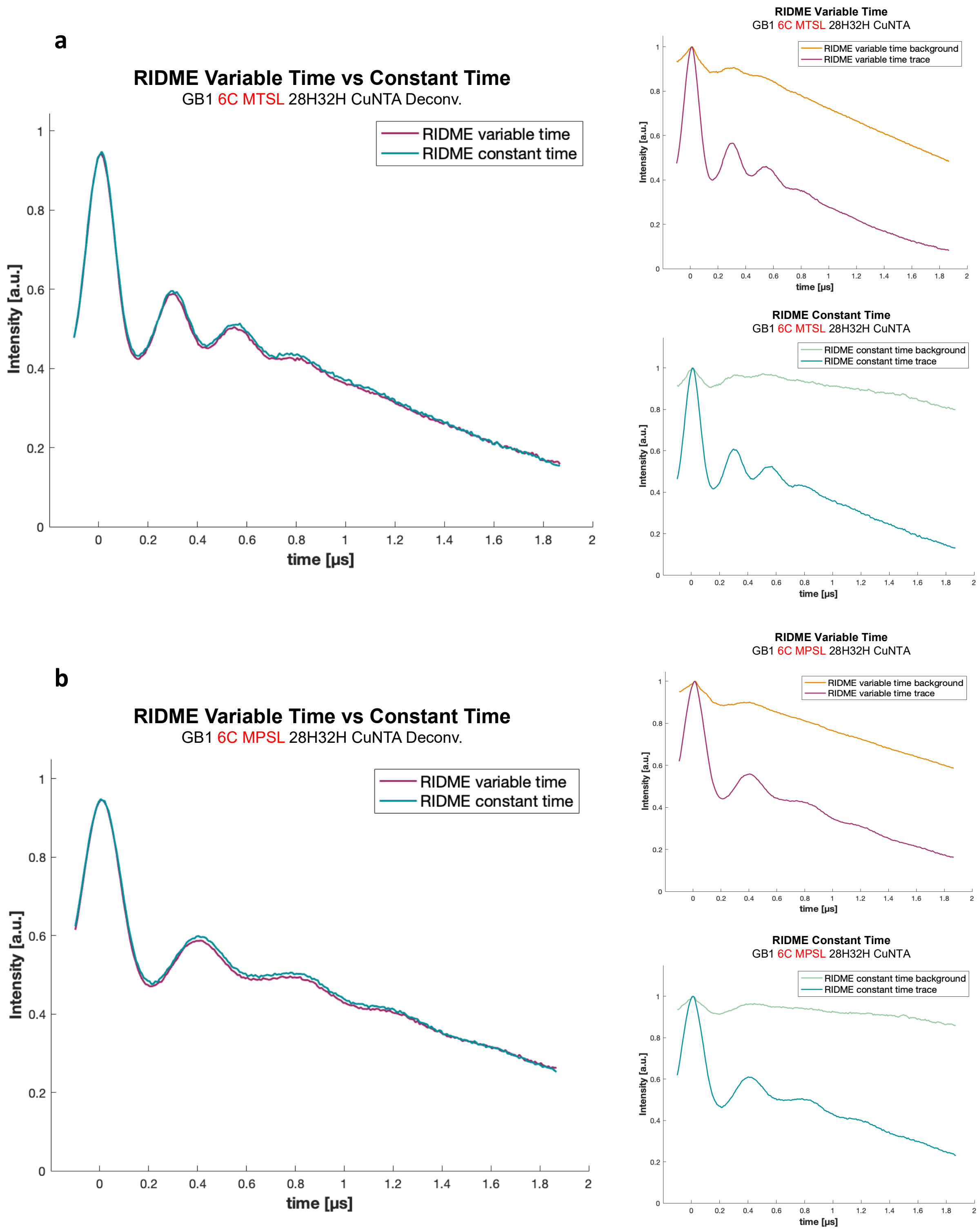

Fig. S3 Right: ctRIDME (cyan) and vtRIDME (magenta) raw data traces and their respective background traces (light green and orange). Left: superimposed constant time and variable time RIDME traces after deconvolution **a)** GB1 I6C/K28H/Q32H MTSL Cu-NTA, **b)** GB1 I6C/K28H/Q32H MPSL CuNTA, **c)** GB1 I6C/K28H/Q32H IPSL CuNTA, **d)** GB1 I6C/K28H/Q32H IDSL CuNTA, **e)** GB1 I6H/N8H/K28C CuNTA MTSL, **f)** GB1 I6H/N8H/K28C CuNTA MPSL, **g)** GB1 I6H/N8H/K28C CuNTA IPSL, **h)** GB1 I6H/N8H/K28C CuNTA IDSL

c

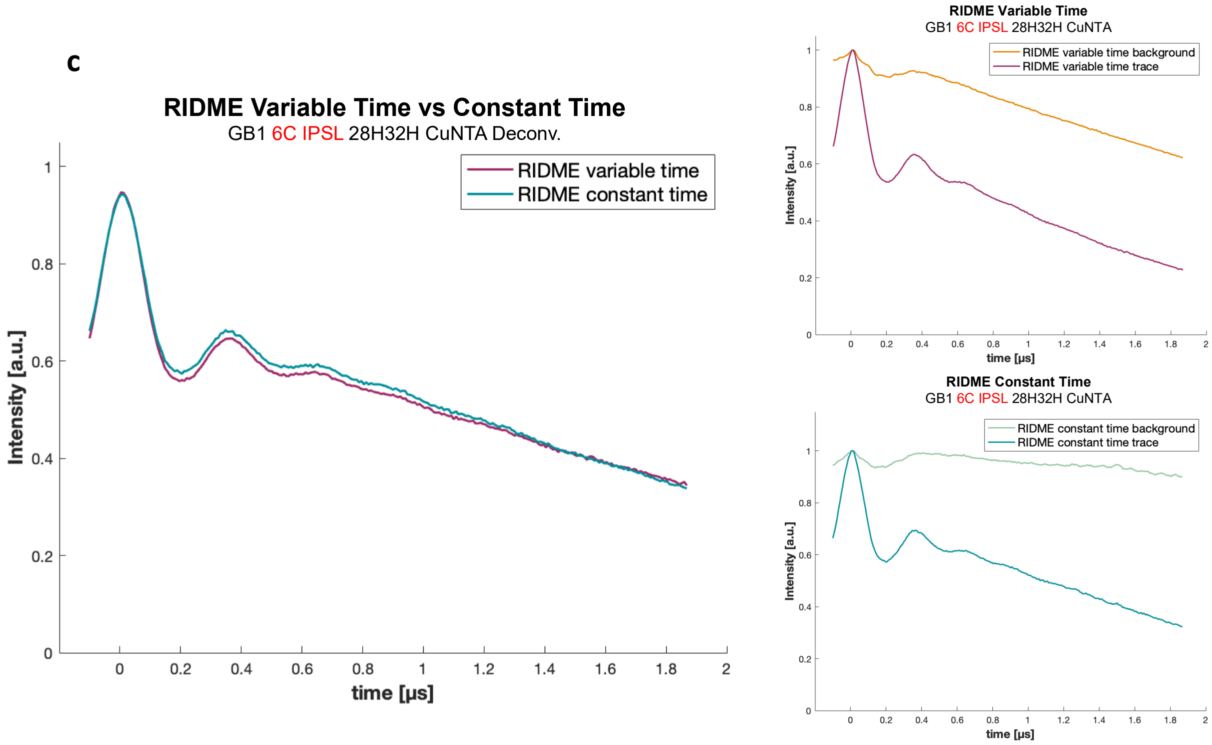

d

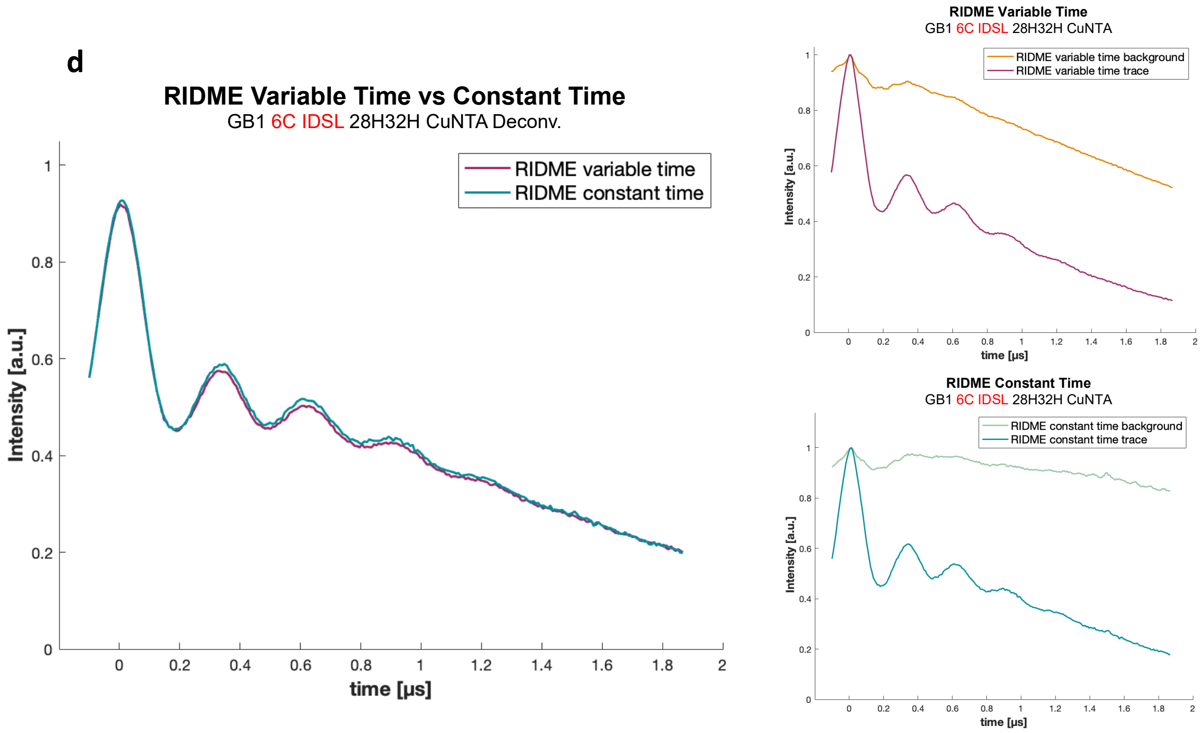

Fig. S3, continued Right: ctRIDME (cyan) and vtRIDME (magenta) raw data traces and their respective background traces (light green and orange). Left: superimposed constant time and variable time RIDME traces after deconvolution **a)** GB1 I6C/K28H/Q32H MTSL CuNTA, **b)** GB1 I6C/K28H/Q32H MPSL CuNTA, **c)** GB1 I6C/K28H/Q32H IPSL CuNTA, **d)** GB1 I6C/K28H/Q32H IDSL CuNTA, **e)** GB1 I6H/N8H/K28C CuNTA MTSL, **f)** GB1 I6H/N8H/K28C CuNTA MPSL, **g)** GB1 I6H/N8H/K28C CuNTA IPSL, **h)** GB1 I6H/N8H/K28C CuNTA IDSL

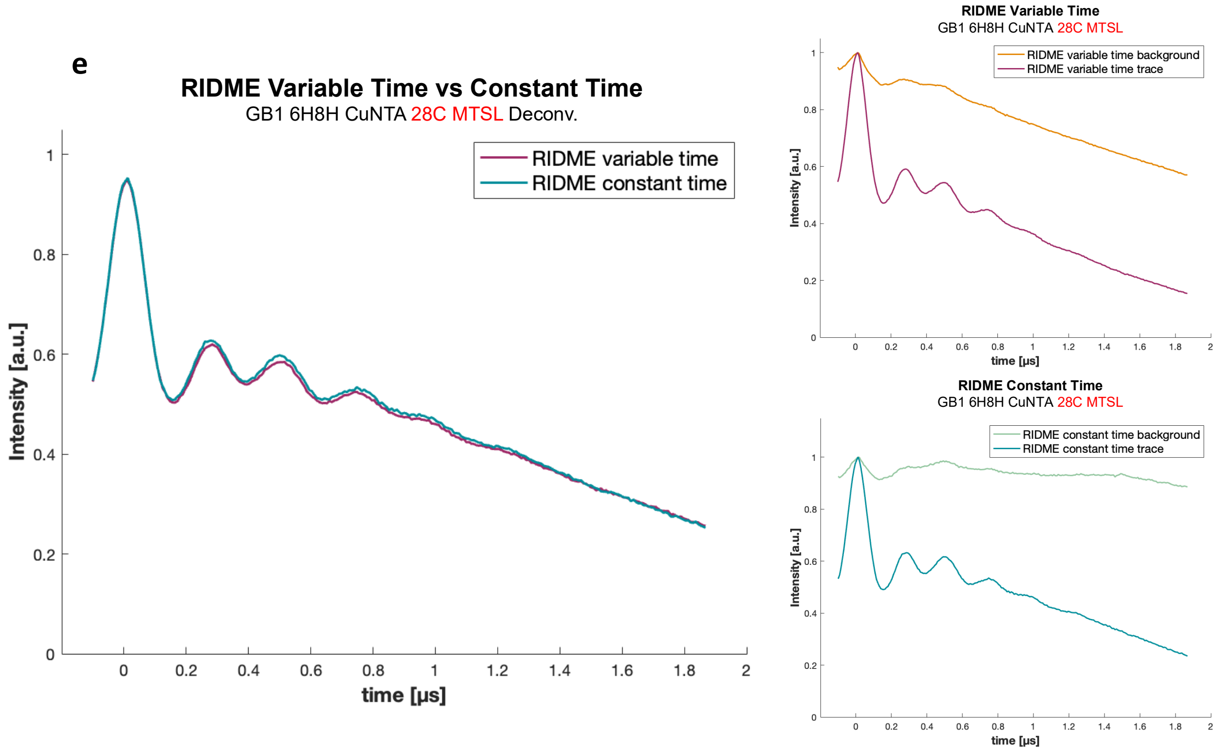

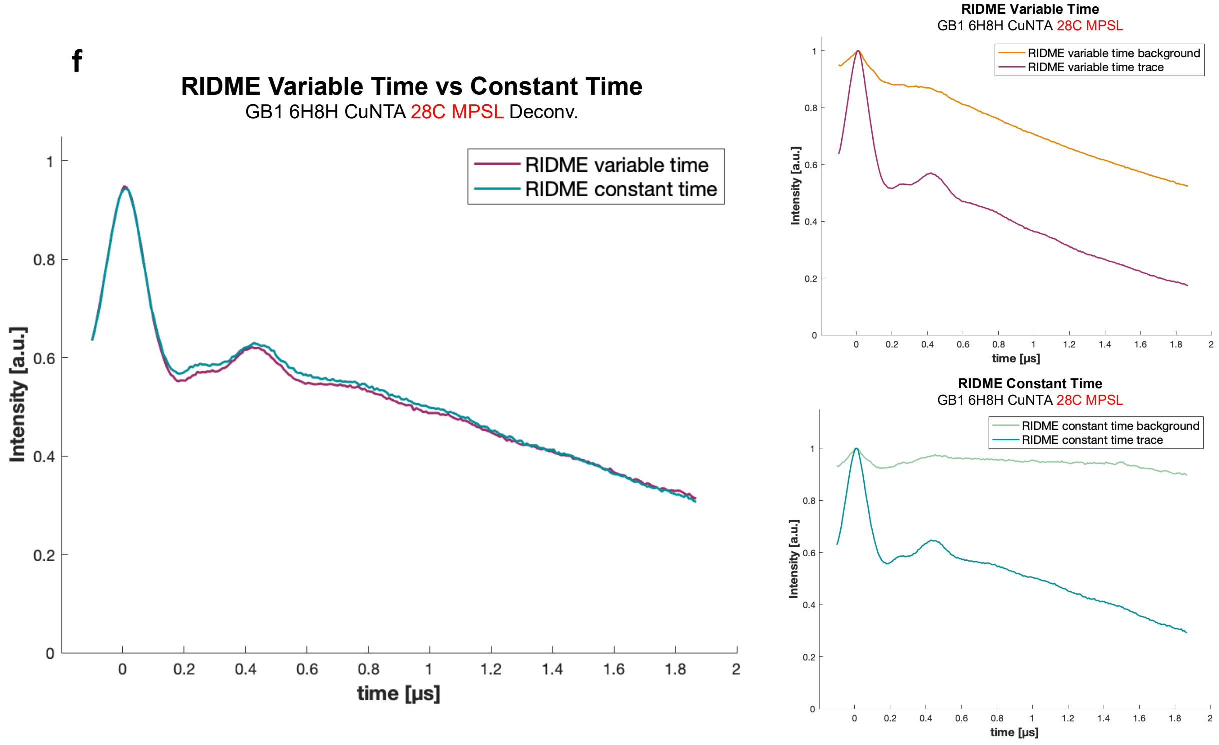

Fig. S3, continued Right: ctRIDME (cyan) and vtRIDME (magenta) raw data traces and their respective background traces (light green and orange). Left: superimposed constant time and variable time RIDME traces after deconvolution **a)** GB1 I6C/K28H/Q32H MTSL CuNTA, **b)** GB1 I6C/K28H/Q32H MPSL CuNTA, **c)** GB1 I6C/K28H/Q32H IPSL CuNTA, **d)** GB1 I6C/K28H/Q32H IDSL CuNTA, **e)** GB1 I6H/N8H/K28C CuNTA MTSL, **f)** GB1 I6H/N8H/K28C CuNTA MPSL, **g)** GB1 I6H/N8H/K28C CuNTA IPSL, **h)** GB1 I6H/N8H/K28C CuNTA IDSL

g

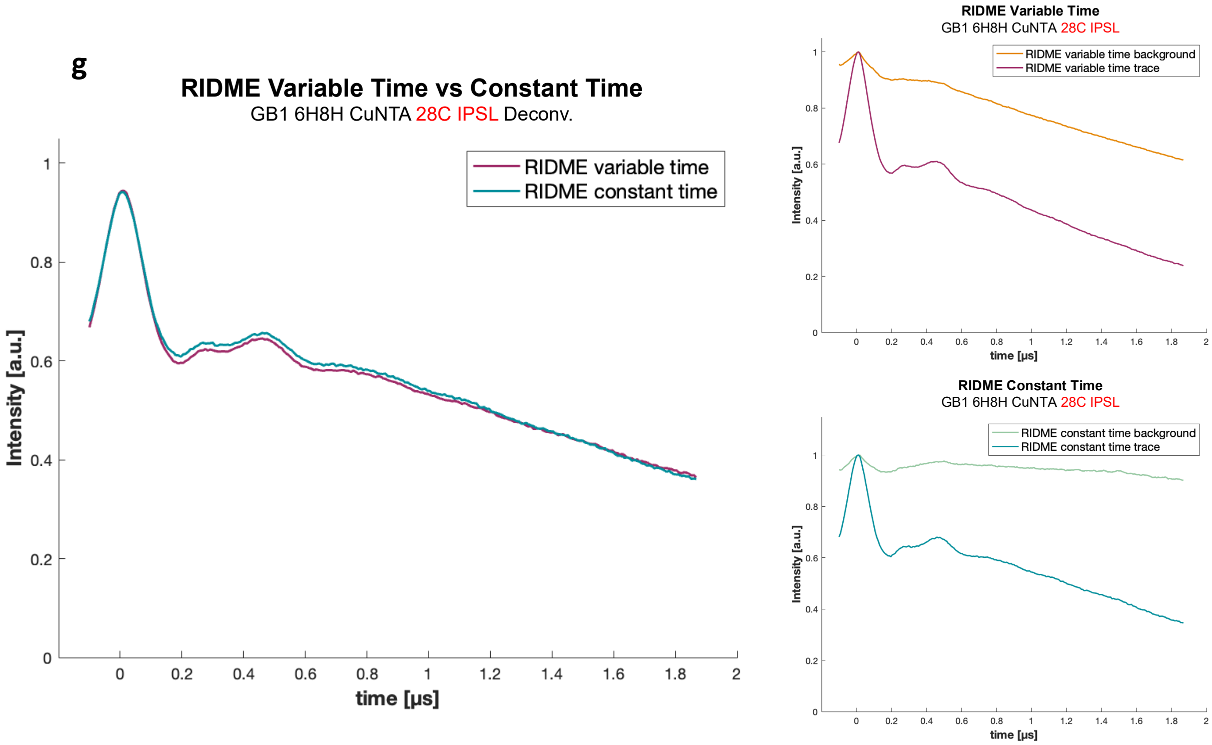

h

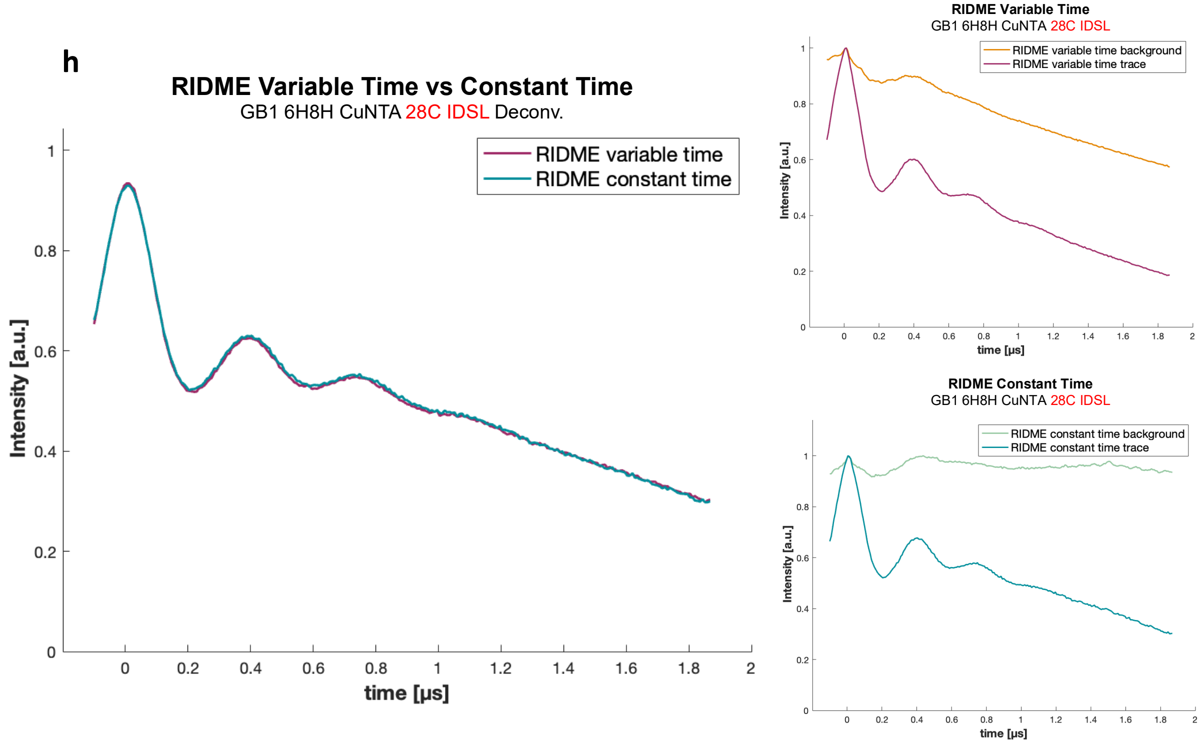

Fig. S3, continued Right: ctRIDME (cyan) and vtRIDME (magenta) raw data traces and their respective background traces (light green and orange). Left: superimposed constant time and variable time RIDME traces after deconvolution **a)** GB1 I6C/K28H/Q32H MTSL CuNTA, **b)** GB1 I6C/K28H/Q32H MPSL CuNTA, **c)** GB1 I6C/K28H/Q32H IPSL CuNTA, **d)** GB1 I6C/K28H/Q32H IDSL CuNTA, **e)** GB1 I6H/N8H/K28C CuNTA MTSL, **f)** GB1 I6H/N8H/K28C CuNTA MPSL, **g)** GB1 I6H/N8H/K28C CuNTA IPSL, **h)** GB1 I6H/N8H/K28C CuNTA IDSL

1.4 RIDME processed spectra and distance distributions

Raw and background-corrected RIDME traces, processed with Deer Analysis, and their corresponding distance distributions are reported in **Fig. S4**.

a

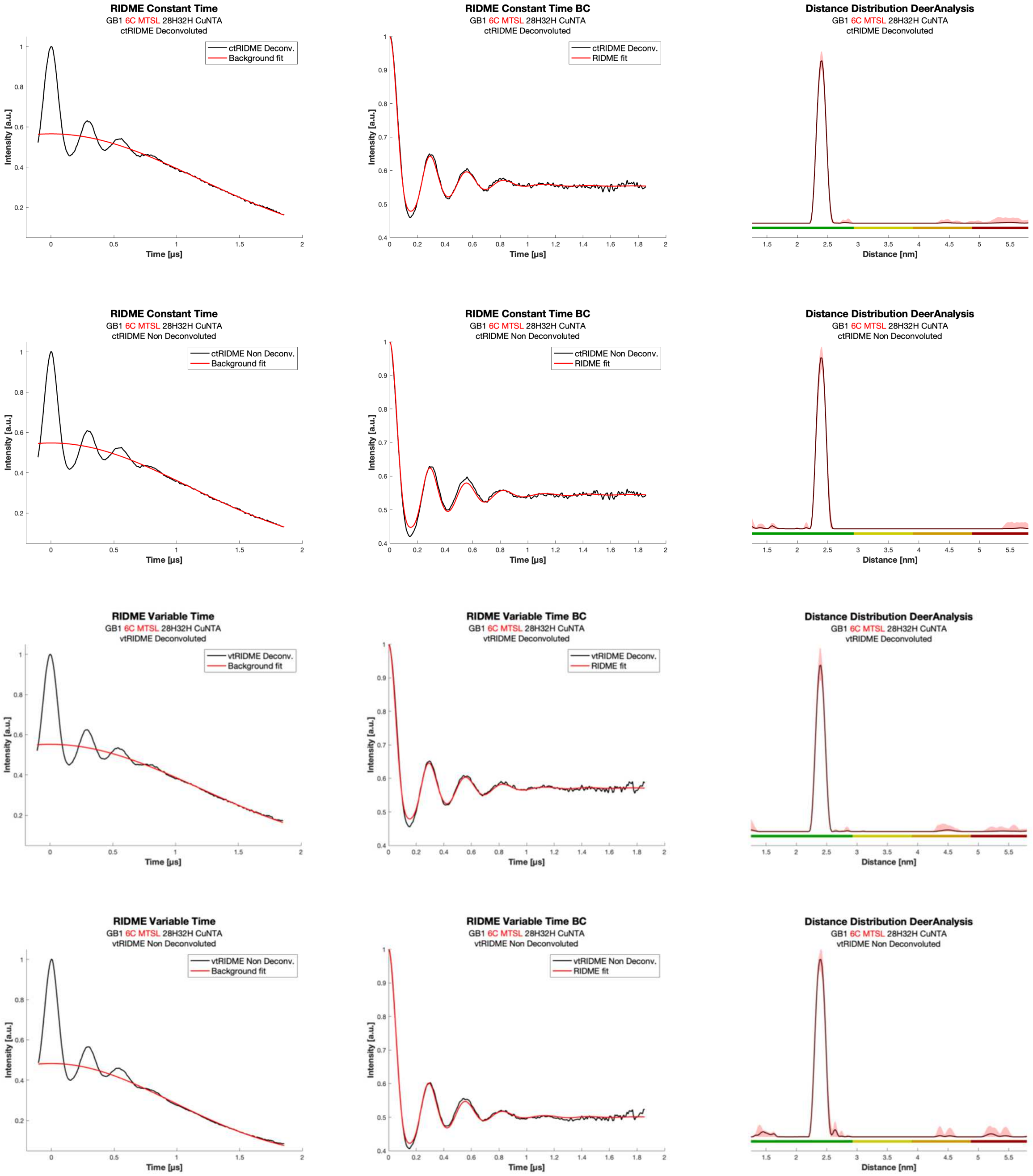

Fig. S4 Left: raw RIDME traces (black) with background function (red); middle: background-corrected (BC) data (black) with fit (red); right: corresponding distance distributions given as 95% confidence intervals (± 2σ) with 50% noise added for error estimation during statistical analysis. Colour bars represent reliability ranges (green: shape reliable; yellow: mean and width reliable; orange: mean reliable: red: no quantification possible). First row: ctRIDME deconvoluted, second row: ctRIDME non deconvoluted, third row: vtRIDME deconvoluted, fourth row: vtRIDME non deconvoluted. **a)** GB1 I6C/K28H/Q32H MTSL CuNTA, **b)** GB1 I6C/K28H/Q32H MPSL CuNTA, **c)** GB1 I6C/K28H/Q32H IPSL CuNTA, **d)** GB1 I6C/K28H/Q32H IDSL CuNTA, **e)** GB1 I6H/N8H/K28C CuNTA MTSL, **f)** GB1 I6H/N8H/K28C CuNTA MPSL, **g)** GB1 I6H/N8H/K28C CuNTA IPSL, **h)** GB1 I6H/N8H/K28C CuNTA IDSL

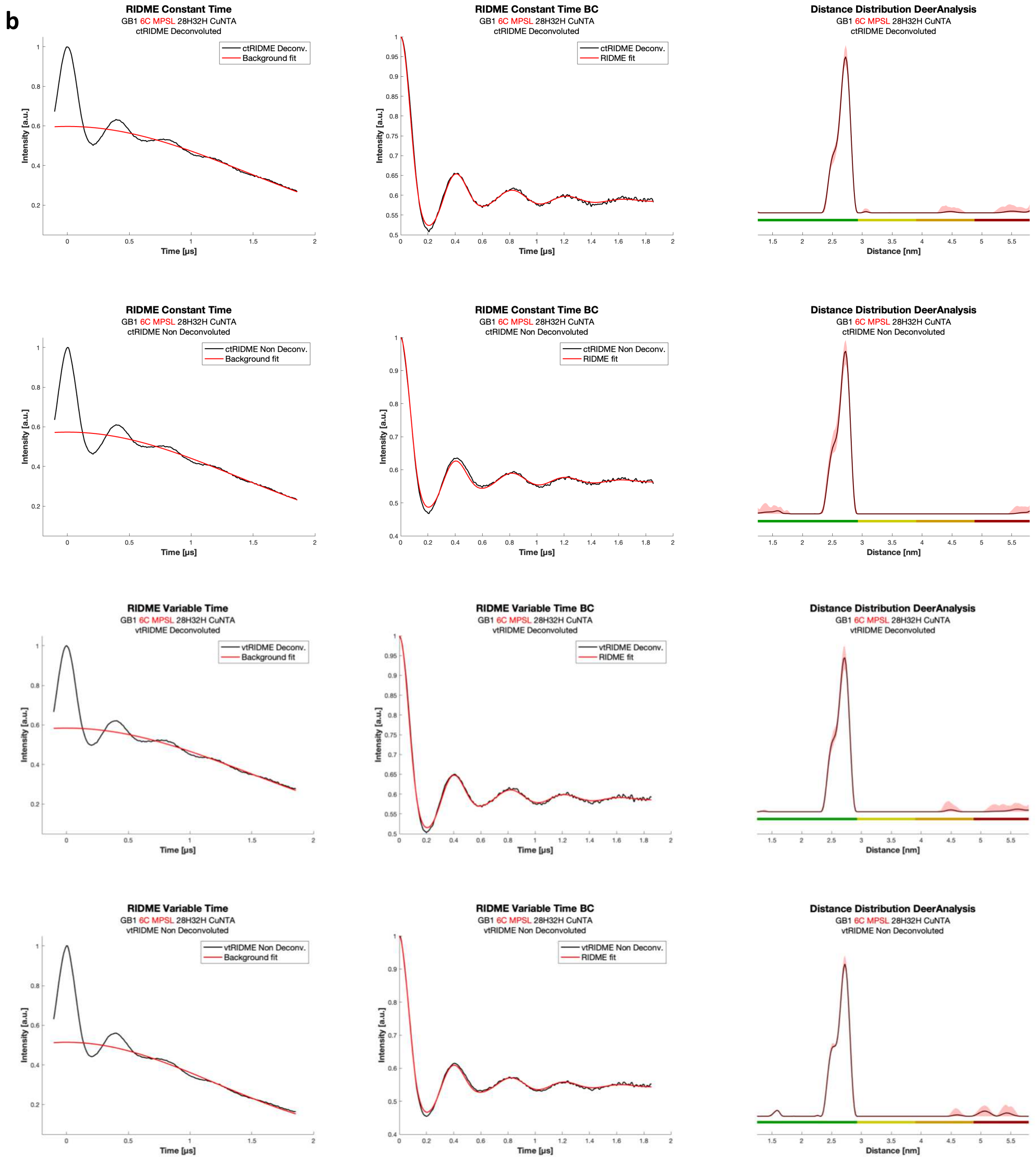

Fig. S4, continued Left: raw RIDME traces (black) with background function (red); middle: background-corrected (BC) data (black) with fit (red); right: corresponding distance distributions given as 95% confidence intervals (± 2σ) with 50% noise added for error estimation during statistical analysis. Colour bars represent reliability ranges (green: shape reliable; yellow: mean and width reliable; orange: mean reliable: red: no quantification possible). First row: ctRIDME deconvoluted, second row: ctRIDME non deconvoluted, third row: vtRIDME deconvoluted, fourth row: vtRIDME non deconvoluted **a)** GB1 I6C/K28H/Q32H MTSL CuNTA, **b)** GB1 I6C/K28H/Q32H MPSL CuNTA, **c)** GB1 I6C/K28H/Q32H IPSL CuNTA, **d)** GB1 I6C/K28H/Q32H IDSL CuNTA, **e)** GB1 I6H/N8H/K28C CuNTA MTSL, **f)** GB1 I6H/N8H/K28C CuNTA MPSL, **g)** GB1 I6H/N8H/K28C CuNTA IPSL, **h)** GB1 I6H/N8H/K28C CuNTA IDSL

c

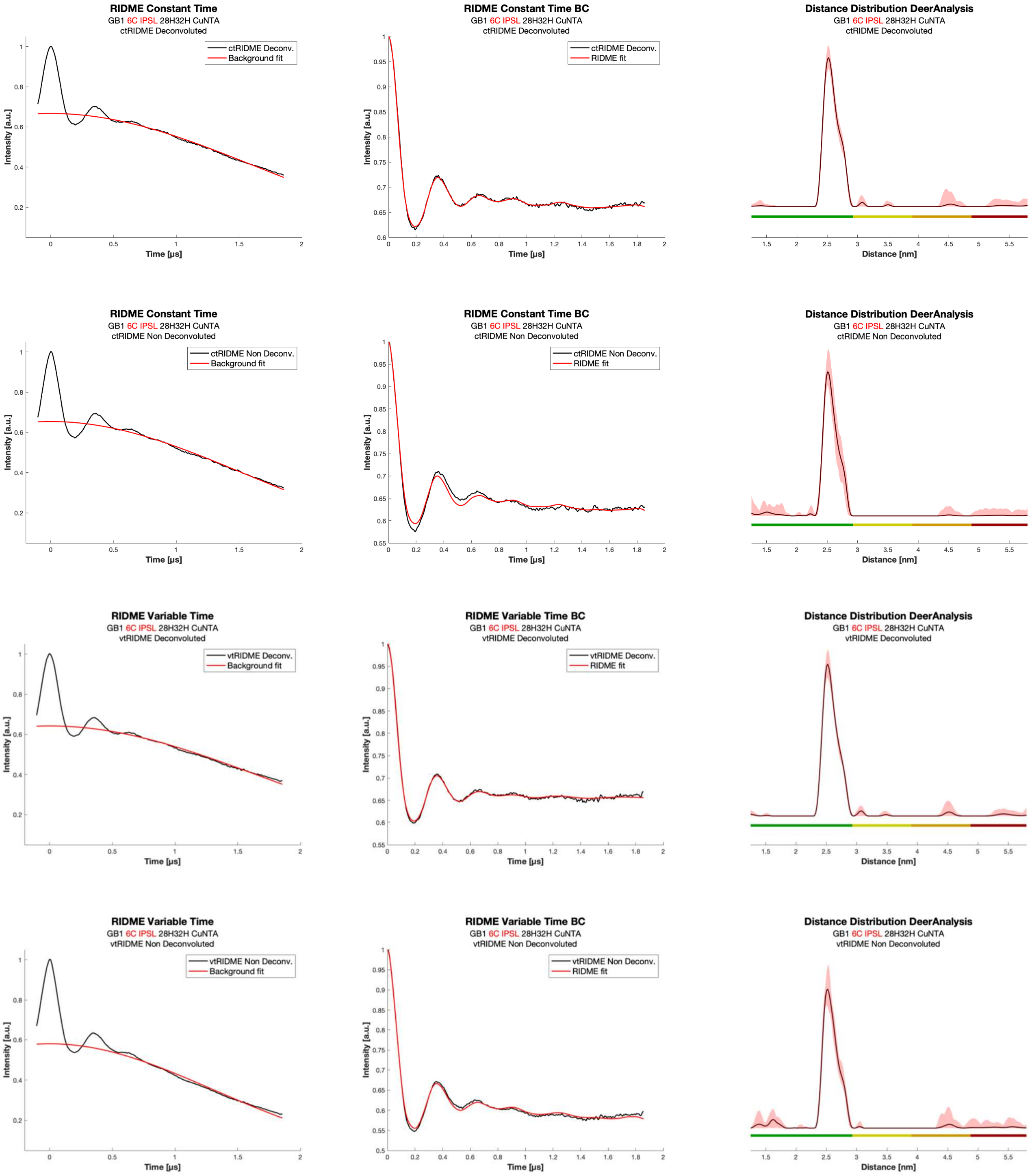

Fig. S4, continued Left: raw RIDME traces (black) with background function (red); middle: background-corrected (BC) data (black) with fit (red); right: corresponding distance distributions given as 95% confidence intervals (± 2σ) with 50% noise added for error estimation during statistical analysis. Colour bars represent reliability ranges (green: shape reliable; yellow: mean and width reliable; orange: mean reliable: red: no quantification possible). First row: ctRIDME deconvoluted, second row: ctRIDME non deconvoluted, third row: vtRIDME deconvoluted, fourth row: vtRIDME non deconvoluted **a)** GB1 I6C/K28H/Q32H MTSL CuNTA, **b)** GB1 I6C/K28H/Q32H MPSL CuNTA, **c)** GB1 I6C/K28H/Q32H IPSL CuNTA, **d)** GB1 I6C/K28H/Q32H IDSL CuNTA, **e)** GB1 I6H/N8H/K28C CuNTA MTSL, **f)** GB1 I6H/N8H/K28C CuNTA MPSL, **g)** GB1 I6H/N8H/K28C CuNTA IPSL, **h)** GB1 I6H/N8H/K28C CuNTA IDSL

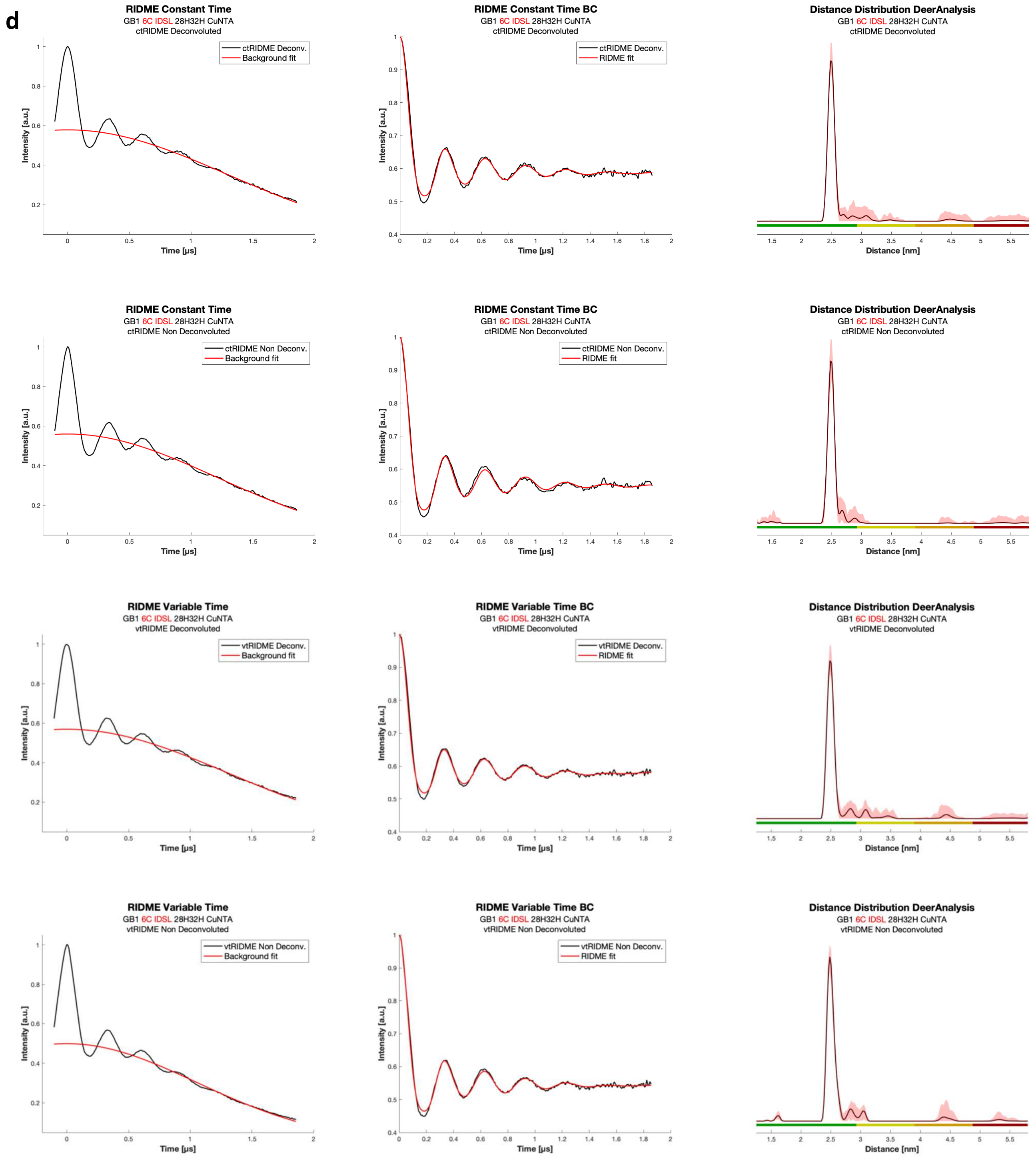

Fig. S4, continued Left: raw RIDME traces (black) with background function (red); middle: background-corrected (BC) data (black) with fit (red); right: corresponding distance distributions given as 95% confidence intervals (± 2σ) with 50% noise added for error estimation during statistical analysis. Colour bars represent reliability ranges (green: shape reliable; yellow: mean and width reliable; orange: mean reliable: red: no quantification possible). First row: ctRIDME deconvoluted, second row: ctRIDME non deconvoluted, third row: vtRIDME deconvoluted, fourth row: vtRIDME non deconvoluted **a)** GB1 I6C/K28H/Q32H MTSL CuNTA, **b)** GB1 I6C/K28H/Q32H MPSL CuNTA, **c)** GB1 I6C/K28H/Q32H IPSL CuNTA, **d)** GB1 I6C/K28H/Q32H IDSL CuNTA, **e)** GB1 I6H/N8H/K28C CuNTA MTSL, **f)** GB1 I6H/N8H/K28C CuNTA MPSL, **g)** GB1 I6H/N8H/K28C CuNTA IPSL, **h)** GB1 I6H/N8H/K28C CuNTA IDSL

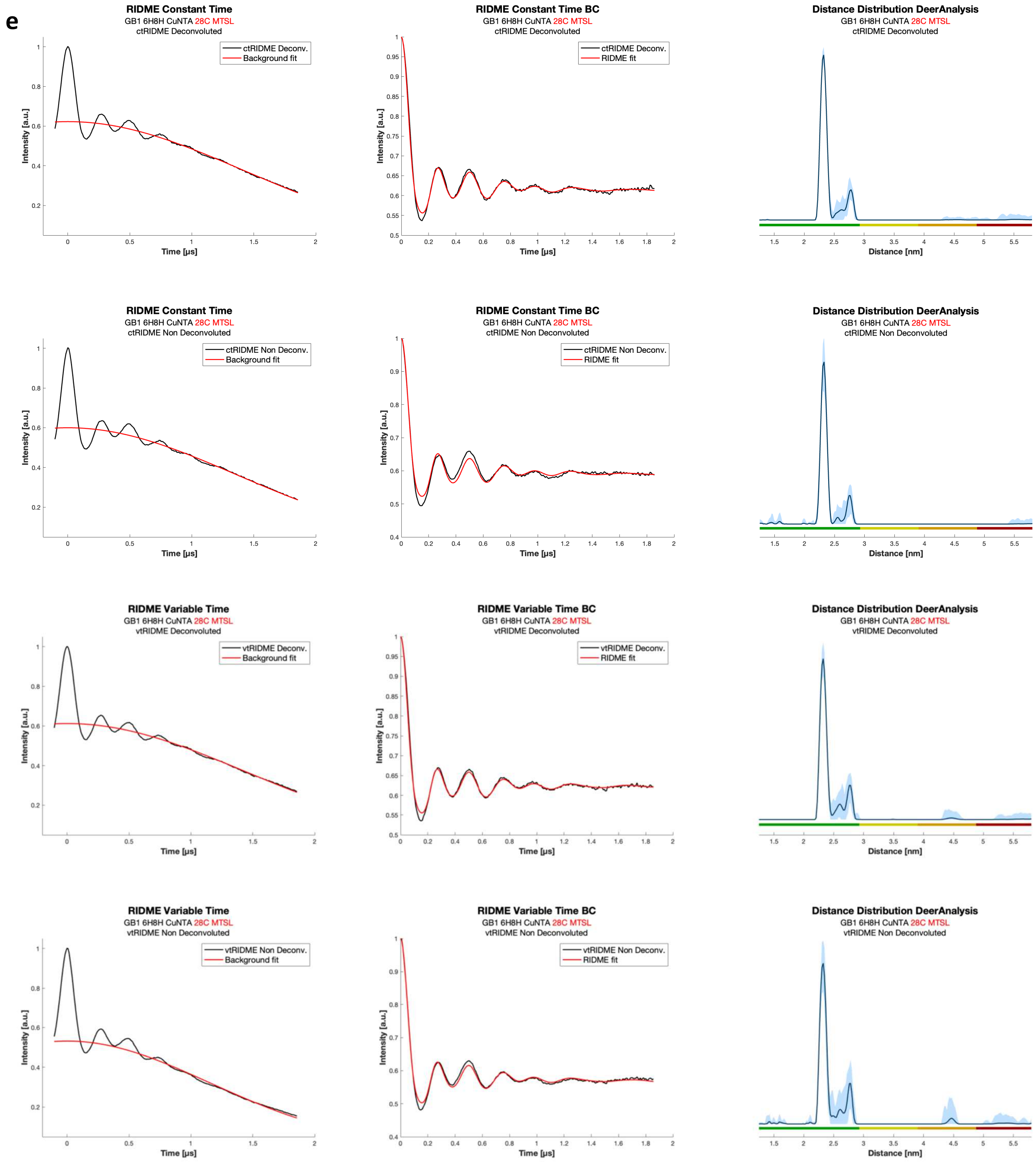

Fig. S4, continued Left: raw RIDME traces (black) with background function (red); middle: background-corrected (BC) data (black) with fit (red); right: corresponding distance distributions given as 95% confidence intervals (± 2σ) with 50% noise added for error estimation during statistical analysis. Colour bars represent reliability ranges (green: shape reliable; yellow: mean and width reliable; orange: mean reliable: red: no quantification possible). First row: ctRIDME deconvoluted, second row: ctRIDME non deconvoluted, third row: vtRIDME deconvoluted, fourth row: vtRIDME non deconvoluted **a)** GB1 I6C/K28H/Q32H MTSL CuNTA, **b)** GB1 I6C/K28H/Q32H MPSL CuNTA, **c)** GB1 I6C/K28H/Q32H IPSL CuNTA, **d)** GB1 I6C/K28H/Q32H IDSL CuNTA, **e)** GB1 I6H/N8H/K28C CuNTA MTSL, **f)** GB1 I6H/N8H/K28C CuNTA MPSL, **g)** GB1 I6H/N8H/K28C CuNTA IPSL, **h)** GB1 I6H/N8H/K28C CuNTA IDSL

f

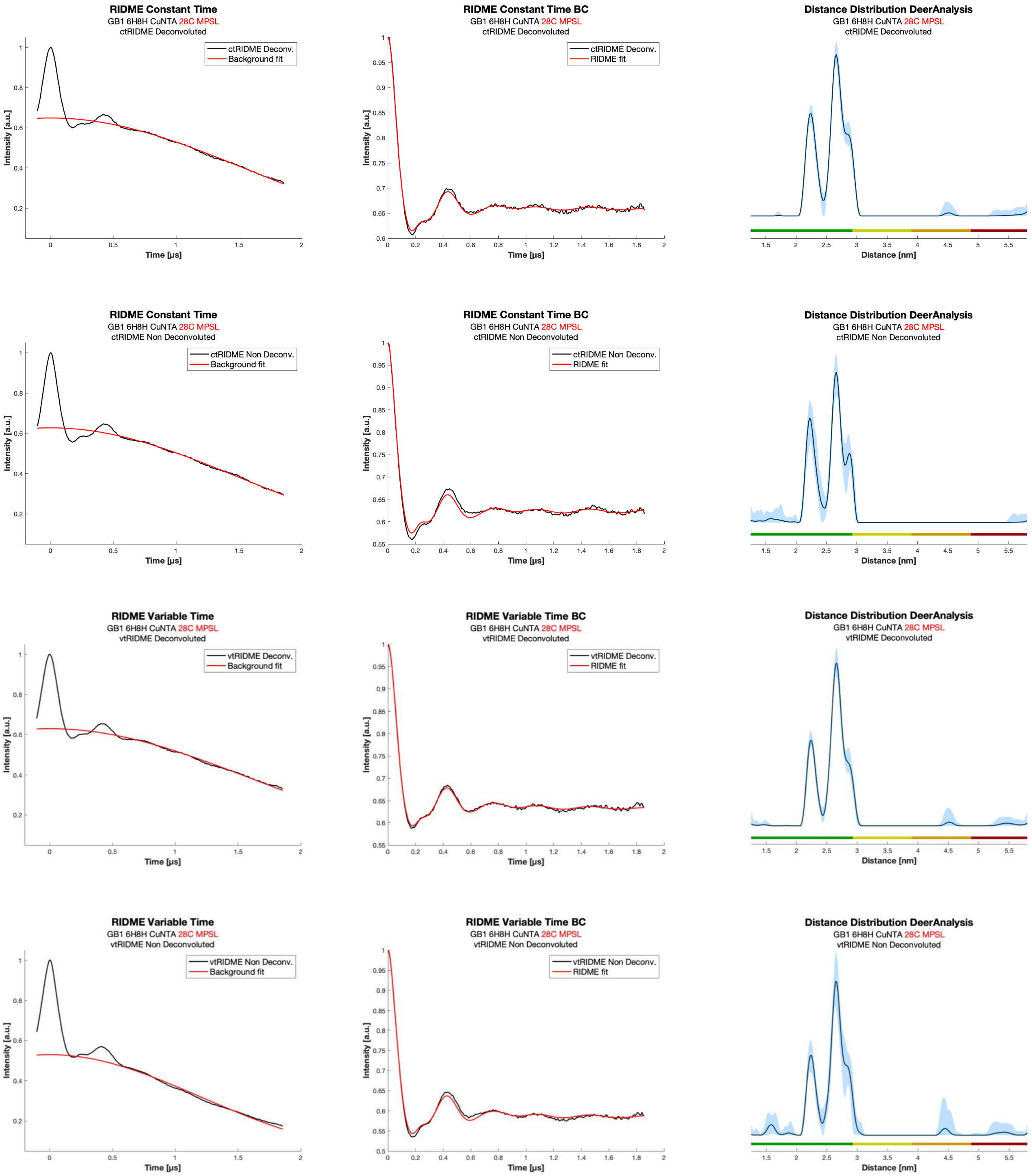

Fig. S4, continued Left: raw RIDME traces (black) with background function (red); middle: background-corrected (BC) data (black) with fit (red); right: corresponding distance distributions given as 95% confidence intervals (± 2σ) with 50% noise added for error estimation during statistical analysis. Colour bars represent reliability ranges (green: shape reliable; yellow: mean and width reliable; orange: mean reliable: red: no quantification possible). First row: ctRIDME deconvoluted, second row: ctRIDME non deconvoluted, third row: vtRIDME deconvoluted, fourth row: vtRIDME non deconvoluted **a)** GB1 I6C/K28H/Q32H MTSL CuNTA, **b)** GB1 I6C/K28H/Q32H MPSL CuNTA, **c)** GB1 I6C/K28H/Q32H IPSL CuNTA, **d)** GB1 I6C/K28H/Q32H IDSL CuNTA, **e)** GB1 I6H/N8H/K28C CuNTA MTSL, **f)** GB1 I6H/N8H/K28C CuNTA MPSL, **g)** GB1 I6H/N8H/K28C CuNTA IPSL, **h)** GB1 I6H/N8H/K28C CuNTA IDSL

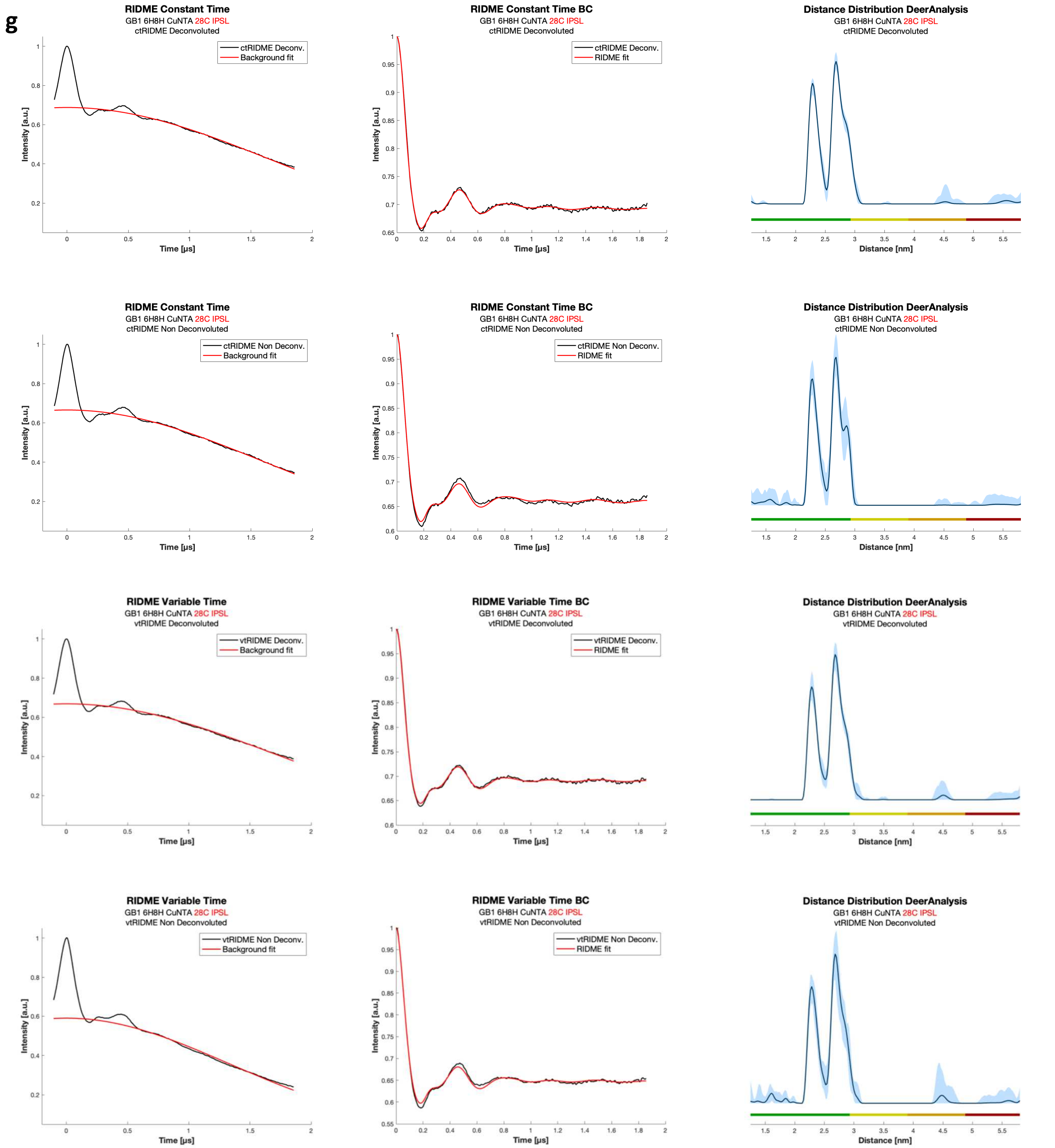

Fig. S4, continued Left: raw RIDME traces (black) with background function (red); middle: background-corrected (BC) data (black) with fit (red); right: corresponding distance distributions given as 95% confidence intervals (± 2σ) with 50% noise added for error estimation during statistical analysis. Colour bars represent reliability ranges (green: shape reliable; yellow: mean and width reliable; orange: mean reliable: red: no quantification possible). First row: ctRIDME deconvoluted, second row: ctRIDME non deconvoluted, third row: vtRIDME deconvoluted, fourth row: vtRIDME non deconvoluted **a)** GB1 I6C/K28H/Q32H MTSL CuNTA, **b)** GB1 I6C/K28H/Q32H MPSL CuNTA, **c)** GB1 I6C/K28H/Q32H IPSL CuNTA, **d)** GB1 I6C/K28H/Q32H IDSL CuNTA, **e)** GB1 I6H/N8H/K28C CuNTA MTSL, **f)** GB1 I6H/N8H/K28C CuNTA MPSL, **g)** GB1 I6H/N8H/K28C CuNTA IPSL, **h)** GB1 I6H/N8H/K28C CuNTA IDSL

h

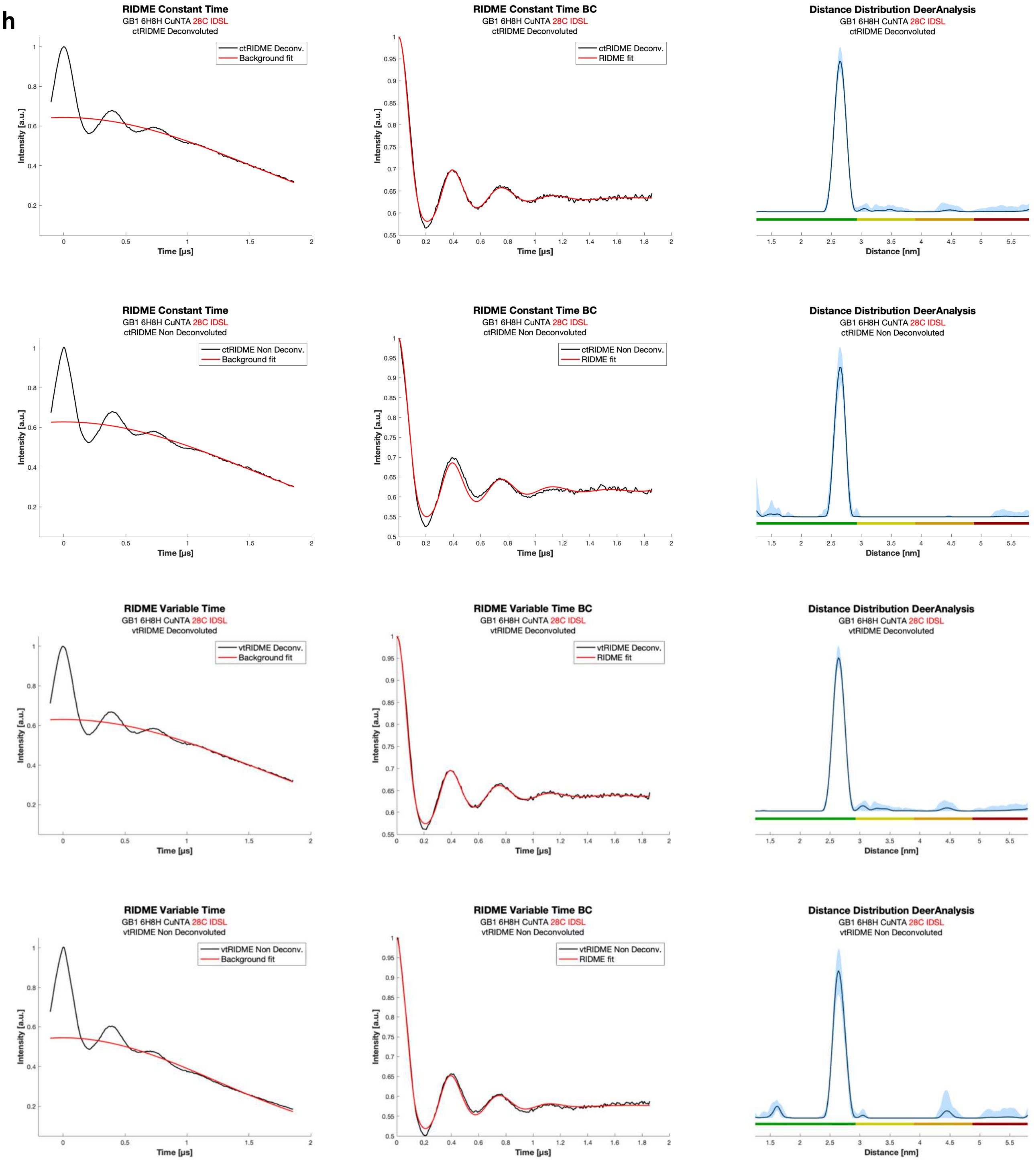

Fig. S4, continued Left: raw RIDME traces (black) with background function (red); middle: background-corrected (BC) data (black) with fit (red); right: corresponding distance distributions given as 95% confidence intervals (± 2σ) with 50% noise added for error estimation during statistical analysis. Colour bars represent reliability ranges (green: shape reliable; yellow: mean and width reliable; orange: mean reliable: red: no quantification possible). First row: ctRIDME deconvoluted, second row: ctRIDME non deconvoluted, third row: vtRIDME deconvoluted, fourth row: vtRIDME non deconvoluted **a)** GB1 I6C/K28H/Q32H MTSL CuNTA, **b)** GB1 I6C/K28H/Q32H MPSL CuNTA, **c)** GB1 I6C/K28H/Q32H IPSL CuNTA, **d)** GB1 I6C/K28H/Q32H IDSL CuNTA, **e)** GB1 I6H/N8H/K28C CuNTA MTSL, **f)** GB1 I6H/N8H/K28C CuNTA MPSL, **g)** GB1 I6H/N8H/K28C CuNTA IPSL, **h)** GB1 I6H/N8H/K28C CuNTA IDSL

1.5 Sensitivity data for vtRIDME and ctRIDME

We employed both ctRIDME and vtRIDME to compare the two pulse sequences in their performances for gaining confidence in the general applicability and robustness of the variable-time sequence. Therefore, the modulation depth extracted from each RIDME trace during processing in DeerAnalysis, divided by the noise (RMSD), calculated from the phase corrected imaginary part of the data (**Table S3**), were employed for obtaining the sensitivity per echo and the sensitivity per unit of time for each trace (**Table S4**) as reported previously[1]. As expected, higher sensitivity values were achieved from the vtRIDME, especially when considering the ratio between the non deconvoluted sequences.

ctRIDME vs vtRIDME

**Sensitivities**		**Δ**				**RMSD**				**S**			
		**ctRIDME**		**vtRIDME**		**ctRIDME**		**vtRIDME**		**ctRIDME**		**vtRIDME**	
		**Deconv.**	**Non Deconv.**	**Deconv.**	**Non Deconv.**	**Deconv.**	**Non Deconv.**	**Deconv.**	**Non Deconv.**	**Deconv.**	**Non Deconv.**	**Deconv.**	**Non Deconv.**
**MTSL**	6C	0.434	0.452	0.447	0.517	0.0030	0.0030	0.0024	0.0016	142.8	149.9	187.6	323.1
	28C	0.377	0.406	0.387	0.428	0.0023	0.0026	0.0020	0.0014	193.8	189.8	232.5	313.5
**MPSL**	6C	0.403	0.427	0.416	0.486	0.0021	0.0022	0.0018	0.0015	159.4	134.1	166.3	237.7
	28C	0.351	0.373	0.370	0.470	0.0020	0.0022	0.0018	0.0011	151.1	151.0	222.7	351.1
**IPSL**	6C	0.333	0.346	0.358	0.420	0.0021	0.0026	0.0021	0.0018	164.5	157.3	196.0	304.2
	28C	0.312	0.334	0.331	0.410	0.0017	0.0024	0.0015	0.0011	171.4	166.4	207.0	416.2
**IDSL**	6C	0.412	0.440	0.430	0.501	0.0027	0.0029	0.0019	0.0014	185.5	138.4	226.2	385.5
	28C	0.357	0.372	0.369	0.419	0.0024	0.0028	0.0025	0.0019	147.4	133.0	146.4	219.1

Table S3 Noise estimates (RMSD), modulation depths (Δ) and the sensitivity obtained from their ratio (S) for the two different RIDME pulse sequences (variable or constant time) with and without deconvolution for the two GB1 constructs (I6C/K28H/Q32H and I6H/N8H/K28C) with the four nitroxide labels, MTSL, MPSL, IPSL, IDSL

ctRIDME vs vtRIDME

Sensitivities		S_n				S_t				S_{ratio}	
		ctRIDME		vtRIDME		ctRIDME		vtRIDME			
		Deconv.	Non Deconv.	Deconv.	Non Deconv.	Deconv.	Non Deconv.	Deconv.	Non Deconv.	vt/ct Deconvoluted	vt/ct Non Deconvoluted
MTSL	6C	4.464	4.686	5.864	10.09	44.64	46.86	58.64	100.9	1.314	2.155
	28C	6.058	5.933	7.267	9,797	49.82	59.33	72.67	97.97	1.191	1.934
MPSL	6C	4.982	4.192	5.199	7.431	49.82	41.92	51.99	74.31	1.119	1.651
	28C	4.725	4,720	6,961	10,97	47.25	47.20	69.61	109.7	1.208	2.501
IPSL	6C	5.141	4.917	6.126	9.509	51.41	49.17	61.26	95.09	1.044	1.772
	28C	5.359	5.201	6.471	13.00	53.59	52.01	64.71	130.0	1.219	2.784
IDSL	6C	5.800	4.328	7.070	12.05	58.00	43.28	70.70	120.5	1.473	2.325
	28C	4.607	4.158	4.575	6.848	46.07	41.58	45.75	68.48	0.933	1.647

Table S4 Normalized sensitivity values (S_n) obtained from the sensitivity (S, **Table S3**) divided by the square root of total echoes per point (taken as the product of number of scans (1), shots per point (2), number of τ averages (16), and phase cycle(32)), and sensitivity per unit of time (S_t) obtained by multiplying S_n values for the square root of the averaging rate (100 Hz). The values were extracted for both the constant and variable time RIDME with and without deconvolution for both GB1 constructs I6C/K28H/Q32H and I6H/N8H/K28C with the four nitroxide labels MTSL, MPSL, IPSL and IDSL. S_{ratio} represents the ratio between the sensitivity values for the constant and variable time RIDME, respectively for the deconvoluted or non deconvoluted.

1.6 Distance distributions extracted from vtRIDME and ctRIDME with and without deconvolution

As expected from systems with short distances and relatively narrow distance distributions, the two different RIDME pulse sequences do not display significant discrepancies, neither in shape nor in mean and widths (**Table S4**), showing high consistency and robustness, regardless whether the deconvolution step has been applied or not. However, the distance distributions belonging to the vtRIDME without the deconvolution step (in green) are the most affected by the presence of artifacts at higher distance ranges. The superimposed distance distributions for both GB1 constructs, each one with the four distinct nitroxide labels, are reported in **Fig. S5**.

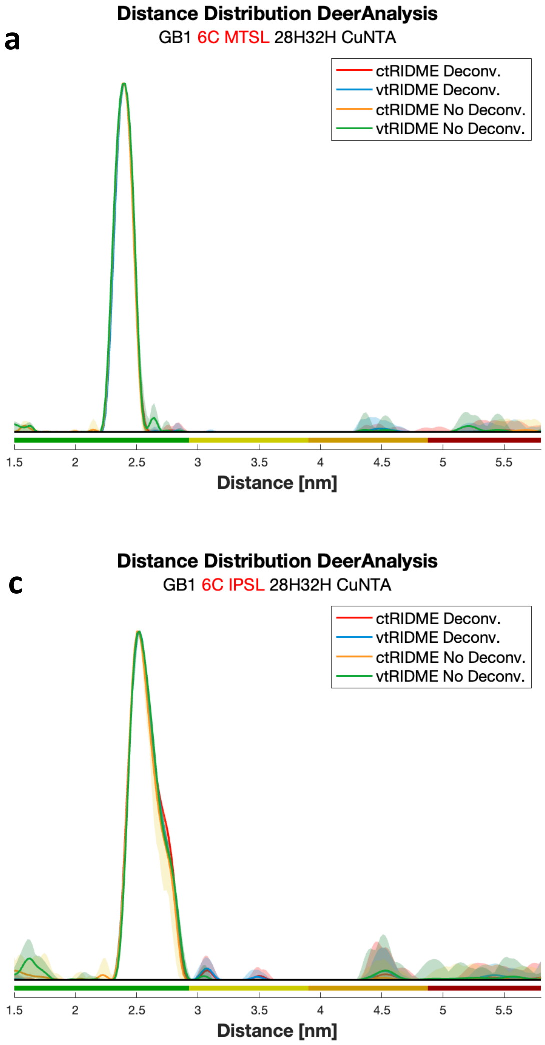

Fig. S5 Superimposed distance distributions extracted from ctRIDME and vtRIDME both deconvoluted and non deconvoluted traces, with the confidence estimates intervals (± 2σ) shown as shaded regions. Colour bars represent reliability ranges (green: shape reliable; yellow: mean and width reliable; orange: mean reliable: red: no quantification possible). **a)** GB1 I6C/K28H/Q32H MTSL CuNTA, **b)** GB1 I6C/K28H/Q32H MPSL CuNTA, **c)** GB1 I6C/K28H/Q32H IPSL CuNTA, **d)** GB1 I6C/K28H/Q32H IDSL CuNTA, **e)** GB1 I6H/N8H/K28C CuNTA MTSL, **f)** GB1 I6H/N8H/K28C CuNTA MPSL, **g)** GB1 I6H/N8H/K28C CuNTA IPSL, **h)** GB1 I6H/N8H/K28C CuNTA IDSL

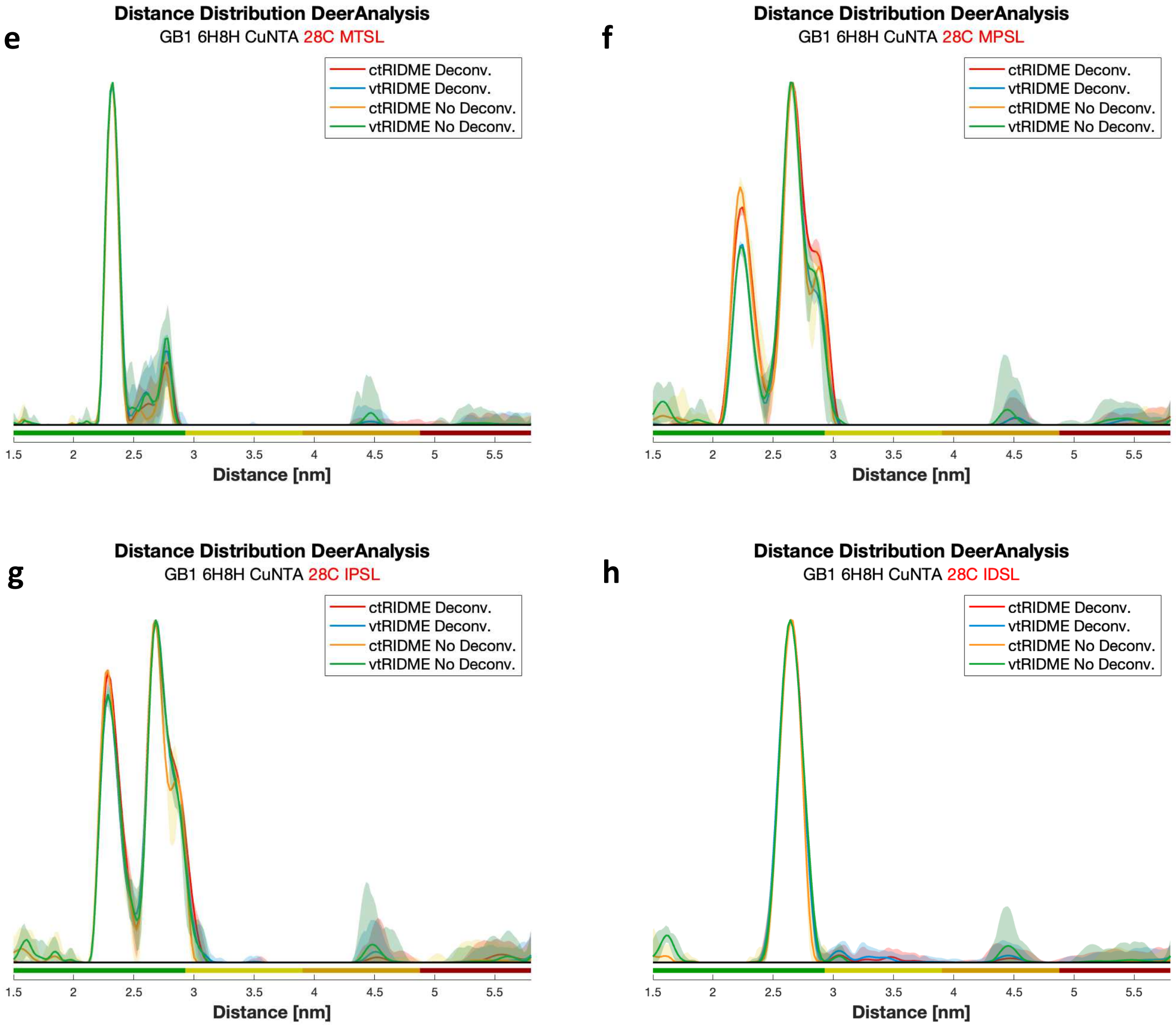

Fig. S5, continued Superimposed distance distributions extracted from ctRIDME and vtRIDME both deconvoluted and non deconvoluted traces, with the confidence estimates intervals (± 2σ) shown as shaded regions. Colour bars represent reliability ranges (green: shape reliable; yellow: mean and width reliable; orange: mean reliable: red: no quantification possible). **a)** GB1 I6C/K28H/Q32H MTSL CuNTA, **b)** GB1 I6C/K28H/Q32H MPSL CuNTA, **c)** GB1 I6C/K28H/Q32H IPSL CuNTA, **d)** GB1 I6C/K28H/Q32H IDSL CuNTA, **e)** GB1 I6H/N8H/K28C CuNTA MTSL, **f)** GB1 I6H/N8H/K28C CuNTA MPSL, **g)** GB1 I6H/N8H/K28C CuNTA IPSL, **h)** GB1 I6H/N8H/K28C CuNTA IDSL

1.7 Mean, Width and Full Width at Half Maximum for ctRIDME and vtRIDME both with and without deconvolution

All mean and width values of the experimental distance distributions were extracted with an in-house Matlab script. The values were calculated considering only the data between 1.7 and 4.5 nm to suppress the influence of artifacts at higher or lower distance ranges that greatly affect these values (**Table S5**). Mean values for the different experimental setups on the same sample can be considered consistent and robust with respect to each other. On the other hand, the vtRIDME suffers the most from the presence of artifacts which is reflected in the consistently higher values of the extracted widths.

ctRIDME vs vtRIDME		Mean (nm)				Width (nm)			
		ctRIDME		vtRIDME		ctRIDME		vtRIDME	
		Deconv.	Non Deconv.	Deconv.	Non Deconv.	Deconv.	Non Deconv.	Deconv.	Non Deconv.
**MTSL**	6C	2.402	2.390	2.412	2.407	0.131	0.062	0.189	0.159
	28C	2.404	2.382	2.424	2.453	0.185	0.154	0.244	0.341
**MPSL**	6C	2.674	2.653	2.662	2.657	0.168	0.115	0.171	0.124
	28C	2.581	2.552	2.591	2.611	0.272	0.245	0.261	0.324
**IPSL**	6C	2.596	2.574	2.601	2.601	0.185	0.149	0.208	0.212
	28C	2.592	2.565	2.600	2.607	0.256	0.233	0.277	0.317
**IDSL**	6C	2.561	2.515	2.596	2.589	0.259	0.133	0.333	0.319
	28C	2.682	2.641	2.692	2.705	0.217	0.091	0.250	0.308

Table S5 Mean and width values (reported in nm) extracted from experimental distance distributions of the two RIDME pulse sequences with and without deconvolution for both GB1 constructs (I6C/K28H/Q32H and I6H/N8H/K28C) for the four spin labels MTSL, MPSL, IPSL, IDSL

However, in some cases considering the restricted range between 1.7 and 4.5 nm is not enough to be completely independent from artifacts, and some values are still relatively affected by them, such as the width for the 28C IDSL. Therefore, we monitored the full width at half maximum (FWHM), considering this to be a parameter more independent from artifacts, for every distance distribution peak, to further point out the high consistency and robustness of the two different RIDME sequences (**Table S6**). Moreover, we decided to extract the mean and width values for every distance distribution considering the lower error estimate obtained from Deer Analysis (**Table S7**).

ctRIDME vs vtRIDME

FWHM (nm)		MTSL		MPSL		IPSL		IDSL	
		6C	28C	6C	28C	6C	28C	6C	28C
ctRIDME	Deconv.	0.179	0.125	0.221	0.688	0.285	0.669	0.133	0.229
	No Deconv.	0.180	0.120	0.227	0.590	0.256	0.672	0.128	0.212
vtRIDME	Deconv.	0.175	0.128	0.246	0.557	0.274	0.635	0.139	0.230
	No Deconv.	0.191	0.130	0.220	0.554	0.265	0.640	0.139	0.231

Table S6 Full width at half maximum (FWHM) (reported in nm) extracted from experimental distance distributions of the two RIDME pulse sequences with and without deconvolution for both GB1 constructs I6C/K28H/Q32H and I6H/N8H/K28C for the four spin labels MTSL, MPSL, IPSL, IDSL

ctRIDME vs vtRIDME		Mean LB (nm)				Width LB (nm)			
		ctRIDME		vtRIDME		ctRIDME		vtRIDME	
		Deconv.	Non Deconv.	Deconv.	Non Deconv.	Deconv.	Non Deconv.	Deconv.	Non Deconv.
**MTSL**	6C	2.393	2.390	2.395	2.391	0.063	0.060	0.062	0.063
	28C	2.361	2.340	2.350	2.339	0.135	0.102	0.113	0.108
**MPSL**	6C	2.669	2.655	2.655	2.653	0.107	0.111	0.111	0.114
	28C	2.576	2.557	2.582	2.561	0.241	0.235	0.219	0.219
**IPSL**	6C	2.578	2.556	2.577	2.579	0.114	0.100	0.115	0.113
	28C	2.581	2.553	2.579	2.566	0.233	0.228	0.226	0.222
**IDSL**	6C	2.488	2.489	2.492	2.504	0.047	0.047	0.051	0.072
	28C	2.647	2.641	2.644	2.647	0.085	0.071	0.084	0.084

Table S7 Mean and width values (reported in nm) extracted from the lower error bound (LB) estimate of the experimental distance distributions of the two RIDME pulse sequences with and without deconvolution for both GB1 constructs (I6C/K28H/Q32H and I6H/N8H/K28C) for the four spin labels MTSL, MPS, IPSL and IDSL

1.8 Comparison of the distance distributions between two nitroxides labels or between the copper(II) and the nitroxides

Data obtained previously on the double-cysteine GB1 construct (GB1 I6C/K28C) showed bimodality for the MTSL label and generally broader distributions for the MPSL and IPSL labels (**Fig. S6**). Distinguishing whether this behaviour depends on the nitroxide attached to the α-helix or the β-sheet was possible only after the introduction of the less conformationally flexible CuNTA chelator ligand alternatively on one labelling site of the protein. The reduction in the distribution widths, provoked by the rigidity of this bipedal ligand, entailed a significant improvement in the precision of the measured distances, and a consequent decrease in the ambiguity of the interpretation of the system behaviour, leading us to mark the helix site as the one responsible for the broadness and bimodality in the distance distributions.

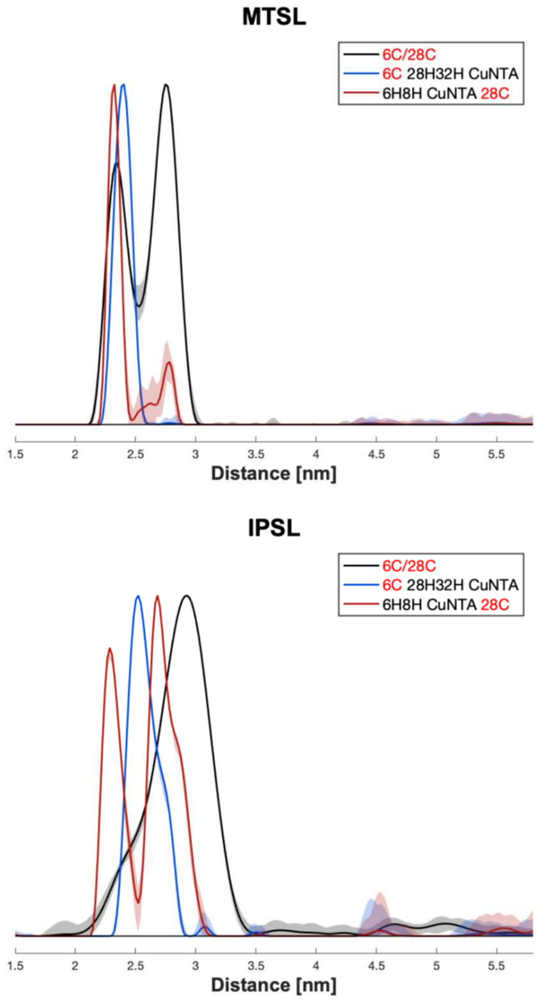

Fig. S6 Comparison of the distance distributions between two nitroxide spin labels on the double cysteine mutant GB1 I6C/K28C (black) obtained with the 4-pulse DEER sequence, and the distributions between the CuNTA and the same nitroxides, respectively on the I6C/K28H/Q32H (blue) and I6H/N8H/K28C (red) GB1 constructs obtained with the 5-pulse RIDME pulse sequence. The shadowed area represents the confidence estimate intervals (± 2σ)

1.9 Modelling with MMM and MtsslWizard

All the modelling data of the distance distributions, acquired for the different structure prediction tools (AlphaFold2 (**Fig. S7**), OmegaFold (**Fig. S8**), ESMFold (**Fig. S9**)), and the crystallographic structure (PDB:4wh4 (**Fig. S10**)), are reported here compared to the experimentally obtained distributions from the ctRIDME deconvoluted data set. All structural models behave in a similar manner, and the extensive discussion in the main manuscript about the AlphaFold2 performance can be transposed to the other prediction methods and to the crystallographic structure. Minor differences in terms of shapes of the distance distributions can be detected. Here we report a visual representation of the computated rotamers for the different labelling approaches and their respective different conditions (ambient and cryogenic temperature for MMM and Tight and Loose for MtsslWizard) for the nitroxides (**Fig. S11**) and for the CuNTA (Tight and Loose settings for the Wizard and as previously described for MMM [2]) (**Fig. S12**).

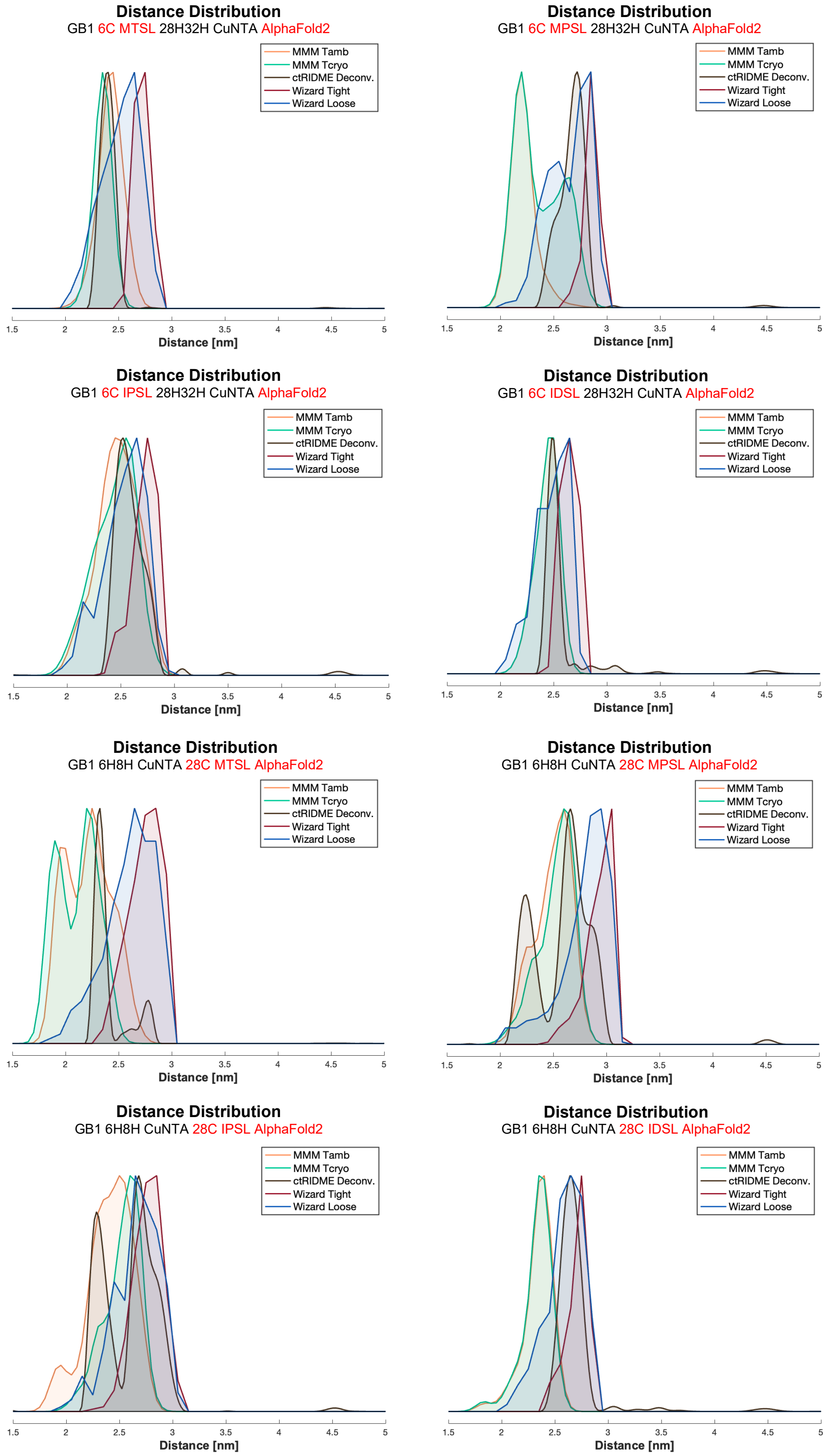

Fig. S7 Modelled distance distributions for both I6C/K28H/Q32H and the I6H/N8H/K28C GB1 constructs, both labelled with MTSL, MPSL, IPSL and IDSL, based on the AlphaFold2 structure, superimposed with their respective experimental distance distributions (derived from ctRIDME deconvoluted data set, in black). The *in silico* approaches considered are MMM at ambient (orange) and cryogenic (green) temperature and MtsslWizard with Tight (red) and Loose (blue) settings. First row: 6C MTSL, 6C MPSL. Second row: 6C IPSL, 6C IDSL. Third row: 28C MTSL and 28C MPSL. Fourth row: 28C IPSL and 28C IDSL

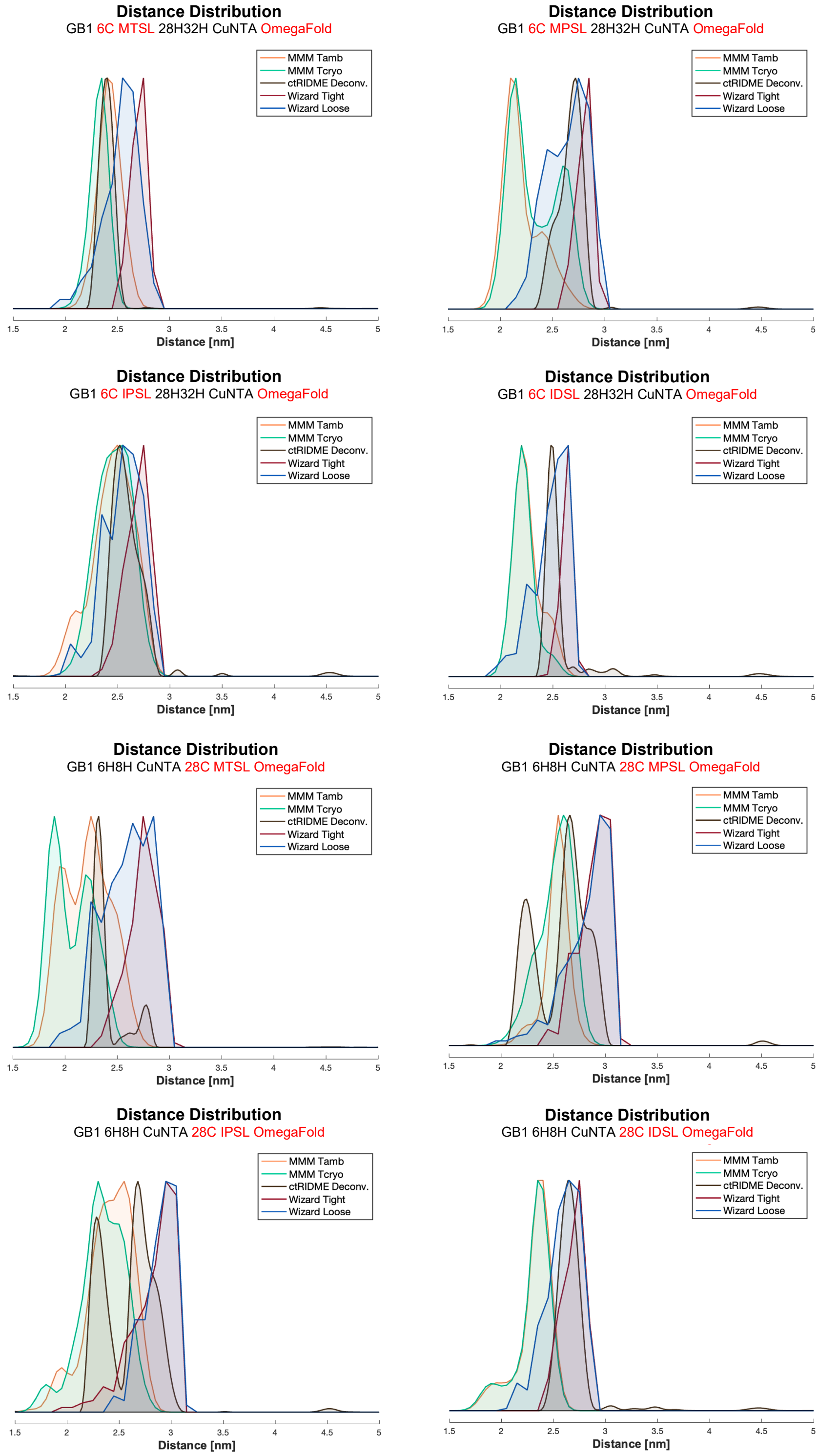

Fig. S8 Modelled distance distributions for both I6C/K28H/Q32H and the I6H/N8H/K28C GB1 constructs, both labelled with MTSL, MPSL, IPSL and IDSL, based on the OmegaFold structure, superimposed with their respective experimental distance distributions (derived from ctRIDME deconvoluted data set, in black). The *in silico* approaches considered are MMM at ambient (orange) and cryogenic (green) temperature and MtsslWizard with Tight (red) and Loose (blue) settings. First row: 6C MTSL, 6C MPSL. Second row: 6C IPSL, 6C IDSL. Third row: 28C MTSL and 28C MPSL. Fourth row: 28C IPSL and 28C IDSL

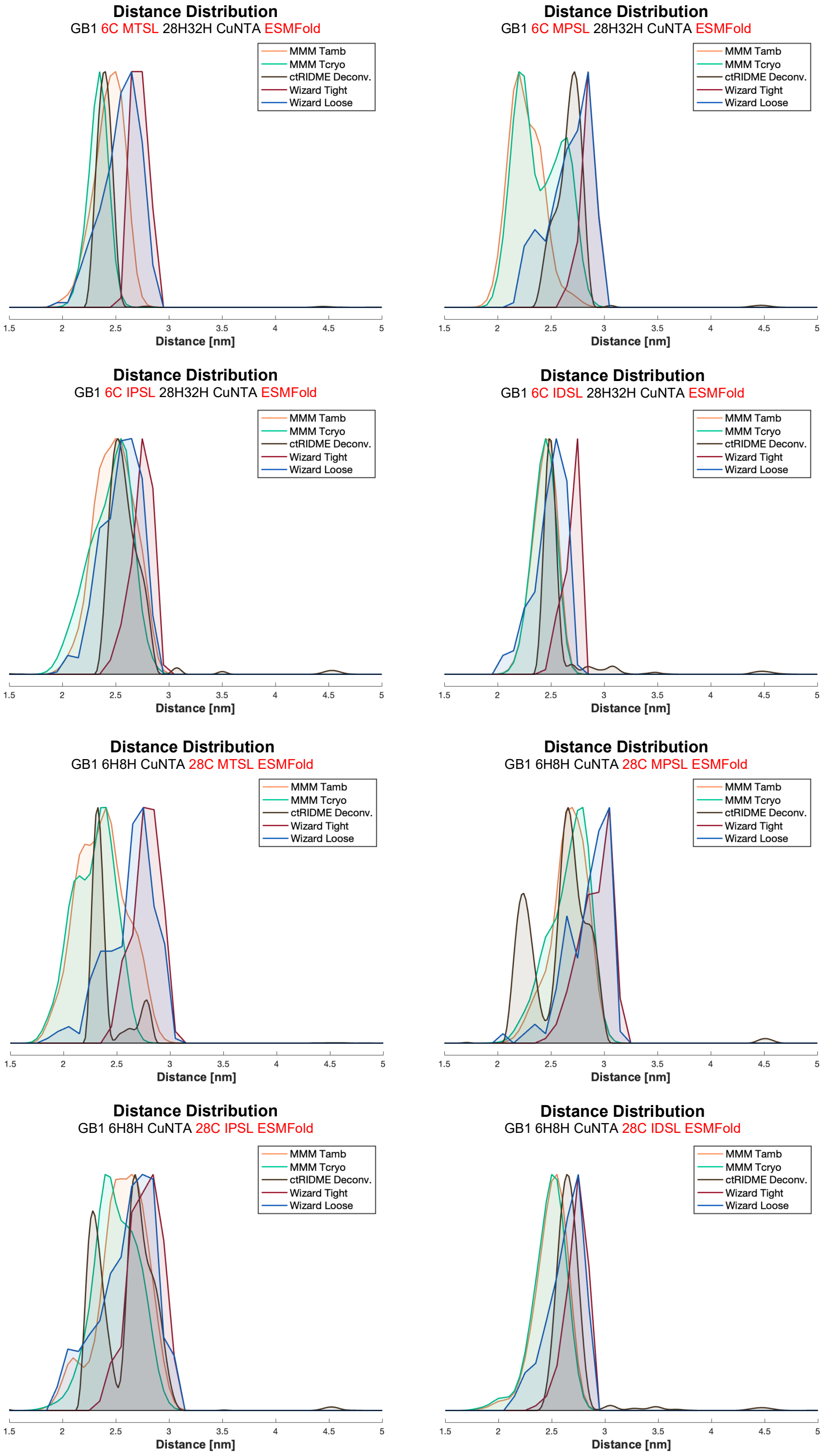

Fig. S9 Modelled distance distributions for both I6C/K28H/Q32H and the I6H/N8H/K28C GB1 constructs, both labelled with MTSL, MPSL, IPSL and IDSL, based on the ESMFold structure, superimposed with their respective experimental distance distributions (derived from ctRIDME deconvoluted data set, in black). The *in silico* approaches considered are MMM at ambient (orange) and cryogenic (green) temperature and MtsslWizard with Tight (red) and Loose (blue) settings. First row: 6C MTSL, 6C MPSL. Second row: 6C IPSL, 6C IDSL. Third row: 28C MTSL and 28C MPSL. Fourth row: 28C IPSL and 28C IDSL

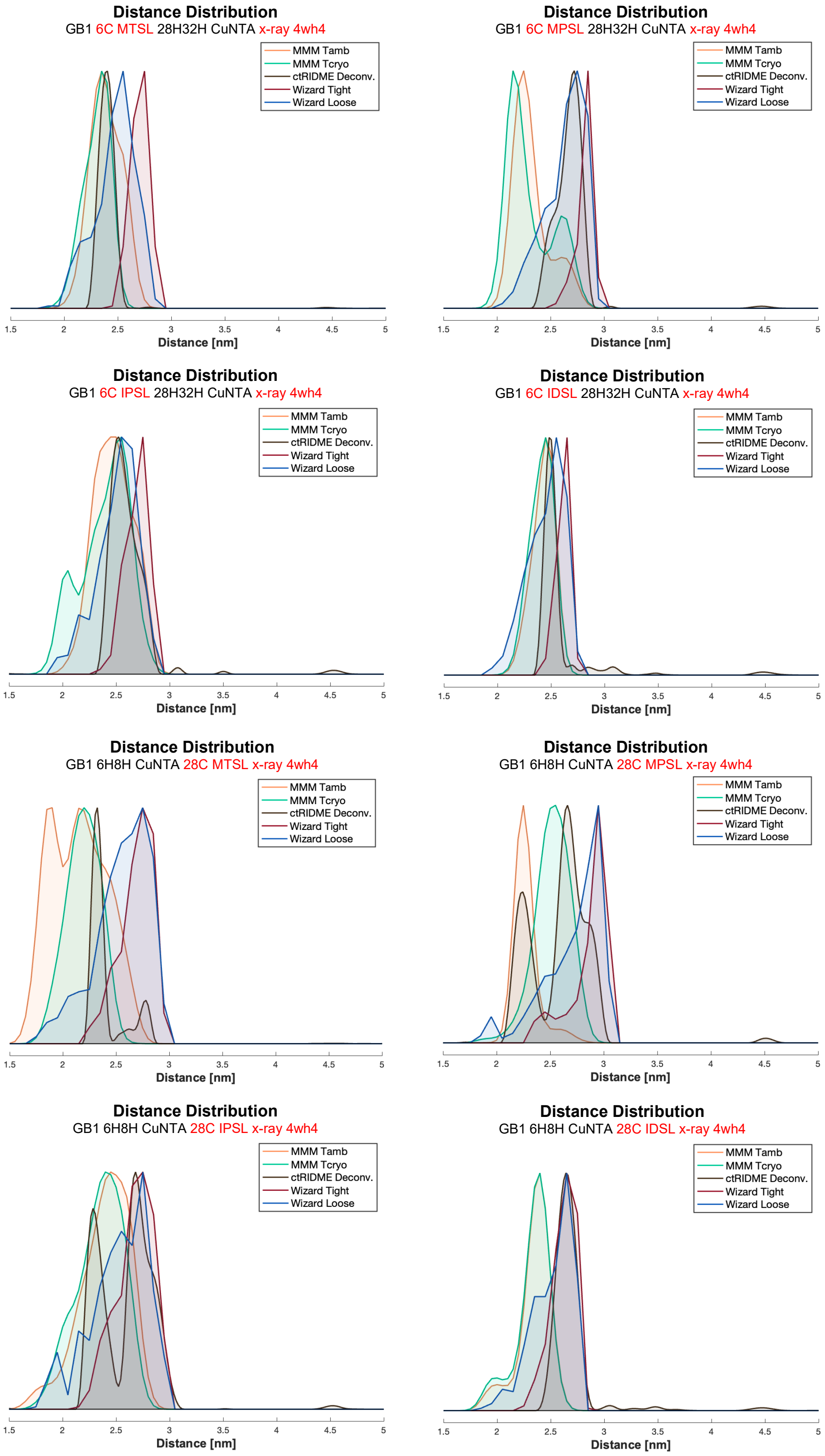

Fig. S10 Modelled distance distributions for both I6C/K28H/Q32H and the I6H/N8H/K28C GB1 constructs, both labelled with MTSL, MPSL, IPSL and IDSL, based on the X-ray structure (PDB: 4wh4), superimposed with their respective experimental distance distributions (derived from ctRIDME deconvoluted data set, in black). The *in silico* approaches considered are MMM at ambient (orange) and cryogenic (green) temperature and MtsslWizard with Tight (red) and Loose (blue) settings. First row: 6C MTSL, 6C MPSL. Second row: 6C IPSL, 6C IDSL. Third row: 28C MTSL and 28C MPSL. Fourth row: 28C IPSL and 28C IDSL

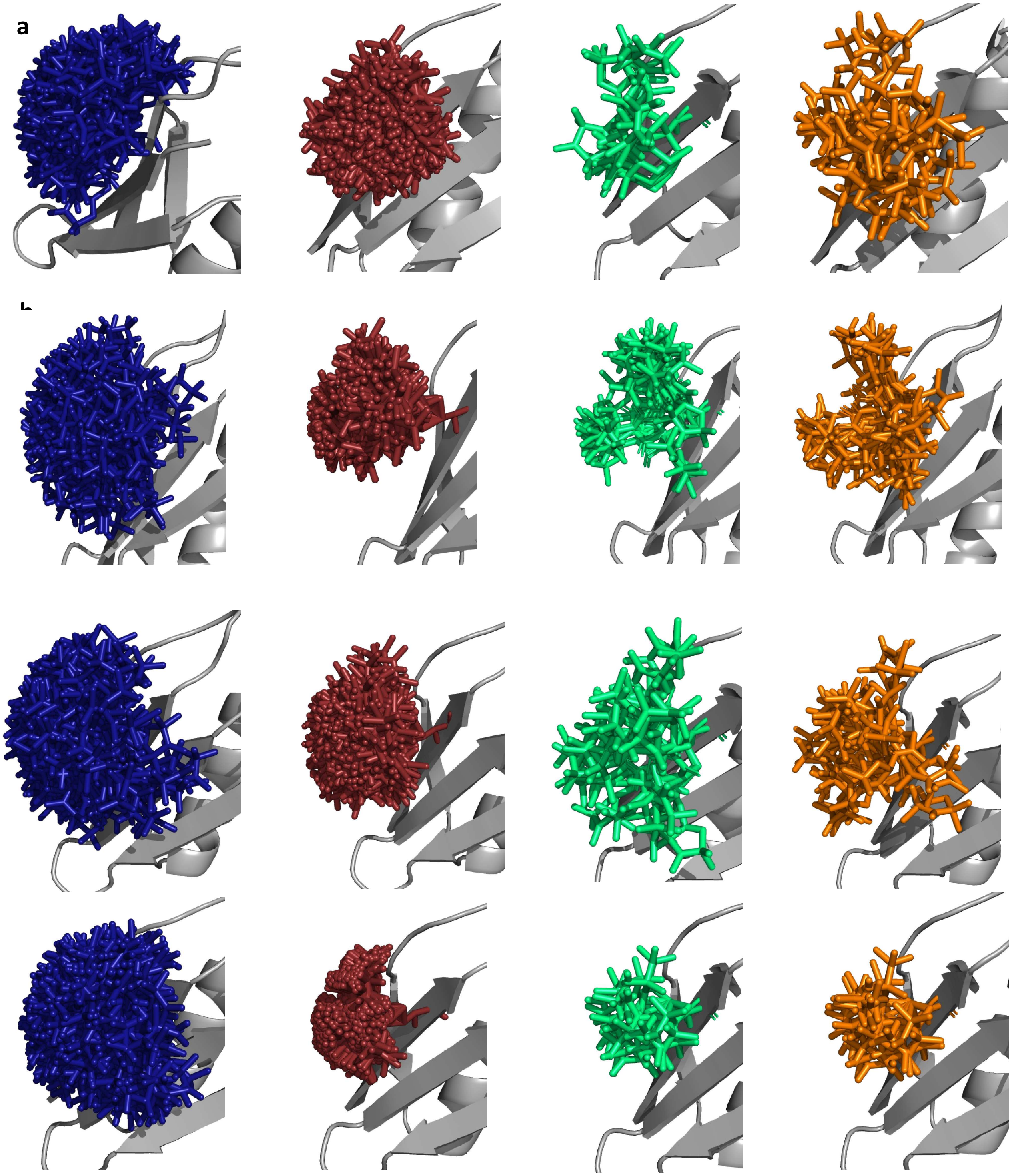

Fig. S11 Modelled rotamers for the two in silico labelling approaches, MMM with cryogenic (green rotamers) and ambient (orange rotamers) conditions, and MtsslWizard with Tight (red rotamers) and Loose (blue rotamers) settings, for both I6C/K28H/Q32H and the I6H/N8H/K28C GB1 constructs, labelled with MTSL, MPSL, IPSL and IDSL. **a)** GB1 I6C/K28H/Q32H MTSL, **b)** GB1 I6C/K28H/Q32H MPSL, **c)** GB1 I6C/K28H/Q32H IPSL, **d)** GB1 I6C/K28H/Q32H IDSL, **e)** GB1 I6H/N8H/K28C MTSL, **f)** GB1 I6H/N8H/K28C MPSL, **g)** GB1 I6H/N8H/K28C IPSL, **h)** GB1 I6H/N8H/K28C IDSL

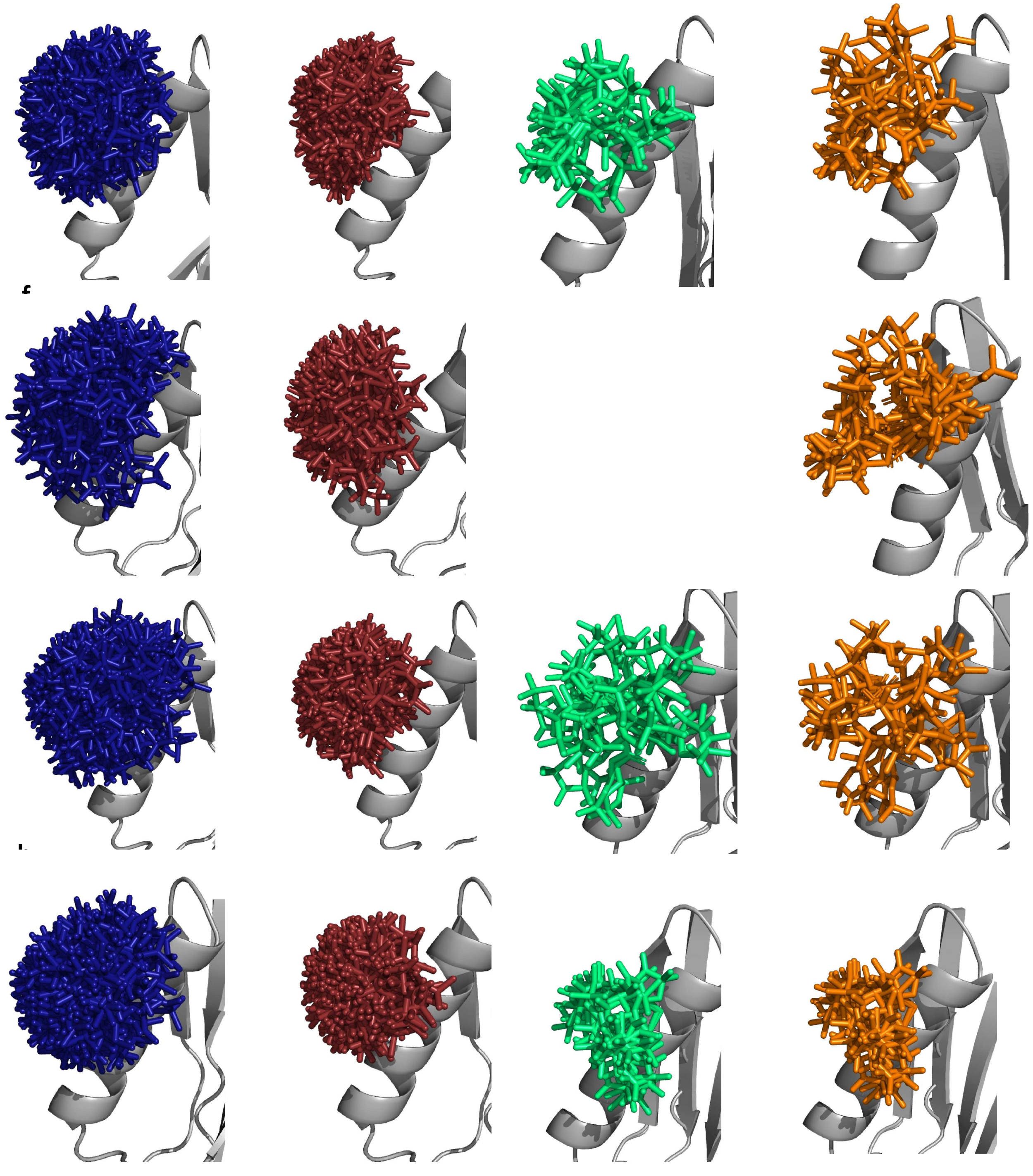

Fig. S11, continued Modelled rotamers for the two in silico labelling approaches, MMM with cryogenic (green rotamers) and ambient (orange rotamers) conditions, and MtsslWizard with Tight (red rotamers) and Loose (blue rotamers) settings, for both I6C/K28H/Q32H and the I6H/N8H/K28C GB1 constructs, labelled with MTSL, MPSL, IPSL and IDSL. **a)** GB1 I6C/K28H/Q32H MTSL, **b)** GB1 I6C/K28H/Q32H MPSL, **c)** GB1 I6C/K28H/Q32H IPSL, **d)** GB1 I6C/K28H/Q32H IDSL, **e)** GB1 I6H/N8H/K28C MTSL, **f)** GB1 I6H/N8H/K28C MPSL, **g)** GB1 I6H/N8H/K28C IPSL, **h)** GB1 I6H/N8H/K28C IDSL

a

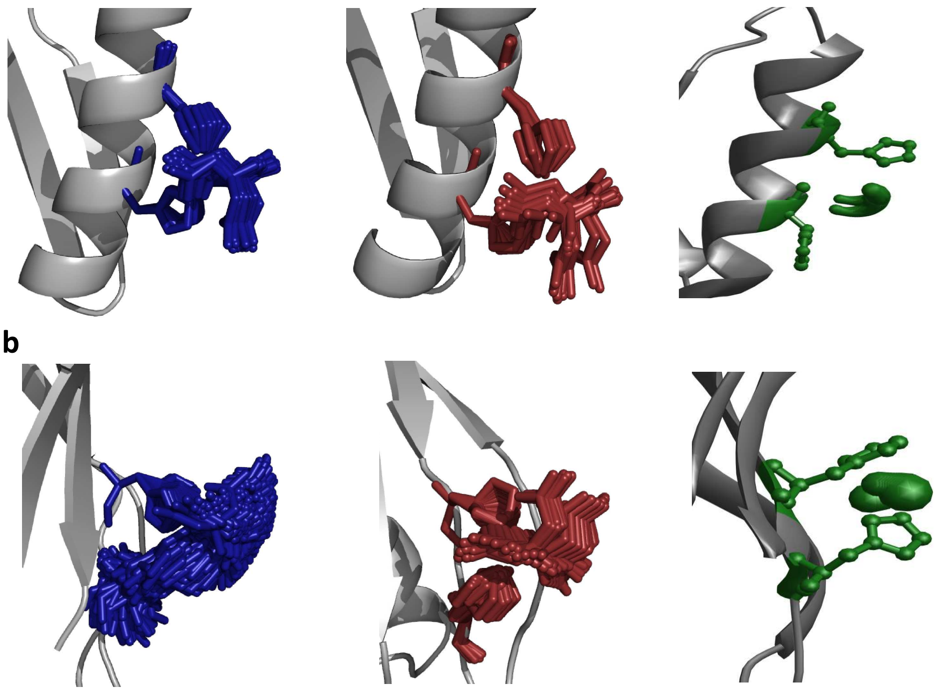

b

Fig. S12 Modelled rotamers for the CuNTA label for the two *in silico* labelling approaches MtsslWizard Tight (red rotamers) and Loose (blue rotamers) settings and for MMM (forest green rotamers) for both I6C/K28H/Q32H (**a**) and I6H/N8H/K28C (**b**) GB1 constructs

1.10 Correlation plots and mean, widths values of the modelled distributions

Correlation plots (**Fig. S13** and **Fig. S14**) were obtained by extracting the mean and width of every distance distribution from the experimental and the *in silico* labelling data (**Table S5**). To get a numerical quantification of the curve discrepancies, the differences between the experimental and *in silico* mean values were also extracted as absolute values (Δ mean) (**Tables S8-S11**). The correlation plots were built considering the mean of the experimental distances on the x-axis and the mean of the *in silico* labelling on the y-axis. The widths of the experimental and simulated distributions were employed as "error bars" for each data point.

AlphaFold2		Mean				Width				Δ Mean			
		MMM		MtsslWizard		MMM		MtsslWizard		MMM		MtsslWizard	
		T_{cryo}	T_{amb}	Tight	Loose	T_{cryo}	T_{amb}	Tight	Loose	T_{cryo}	T_{amb}	Tight	Loose
MTSL	6C	2.349	2.425	2.720	2.517	0.089	0.123	0.074	0.184	0.052	0.023	0.318	0.115
	28C	2.108	2.217	2.746	2.606	0.196	0.220	0.152	0.235	0.295	0.186	0.341	0.202
MPSL	6C	2.364	2.213	2.852	2.643	0.223	0.123	0.070	0.195	0.310	0.460	0.177	0.031
	28C	2.525	2.499	2.930	2.795	0.173	0.173	0.132	0.229	0.056	0.082	0.348	0.213
IPSL	6C	2.365	2.473	2.718	2.528	0.223	0.197	0.115	0.199	0.232	0.123	0.121	0.068
	28C	2.525	2.408	2.768	2.659	0.173	0.217	0.140	0.216	0.066	0.183	0.175	0.067
IDSL	6C	2.436	2.437	2.645	2.478	0.115	0.115	0.079	0.159	0.124	0.123	0.084	0.082
	28C	2.331	2.339	2.714	2.591	0.148	0.148	0.105	0.176	0.350	0.342	0.028	0.090

Table S8 Mean, width and Δ mean of the modelled distances for both MMM (ambient and cryogenic temperature) and MtsslWizard (Tight and Loose settings), for both GB1 constructs I6C/K28H/Q32H and I6H/N8H/K28C, each labelled with the four nitroxides (MTSL, MPSL, IPSL and IDSL), based on the AlphaFold2 structure

OmegaFold		Mean				Width				Δ Mean			
		MMM		MtsslWizard		MMM		MtsslWizard		MMM		MtsslWizard	
		T_{cryo}	T_{amb}	Tight	Loose	T_{cryo}	T_{amb}	Tight	Loose	T_{cryo}	T_{amb}	Tight	Loose
MTSL	6C	2.323	2.424	2.704	2.534	0.091	0.114	0.077	0.175	0.078	0.022	0.301	0.132
	28C	2.071	2.283	2.746	2.594	0.200	0.194	0.146	0.234	0.332	0.121	0.341	0.189
MPSL	6C	2.333	2.212	2.805	2.638	0.237	0.188	0.075	0.193	0.340	0.462	0.130	0.036
	28C	2.521	2.533	2.888	2.824	0.171	0.125	0.149	0.222	0.060	0.048	0.306	0.242
IPSL	6C	2.466	2.451	2.690	2.547	0.171	0.208	0.115	0.187	0.130	0.145	0.093	0.049
	28C	2.342	2.410	2.753	2.609	0.219	0.223	0.145	0.239	0.249	0.181	0.161	0.017
IDSL	6C	2.223	2.257	2.631	2.479	0.115	0.138	0.051	0.167	0.337	0.303	0.070	0.081
	28C	2.308	2.317	2.690	2.595	0.177	0.175	0.109	0.168	0.373	0.365	0.079	0.086

Table S9 Mean, width and Δ mean of the modelled distances for both MMM (ambient and cryogenic temperature) and MtsslWizard (Tight and Loose settings), for both GB1 constructs I6C/K28H/Q32H and I6H/N8H/K28C, each labelled with the four nitroxides (MTSL, MPSL, IPSL and IDSL), based on the OmegaFold structure

ESMFold		Mean				Width				Δ Mean			
		MMM		MtsslWizard		MMM		MtsslWizard		MMM		MtsslWizard	
		T_{cryo}	T_{amb}	Tight	Loose	T_{cryo}	T_{amb}	Tight	Loose	T_{cryo}	T_{amb}	Tight	Loose
MTSL	6C	2.334	2.434	2.723	2.547	0.096	0.147	0.075	0.178	0.067	0.032	0.320	0.145
	28C	2.283	2.361	2.776	2.626	0.194	0.123	0.130	0.229	0.121	0.042	0.371	0.222
MPSL	6C	2.395	2.267	2.846	2.667	0.221	0.161	0.076	0.203	0.279	0.407	0.171	0.007
	28C	2.663	2.667	2.913	2.844	0.191	0.172	0.152	0.204	0.081	0.085	0.332	0.263
IPSL	6C	2.443	2.490	2.742	2.547	0.199	0.186	0.104	0.181	0.152	0.106	0.145	0.048
	28C	2.503	2.561	2.772	2.601	0.209	0.229	0.154	0.264	0.088	0.030	0.180	0.089
IDSL	6C	2.433	2.440	2.691	2.486	0.110	0.113	0.077	0.151	0.127	0.120	0.130	0.075
	28C	2.437	2.490	2.727	2.622	0.163	0.158	0.104	0.169	0.208	0.191	0.045	0.059

Table S10 Mean, width and Δ mean of the modelled distances for both MMM (ambient and cryogenic temperature) and MtsslWizard (Tight and Loose settings), for both GB1 constructs I6C/K28H/Q32H and I6H/N8H/K28C, each labelled with the four nitroxides (MTSL, MPSL, IPSL and IDSL), based on the ESMFold structure

X-ray		Mean				Width				Δ Mean			
		MMM		MtsslWizard		MMM		MtsslWizard		MMM		MtsslWizard	
		T_{cryo}	T_{amb}	Tight	Loose	T_{cryo}	T_{amb}	Tight	Loose	T_{cryo}	T_{amb}	Tight	Loose
MTSL	6C	2.304	2.391	2.703	2.472	0.127	0.148	0.084	0.191	0.097	0.010	0.300	0.070
	28C	2.193	2.151	2.689	2.552	0.163	0.263	0.157	0.257	0.210	0.252	0.285	0.148
MPSL	6C	2.307	2.319	2.822	2.634	0.219	0.176	0.076	0.188	0.367	0.355	0.147	0.040
	28C	2.519	2.272	2.860	2.721	0.171	0.125	0.186	0.269	0.062	0.307	0.278	0.139
IPSL	6C	2.394	2.477	2.691	2.508	0.228	0.183	0.108	0.195	0.201	0.118	0.094	0.088
	28C	2.351	2.394	2.673	2.522	0.217	0.233	0.173	0.271	0.240	0.198	0.080	0.069
IDSL	6C	2.413	2.428	2.615	2.454	0.113	0.109	0.063	0.170	0.148	0.133	0.054	0.106
	28C	2.332	2.346	2.620	2.518	0.166	0.158	0.115	0.188	0.349	0.335	0.061	0.164

Table S11 Mean, width and Δ mean of the modelled distances for both MMM (ambient and cryogenic temperature) and MtsslWizard (Tight and Loose settings), for both GB1 constructs I6C/K28H/Q32H and I6H/N8H/K28C, each labelled with the four nitroxides (MTSL, MPSL, IPSL and IDSL), based on the X-ray crystallographic structure (PDB: 4wh4)

A first set of correlation plots investigates the dependence on the labelling approach (MtsslWizard Tight or Loose and MMM ambient or cryogenic temperature) of the four different nitroxide labels for the same structure prediction method.

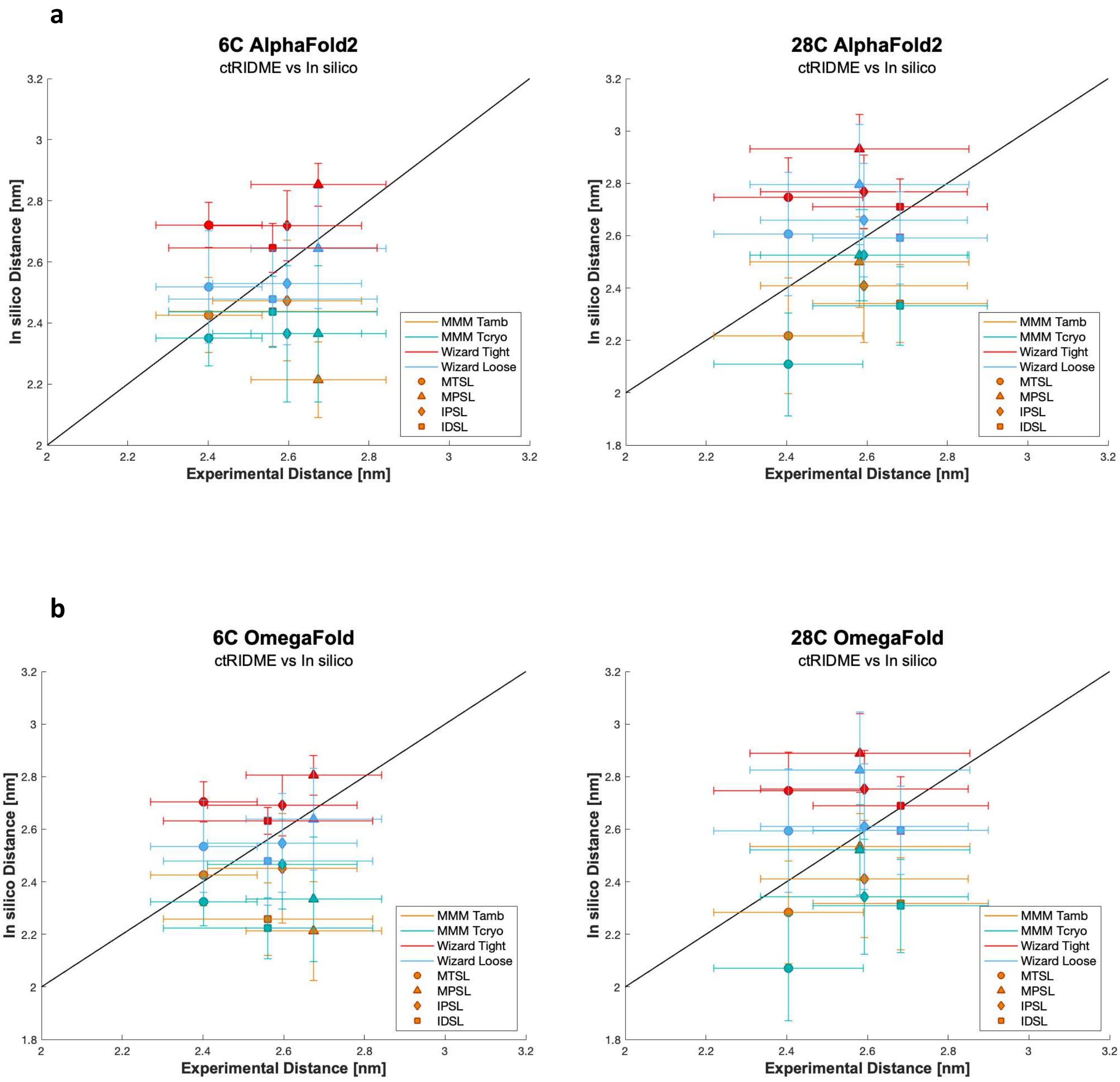

Fig. S13 Correlation plots, each point was plotted considering the mean value of the experimental distance distribution of the ctRIDME for the x-axis and their respective *in silico* mean values on the y axis. The error bars of each data point are derived from the experimental distance distribution width on the x axis and the respective *in silico* width on the y axis. The black line scores for the experimental trend. The different colors represent the different labelling approaches at different conditions: MMM at ambient temperature (orange), MMM at cryogenic temperature (green), MtsslWizard with Tight settings (red), MtsslWizard with Loose settings (blue). The different shapes correspond to the different nitroxide labels: circle MTSL, triangle MPSL, diamond IPSL and square IDSL. **a)** AlphaFold2, **b)** OmegaFold, **c)** ESMFold, **d)** X-ray (PDB: 4wh4)

c

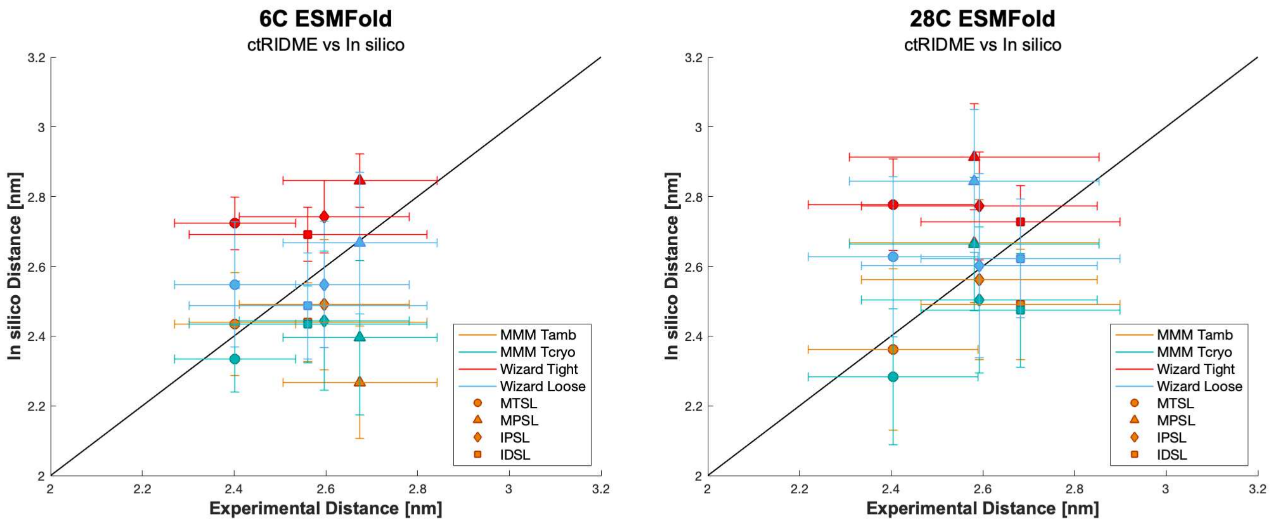

d

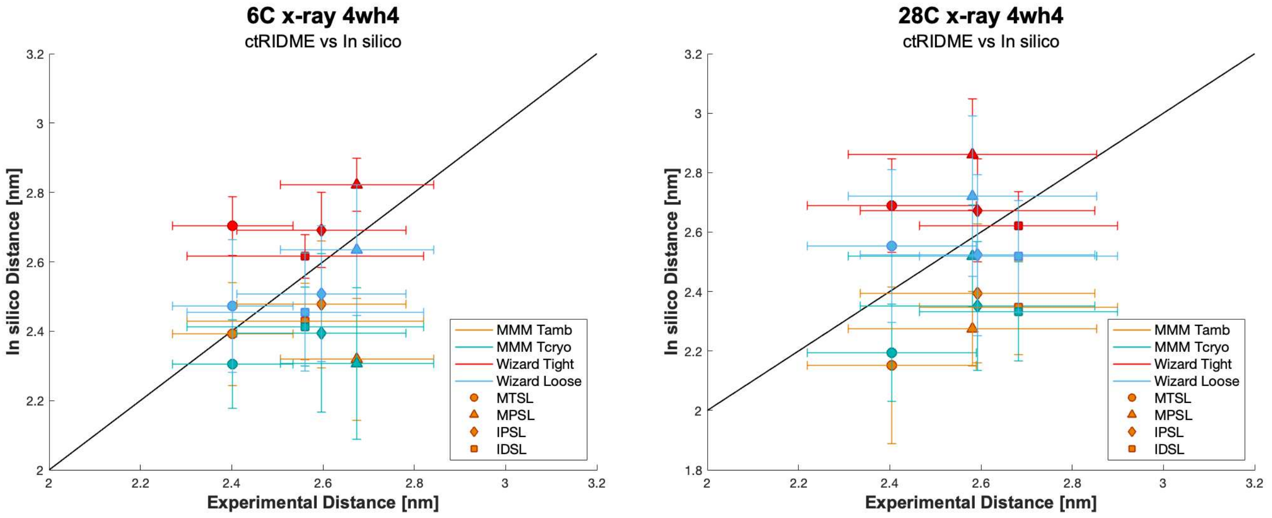

Fig. S13, continued Correlation plots, each point was plotted considering the mean value of the experimental distance distribution of the ctRIDME for the x-axis and their respective *in silico* mean values on the y axis. The error bars of each data point are derived from the experimental distance distribution width on the x axis and the respective *in silico* width on the y axis. The black line scores for the experimental trend. The different colors represent the different labelling approaches at different conditions: MMM at ambient temperature (orange), MMM at cryogenic temperature (green), MtsslWizard with Tight settings (red), MtsslWizard with Loose settings (blue). The different shapes correspond to the different nitroxide labels: circle MTSL, triangle MPSL, diamond IPSL and square IDSL. **a)** AlphaFold2, **b)** OmegaFold, **c)** ESMFold, **d)** X-ray (PDB: 4wh4)

A second set of correlation plots was introduced to better understand the role of the structure prediction methods with respect to the different labelling approaches for the same nitroxide label on the same GB1 construct.

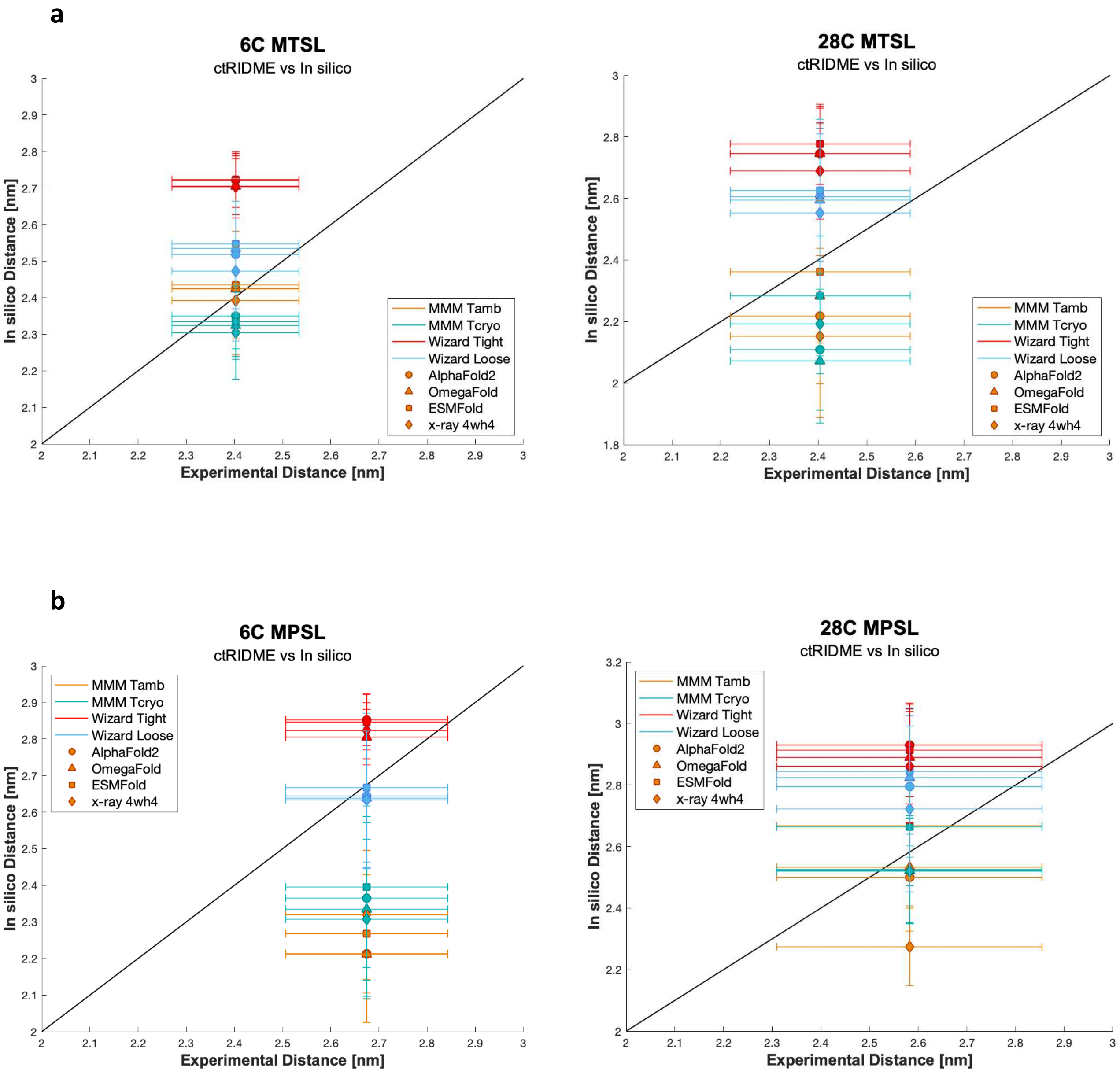

Fig. S14 Correlation plots, each point was plotted considering the mean value of the experimental distance distribution of the ctRIDME for the x-axis and their respective *in silico* mean values on the y axis. The error bars of each data point derive from the experimental distance distribution width on the x axis and the respective *in silico* width on the y axis. The black line scores for the experimental trend. The different colors represent the different labelling approaches at different conditions: MMM at ambient temperature (orange), MMM at cryogenic temperature (green), MtsslWizard with Tight settings (red), MtsslWizard with Loose settings (blue). The different shapes correspond to the different structure predictor tools: circle AlphaFold2, triangle OmegaFold, diamond X-ray (PDB: 4wh4) and square ESM-Fold. **a)** 6C and 28C MTSL, **b)** 6C and 28C MPSL, **c)** 6C and 28C IPSL, **d)** 6C and 28C IDSL.

c

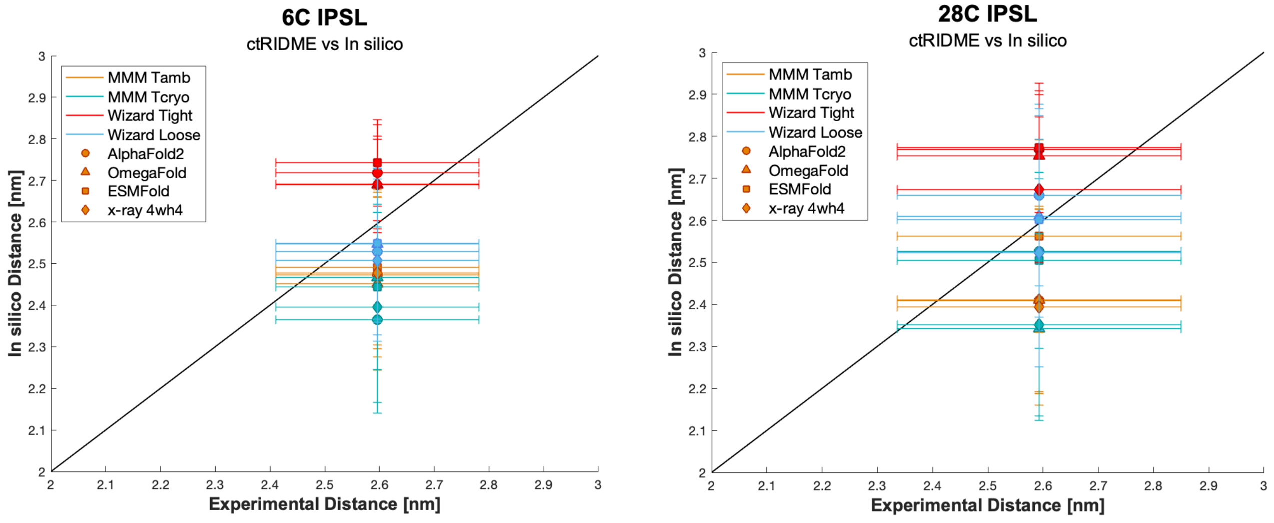

d

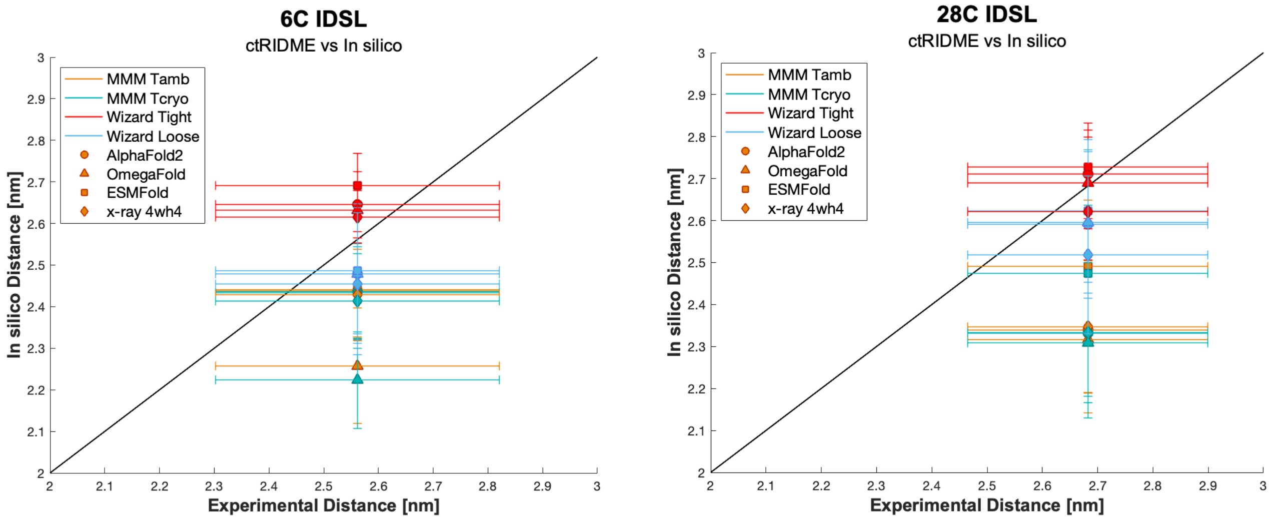

Fig. S14, continued Correlation plots, each point was plotted considering the mean value of the experimental distance distribution of the ctRIDME for the x-axis and their respective *in silico* mean values on the y axis. The error bars of each data point derive from the experimental distance distribution width on the x axis and the respective *in silico* width on the y axis. The black line scores for the experimental trend. The different colors represent the different labelling approaches at different conditions: MMM at ambient temperature (orange), MMM at cryogenic temperature (green), MtsslWizard with Tight settings (red), MtsslWizard with Loose settings (blue). The different shapes correspond to the different structure predictor tools: circle AlphaFold2, triangle OmegaFold, diamond X-ray (PDB: 4wh4) and square ESM-Fold. **a)** 6C and 28C MTSL, **b)** 6C and 28C MPSL, **c)** 6C and 28C IPSL, **d)** 6C and 28C IDSL.

1.11 Root mean square deviation (RMSD) values

To compare the overall shapes of the experimental and *in silico* distance distributions, the rmsd values were calculated with an in-house Matlab software (**Tables S12-S15**).

AlphaFold2

RMSD		MTSL		MPSL		IPSL		IDSL	
		6C	28C	6C	28C	6C	28C	6C	28C
**MMM**	T_{cryo}	0.0070	0.0159	0.0132	0.0072	0.0118	0.0082	0.0094	0.0193
	T_{amb}	0.0083	0.0149	0.0202	0.0075	0.0068	0.0081	0.0094	0.0191
**Wizard**	Tight	0.0247	0.0197	0.0182	0.0082	0.0121	0.0082	0.0190	0.0096
	Loose	0.0153	0.0170	0.0082	0.0057	0.0057	0.0057	0.0135	0.0065

Table S12 Root mean square deviation (rmsd) values of the modelled distances for both MMM and MtsslWizard at both different conditions (ambient and cryogenic temperature and Tight and Loose settings), for both GB1 constructs I6C/K28H/Q32H and I6H/N8H/K28C, each respectively with the four nitroxides (MTSL, MPSL, IPSL and IDSL), for the AlphaFold2 structure

OmegaFold

RMSD		MTSL		MPSL		IPSL		IDSL	
		6C	28C	6C	28C	6C	28C	6C	28C
**MMM**	T_{cryo}	0.0098	0.0169	0.0133	0.0074	0.0071	0.0088	0.0226	0.0190
	T_{amb}	0.0076	0.0147	0.0175	0.0121	0.0067	0.0079	0.0201	0.0188
**Wizard**	Tight	0.0245	0.0197	0.0135	0.0117	0.0099	0.001	0.0228	0.0070
	Loose	0.0166	0.0158	0.0075	0.0101	0.0057	0.0046	0.0130	0.0065

Table S13 Root mean square deviation (rmsd) values of the modelled distances for both MMM and MtsslWizard at both different conditions (ambient and cryogenic temperature and Tight and Loose settings), for both GB1 constructs I6C/K28H/Q32H and I6H/N8H/K28C, each respectively with the four nitroxides (MTSL, MPSL, IPSL and IDSL), for the OmegaFold structure

ESMFold

RMSD		MTSL		MPSL		IPSL		IDSL	
		6C	28C	6C	28C	6C	28C	6C	28C
**MMM**	T_{cryo}	0.0085	0.0137	0.0123	0.0058	0.0069	0.0066	0.0104	0.0118
	T_{amb}	0.0114	0.0139	0.0176	0.0055	0.0067	0.0066	0.0098	0.0108
**Wizard**	Tight	0.0246	0.0205	0.017	0.0120	0.0135	0.0078	0.0224	0.0102
	Loose	0.0165	0.0166	0.0084	0.0101	0.0057	0.0051	0.0120	0.0070

Table S14 Root mean square deviation (rmsd) values of the modelled distances for both MMM and MtsslWizard at both different conditions (ambient and cryogenic temperature and Tight and Loose settings), for both GB1 constructs I6C/K28H/Q32H and I6H/N8H/K28C, each respectively with the four nitroxides (MTSL, MPSL, IPSL and IDSL), for the ESMfold structure

X-ray 4wh4

RMSD		MTSL		MPSL		IPSL		IDSL	
		6C	28C	6C	28C	6C	28C	6C	28C
MMM	T_{cryo}	0.0099	0.0137	0.0151	0.0086	0.0077	0.009	0.0115	0.0184
	T_{amb}	0.0106	0.0139	0.0169	0.0133	0.0075	0.0081	0.0100	0.0185
Wizard	Tight	0.0239	0.0189	0.0036	0.0128	0.0103	0.0059	0.0201	0.0034
	Loose	0.0146	0.0165	0.0104	0.081	0.0051	0.0055	0.0129	0.0074

Table S15 Root mean square deviation (rmsd) values of the modelled distances for both MMM and MtsslWizard at both different conditions (ambient and cryogenic temperature and Tight and Loose settings), for both GB1 constructs I6C/K28H/Q32H and I6H/N8H/K28C, each respectively with the four nitroxides (MTSL, MPSL, IPSL and IDSL), for the X-ray crystallographic structure (PDB: 4wh4)

To better estimate which labelling approach globally predicts more consistently the experimental behaviour, we compared at the same time all the mean values of the distance distributions of both constructs with the four distinct nitroxide spin labels for a single *in silico* labelling approach, with the same experimental data, extracting the global rmsd values (**Table S16**). This procedure was repeated for the two labelling approaches and their respective conditions (ambient and cryogenic temperatures for MMM and Tight and Loose settings for MtsslWizard). All three different structure prediction methods (AlphaFold2, OmegaFold and ESMFold) and the X-ray crystallographic structure were analysed. The same procedure was employed to analyse the distribution widths (**Table S16**).

RMSD Global		AlphaFold2		OmegaFold		ESMFold		X-ray	
		Mean	Width	Mean	Width	Mean	Width	Mean	Width
**MMM**	T_{cryo}	0.219	0.077	0.267	0.071	0.156	0.069	0.233	0.071
	T_{amb}	0.233	0.071	0.252	0.071	0.173	0.068	0.242	0.082
**Wizard**	Tight	0.230	0.110	0.211	0.112	0.238	0.107	0.191	0.100
	Loose	0.125	0.051	0.127	0.048	0.137	0.054	0.111	0.047

Table S16 Global RMSD values, extracted by comparison between all the mean values (for both GB1 constructs and the four nitroxide labels) for a single labelling approach with the experimental values. Repeated for the two labelling approaches MMM and MtsslWizard with their respective conditions, cryogenic and ambient temperature for the former, and Tight and Loose settings for the latter. All predicted structures (AlphaFold2, OmegaFold, ESMFold) and the X-ray crystallographic structure have been considered. The same procedure was employed to analyse the distribution widths

II References

[1] K. Ackermann, J.L. Wort, B.E. Bode, Pulse dipolar EPR for determining nanomolar binding affinities, *Chem Commun,* **2022**; 58, 8790-8793, https://doi.org/10.1039/d2cc02360a.

[2] S. Ghosh, S. Saxena, G. Jeschke, Rotamer Modelling of Cu(II) Spin Labels Based on the Double-Histidine Motif, *Applied Magnetic Resonance,* **2018**; 49, 1281-1298, https://doi.org/10.1007/s00723-018-1052-8.
